# Small molecule-mediated activation of Notch signal transduction

**DOI:** 10.1038/s41419-026-09044-x

**Published:** 2026-07-02

**Authors:** Subhamita Dey, Benedetto Daniele Giaimo, Iva Katharina Zöllner, Qingwen Yang, Hina Zarrin, Jasmin Ballout, Martin Diener, Tobias Friedrich, Francesca Ferrante, Jan Dreute, M. Lienhard Schmitz, Astrid Weiss, Pierfrancesco Polo, Matthias Lauth, Bernd Gahr, Steffen Just, Marek Bartkuhn, Franz Oswald, Tilman Borggrefe

**Affiliations:** 1 Institute of Biochemistry, Justus-Liebig-University Giessen, Giessen, Germany; 2 Department of Internal Medicine I, Center for Internal Medicine, University Medical Center Ulm, Ulm, Germany; 3 Department of Internal Medicine II, Universities of Giessen and Marburg Lung Center, University Hospital Giessen, Justus Liebig University, Giessen, Germany; 4 Institute of Veterinary Physiology and Biochemistry, Justus-Liebig University, Giessen, Germany; 5 Biomedical Informatics and Systems Medicine, Justus-Liebig University, Giessen, Germany; 6 Center for Tumor and Immune Biology, Clinics for Gastroenterology, Endocrinology and Metabolism, Philipps University Marburg, Marburg, Germany; 7 Molecular Cardiology, Department of Internal Medicine II, Ulm University, Ulm, Germany

**Keywords:** Signal transduction, Non-small-cell lung cancer, Differentiation

## Abstract

The Notch signaling pathway is pivotal in regulating cell differentiation, stem cell maintenance and oncogenesis. While several Notch inhibitors have been developed, effective small molecule activators are scarce. Here, we identify a small molecule that robustly activates Notch signaling, leading to significant upregulation of Notch target genes in several human cell lines and in *Danio rerio* embryos. Mechanistically, this compound induces the expression of activated NOTCH3 protein via a cryptic internal promoter within the *NOTCH3* locus. Notch induction is markedly diminished upon genetic depletion of NOTCH3 or the canonical Notch transcription factor RBPJ. Furthermore, we demonstrate that the compound inhibits complex I of the respiratory chain. This is accompanied by a raise of cytosolic Ca^2+^ concentration, which is pivotal for the induction of NOTCH3. Functionally, the Notch inducer enforces cell-cycle arrest and promotes terminal differentiation in acute myeloid leukemia (AML) cells. These findings suggest that this small molecule serves as a potent tool for modulating Notch signaling and holds therapeutic potential for Notch-responsive malignancies.

## Introduction

Notch signaling is a conserved signal transduction pathway regulating embryonic as well as post-natal developmental processes, including stem cell maintenance and differentiation [1, 2]. It has been implicated in carcinogenesis, such as in lung cancer and leukemia [3, 4]. At the molecular level, NOTCH proteins are a family of type 1 transmembrane proteins where ligand binding leads to their proteolytic processing by a γ-secretase containing complex releasing the NOTCH intracellular domain (NICD) that migrates to the nucleus. The NICD associates with transcription factor RBPJ (recognition binding protein-J), assembles a coactivator complex and hence activates transcription. Important target genes are helix-loop-helix transcription factors, such as *HES1, HEY1, HEY2*, and *HES4*, but also NOTCH pathway components per se, like the *NOTCH1* and *NOTCH3* genes.

Several groups have developed Notch modulators, in particular Notch inhibitors, both for regenerative as well as for cancer therapies [5, 6]. Inhibition of the γ-secretase mediated release of the NICD leads to efficient downregulation of Notch target gene expression decreasing neurospheres formation in malignant gliomas [7] and sensitizing hepatocellular carcinoma cells to apoptosis [8]. However, γ-secretase inhibitors (GSIs) affect all NOTCH receptors and cause severe gastrointestinal problems [9]. To circumvent the cytotoxic effects associated with GSIs, monoclonal antibodies have been developed that target individual NOTCH receptors [10]. There have been also alternative Notch inhibitors disrupting the NICD-associated coactivator complex [11] or other small molecules like CB103 [12] and RIN1 [13], interfering with the assembly of the Notch transcription complex.

Less is known about Notch pathway activators, which could be used to study the impact of Notch signaling on disease onset and progression. Notch activating agents offer the potential for differentiation therapy for acute myeloid leukemia (AML) patients [14], regenerative therapy to promote osteogenesis in osteoporosis patients [15] and for ex vivo applications such as the expansion of precursor T-cells [16]. Recently, a potent biological Delta-like canonical Notch ligand 4 (DLL4) variant fragment (Delta^MAX^) has been developed, which could be potentially used in T-cell related applications [17]. Alternatively, combining DNA with clusters of Notch ligand JAG1 can be used to activate NOTCH [18]. An agonistic antibody has also been described for NOTCH3, targeting the negative regulatory region within its extracellular domain [19].

The small molecule isoxazole 9 (hereafter referred to as ISX) has been originally characterized in stem cell differentiation assays [20]. ISX potentiates the expression of the neuronal transcription factor *NEUROD1* and also enhances glucose-stimulated insulin secretion in ex vivo cultures of human pancreatic islet cells [21]. Mechanistically, ISX has been shown to trigger a rise in cytosolic calcium (Ca^2+^) in neurogenesis [20] and this was further supported by activation of Ca^2+^-dependent kinases and of NFAT (nuclear factor of activated T cells) proteins in pancreatic beta-cells [22]. In the context of carcinogenesis, ISX potently abrogates neuroblastoma cell proliferation by modulating the Hedgehog signaling pathway [23].

In this study, we characterize ISX as a novel Notch pathway activator in several different contexts. The ISX-mediated activation of Notch target gene expression depends on NOTCH3 and the transcription factor RBPJ. The versatility of this novel Notch activator is demonstrated by terminal differentiation of AML cells.

## Results

### ISX-mediated activation of Notch signaling

**Fig. 1:**
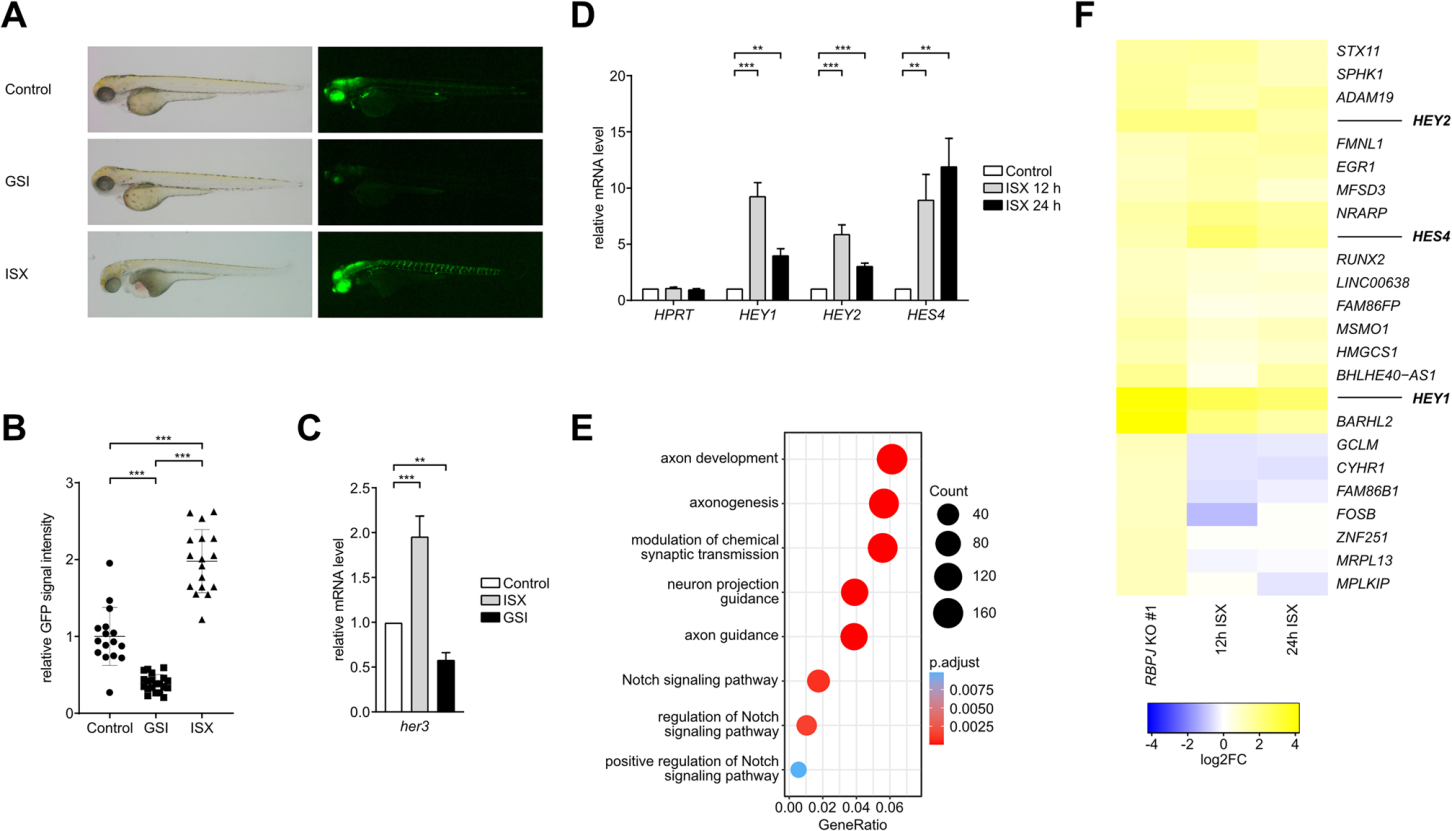
ISX induces the expression of Notch in zebrafish and in H1299 lung cancer cells. Representative images (**A**) and quantification (**B**) of 72 hpf (hours post fertilization) old Tg[12xRBP:EGFP] embryos, treated with DMSO (Control), GSI or ISX. GSI treatment leads to reduced Notch reporter activity in the zebrafish line Tg[12xRBP:EGFP], whereas treatment with ISX leads to an upregulation of Notch reporter activity. Data are presented as mean ± SD of three independent experiments (***P < 0.001; bar graph; 500 µm). **C** Notch target genes are upregulated by ISX treatment. Gene expression of the Notch target gene *her3* is upregulated after 24 h of treatment with ISX and downregulated upon treatment with GSI (**P < 0.01; ****P < 0.0001). **D** ISX treatment leads to upregulation of Notch target genes in human lung cancer cells (H1299). H1299 cells were treated with 20 μM ISX or DMSO as a control for 12 or 24 h. Total RNA was purified, reverse transcribed and Notch target genes expression was analyzed by RT-qPCR using gene-specific primers for *HPRT*, *HEY1*, *HEY2*, and *HES4*. Data were normalized to the housekeeping gene *GAPDH* and represent the mean ± SD of three independent experiments (**P < 0.01, ***P < 0.001, unpaired Student’s t-test). **E, F** H1299 cells were treated with 20 μM ISX or DMSO as a control for 12 or 24 h, RNA was purified and analyzed by deep sequencing. **E** More Notch-related gene ontology (GO) terms were identified among the genes commonly upregulated upon 12 and 24 h of ISX treatment in H1299 cells. **F** RBPJ target genes are upregulated by ISX. Heat map showing the effects of ISX treatment (12 or 24 h) on genes bound by RBPJ and upregulated upon RBPJ depletion in H1299 cells.

In higher eukaryotes, active Notch signaling is known to induce differentiation, for example during neurogenesis. When searching for pharmacological Notch modulators, we considered the known neural inducer ISX, since it has been used by several groups in differentiation assays [20, 24]. We first used the zebrafish (*Danio rerio*) Notch reporter line Tg[12xRBPJ:EGFP], which is easily amenable to small molecule treatments. GFP expression in 72 hpf (hours post fertilization) embryos, treated for 48 h, was overall reduced upon treatment with Notch γ-secretase inhibitor (GSI) and increased upon treatment with small molecule ISX compared to control (DMSO-treated) embryos (Fig. 1A, B). Specifically, ISX led to a strong increase of GFP expression visible in the head and heart region and most prominently in the vasculature whereas treatment with Notch inhibitor GSI abrogated the Notch induction (Fig. 1A, B and Supplementary Fig. 1A). The ISX effects on Notch reporter expression were dose-dependent with strong effects at 10 μM and 5 μM and intermediate at 2.5 μM and 1 μM, whereas no GFP-induction was observed at 0.5 μM (Supplementary Fig. 1A). At the molecular level, the expression of Notch target gene *her3* was upregulated by treatment with ISX and downregulated with GSI (Fig. 1C). Thus, ISX induces a specific Notch-dependent response in zebrafish.

Subsequently, we investigated whether ISX is also able to induce Notch target gene expression in a mammalian setting. We treated non-small cell lung cancer H1299 cells for 12 or 24 h with ISX and observed a strong upregulation of the helix-loop-helix factors encoding *HEY1, HEY2*, and *HES4* genes (Fig. 1D). Furthermore, the ISX- mediated induction of Notch target genes is detectable in other human cell lines

including H69 small cell lung carcinoma cells (Supplementary Fig. 1B). In order to evaluate whether the increased expression of Notch target genes upon ISX treatment is due to increased Notch signaling, we performed Western blotting with an anti- NOTCH1 antibody and observed a significant increase in cleaved active NOTCH1 (NICD1) protein both at 12 and 24 h after ISX treatment (Supplementary Fig. 1C). The detection of an increase in the cleaved and active NOTCH1 protein upon ISX treatment would suggest an increased activation of the pathway. In order to test this, we performed RNA-Seq analysis both at 12 and 24 h of treatment with ISX. We observed that the majority of deregulated genes are upregulated and there is a significant overlap for upregulated and a good overlap for downregulated genes (Supplementary Fig. 1D and Supplementary Table 1). When performing unbiased gene ontology (GO) analyses of the ISX-induced genes, we observed overrepresentation of several Notch-related terms (Fig. 1E and Supplementary Table 2). Similarly, a Gene Set Enrichment Analysis (GSEA) identified the “Notch signaling pathway” both at 12 and 24 h of ISX treatment (Supplementary Fig. 1E and Supplementary Table 3). In order to investigate the effects of ISX on Notch target genes in H1299 cells, we depleted the transcription factor RBPJ, which is the key component of the machinery mediating the Notch transcriptional response (Supplementary Fig. 2A). We observed dysregulation of several well-known Notch target genes such as *HEY1, HEY2*, and *HES4* upon RBPJ depletion in H1299 cells (Supplementary Fig. 2B). Subsequently, we focused on RBPJ KO clone #1 and performed RNA-Seq and ChIP-Seq with anti- RBPJ antibodies to identify bona fide RBPJ targets in H1299 cells. The RBPJ binding motif was promptly identified in our ChIP-Seq analysis (Supplementary Fig. 2C) and the signal was lost in the RBPJ-depleted cells (Supplementary Fig. 2D, E and Supplementary Table 4), validating the specificity of our ChIP-Seq experiments. Once we defined Notch target genes as those genes upregulated upon RBPJ depletion and bound by RBPJ, we observed that most of them are significantly upregulated by ISX treatment (Fig. 1F). Together, this suggests that ISX treatment leads to increased Notch signaling in human lung cancer cell lines.

### ISX strongly induces expression of NOTCH3 via a cryptic internal promoter

**Fig. 2:**
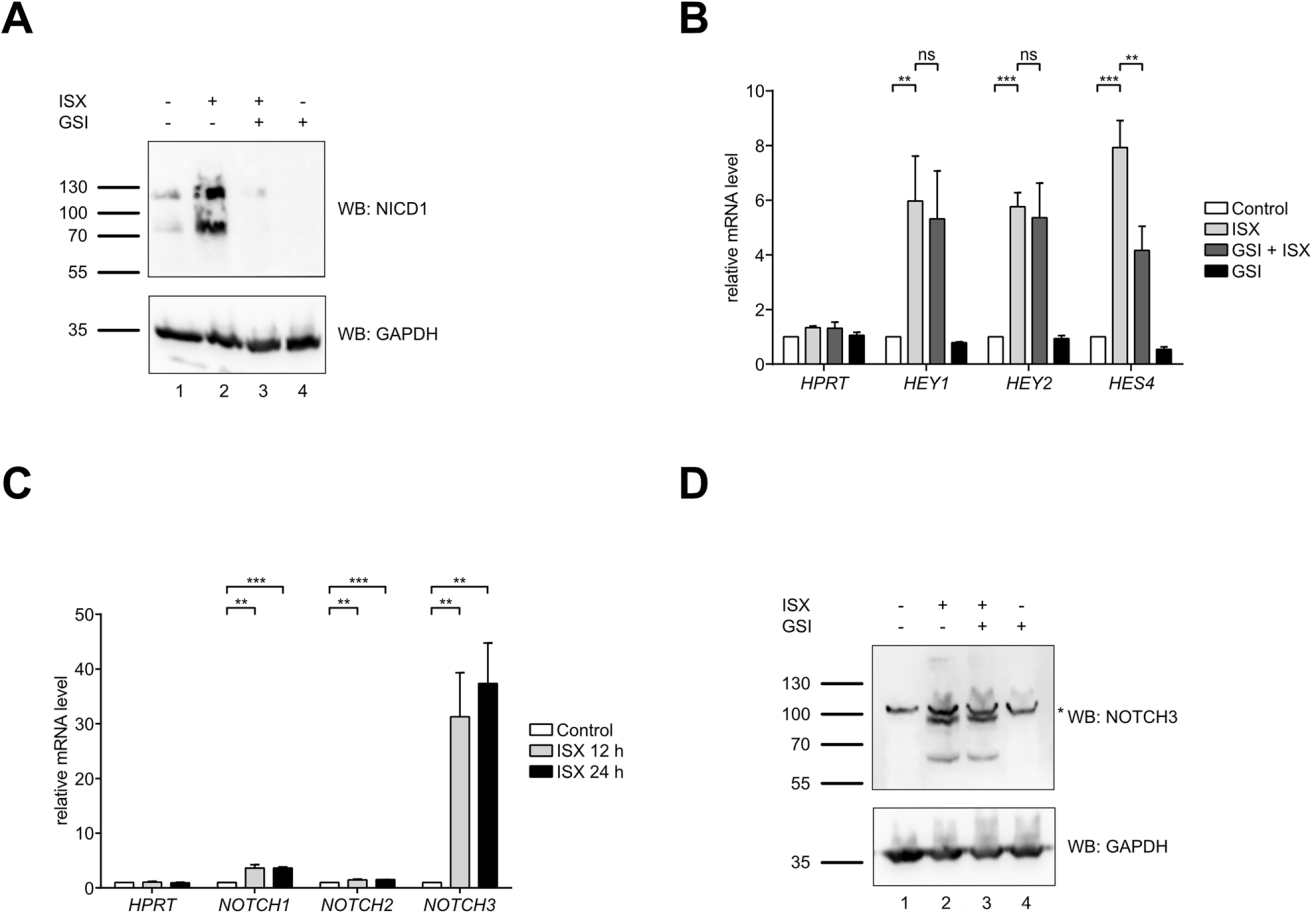
ISX-mediated activation of Notch target genes is independent of NOTCH receptor processing. **A** γ-secretase inhibitor (GSI) treatment reduces ISX-induced NICD1 protein. H1299 cells were treated with 20 µM ISX or DMSO as a control for 12 h in presence of GSI (23.1 µM DAPT) to block NOTCH receptor processing. Whole cell extracts (WCE) were analyzed by Western blotting versus NICD1 or GAPDH as a loading control. **B** Inhibition of NOTCH receptor processing does not abrogate Notch target genes induction by ISX. H1299 cells were treated with 20 µM ISX or DMSO as a control for 12 h in presence of GSI (23.1 µM DAPT) to block NOTCH receptor processing. Total RNA was purified, reverse transcribed and Notch target genes expression was analyzed by RT-qPCR using gene-specific primers for *HPRT*, *HEY1*, *HEY2*, and *HES4*. Data were normalized to the housekeeping gene *GAPDH* and represent the mean ± SD of three independent experiments (**P < 0.01, ***P < 0.001, ns not significant, unpaired Student’s t-test). **C** ISX induces expression of *NOTCH1*, *NOTCH2* and *NOTCH3* genes in H1299 cells. H1299 cells were treated with 20 µM ISX or DMSO as a control for 12 or 24 h. Total RNA was purified, reverse transcribed and Notch target genes expression was analyzed by RT-qPCR using gene-specific primers for *HPRT*, *NOTCH1* and *NOTCH3*. Data were normalized to the housekeeping gene *GAPDH* and represent the mean ± SD of three independent experiments (**P < 0.01, ***P < 0.001, unpaired Student’s t-test). **D** ISX-induced NICD3 is not susceptible to γ-secretase inhibitor (GSI) treatment in H1299. H1299 cells were treated with 20 µM ISX or DMSO as a control for 12 h in presence of GSI (23.1 µM DAPT) to block NOTCH receptor processing. Whole cell extracts (WCE) were analyzed by Western blotting versus NOTCH3 or GAPDH as a loading control.

Subsequently, we investigated whether Notch induction upon ISX treatment depends on γ-secretase activity in H1299 cells: While the ISX-mediated activation of NOTCH1 was abolished by co-treatment with GSI (Fig. 2A). However, the induction of Notch target genes was not affected (Fig. 2B), as *HEY1* and *HEY2* remained induced even upon GSI-treatment and *HES4* was partially downregulated. As a consequence, we hypothesized that another Notch receptor apart from NOTCH1 may be activated in a γ-secretase independent manner. When looking at mRNA levels of the different Notch receptors upon ISX, it was evident that *NOTCH3* upregulation is the strongest of all the NOTCH receptor encoding genes analyzed (Fig. 2C). Subsequently, we observed that the activated form of NOTCH3 was clearly induced upon ISX treatment and that co-treatment with GSI did not abrogate this effect (Fig. 2D). As a further validation in primary cells, we treated human primary pulmonary artery smooth muscles cells (hPASMCs) with ISX. We observed that *NOTCH3* but not *NOTCH1* and *NOTCH2* is significantly induced by ISX (Supplementary Fig. 3A). In line with this, the cleaved and active NOTCH3 protein is detected upon ISX treatment and Notch target genes *HES4* and *HEYL* are efficiently induced by ISX treatment (Supplementary Fig. 3B, C). Together, ISX treatment leads to induction of active NOTCH3 in a γ-secretase- independent manner leading to upregulation of Notch target genes.

**Fig. 3:**
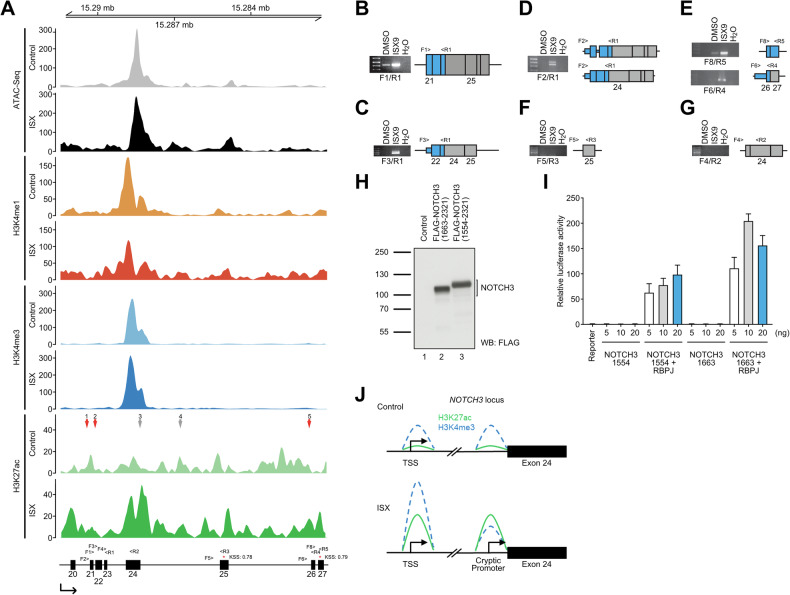
ISX induces an alternative transcription start site (TSS) within the *NOTCH3* locus. **A** H1299 cells were treated with 20 μM ISX or DMSO as a control for 12 h and the chromatin configuration was analyzed via ATAC-Seq or ChIP-Seq versus H4K4me1, H3K4me3 or H3K27ac. An alternative transcription starting site (TSS) before exon 24 (see scheme at the bottom) is indicated by presence of a typical core promoter mark H3K4me3 (blue) as well as enhancer mark H3K4me1 (in orange/red) and chromatin accessibility as detected by ATAC-Seq. ISX treatment leads to increased H3K27ac (green) at this alternative TSS. **B–G** RT-PCR experiments determining the exon-exon and intron-exon junctions within the *NOTCH3* locus upon ISX treatment; Primer location is indicated in the scheme in (**A**). Control exons 21–25 transcript is induced by ISX (**B**, **C**, **E**, upper panel) as well as intron- exon fragments amplified with F2/R1 (**D**, upper and lower band) and F6/R4 (**E**, lower panel) but not for exon 24 and 25 (**F**, **G**). **H** NOTCH3 proteins starting at the methionine 1554 within exon 25 (lane 3) or methionine 1663 within exon 27 (lane 2) are properly translated and (**I**) the NOTCH3 a.a. 1554-2321 as well as NOTCH3 a.a. 1663-2321 protein fragments are able to activate transcription in Notch luciferase reporter assays. **J** Summary of changes in chromatin marks (H3K4me3 in blue; H3K27ac in green) at the *NOTCH3* locus after ISX treatment in H1299 cells: H3K27 acetylation is strongly upregulated both at the TSS and at the cryptic promoter.

One possible scenario that could explain the γ-secretase-independent activation of NOTCH3 is the presence of a cryptic promoter activated upon ISX treatment, resulting in an active C-terminal NICD3 fragment. In order to test this hypothesis, we first analyzed the chromatin configuration of the *NOTCH3* locus by ChIP-Seq and ATAC- Seq in H1299 cells upon ISX treatment for 12 h. We detected an H3K4me3 enrichment between exons 23 and 24 of the *NOTCH3* locus that colocalizes with a signal for chromatin accessibility as revealed via ATAC-Seq (Fig. 3A and Supplementary Fig. 4A), suggesting the presence of an internal cryptic promoter. Of note, ISX treatment increases chromatin accessibility and H3K4me3 and the TSS but has no impact on the intragenic peaks located between exons 23 and 24 (Fig. 3A and Supplementary Fig. 4A). Interestingly, H3K4me1 is partially influenced by ISX treatment but the most important observation is an increase in H3K27ac upon ISX treatment both at the TSS and at the intragenic promoter (Fig. 3A and Supplementary Fig. 4A), suggesting that ISX treatment leads to the activation of both promoters. The presence of this intragenic promoter would suggest the transcription of novel mRNAs stemming from the intronic site close to the H3K4me3 peak between exons 23 and 24. Thus, we scanned exon-exon junction and intron-exon transcripts after ISX treatment to determine putative transcripts from this cryptic intragenic promoter. As expected, several exon-exon spanning transcripts are induced: Exons 21–23 (Fig. 3B), exons 22–23 (Fig. 3C). Importantly, there are also transcripts detectable with intron/exon primers upstream of exon 21 (Fig. 3D, amplified with primers F2 and R1), upstream of exon 26 (Fig. 3E, lower panel, amplified with F6 and R4) but not upstream of exon 25 (Fig. 3F, amplified with F5 and R3) or exon 24 (Fig. 3G, amplified with F4 and R2). Based on these results, we searched for a Kozac sequence downstream of the cryptic promoter. We found potential translation initiation sites within exon 25 and 27 (Fig. 3A, lower scheme, represented with asterisks) showing a KSS (Kozac Similarity Score) of 0.78 and 0.79, respectively. Subsequently we tested the transcriptional activity of proteins encoded by these transcripts generated by the intragenic promoter. We expressed NICD3 proteins from residue M1554 located in exon 25 or M1663 located in exon 27 and tested their expression and function. Both truncated C-terminal fragments of NICD3 are well expressed, localize to the nucleus and are strong activators in Notch reporter assays (Fig. 3H, I and Supplementary Fig. 4B, C). Interestingly, analyzing ENCODE ChIP-Seq data, we observed that H3K4me3 is enriched at the cryptic promoter of the *NOTCH3* gene in hSMCs but not in human CD3^+^ T-cells (Supplementary Fig. 5), suggesting cell-type-specific differences. In conclusion, ISX is able to activate a cryptic promoter which produces *NOTCH3* transcripts (Fig. 3J) that, after translation of N-terminally truncated proteins, are able to activate a NOTCH3- driven response.

### NOTCH3 and RBPJ are necessary for Notch-induction

**Fig. 4:**
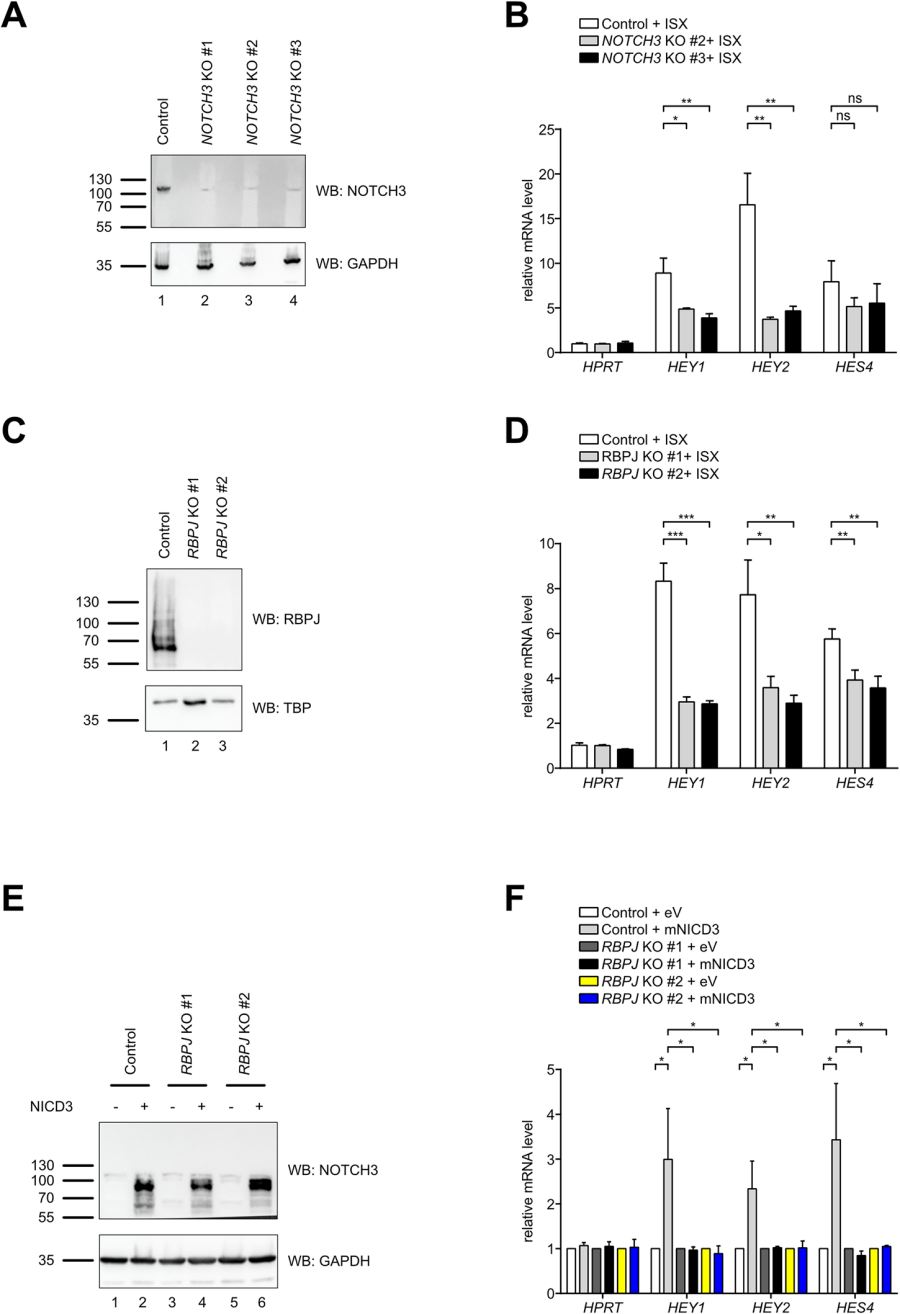
NOTCH3 and RBPJ are required for ISX-mediated induction of Notch target genes. **A**, **B** Depletion of NOTCH3 in H1299 cells leads to downregulation of the ISX- mediated induction of Notch target genes. Depletion of NOTCH3 in H1299 cells was achieved making use of the CRISPR9/Cas9 technology and the depleted cells were eventually treated with ISX for 12 h. **A** Whole cell extract (WCE) from NOTCH3-depleted and control H1299 cells was analyzed by Western blotting versus NOTCH3 or GAPDH as a loading control. **B** Total RNA was purified from NOTCH3-depleted and control H1299 cells treated for 12 h with ISX, the RNA was reverse transcribed and Notch target genes expression was analyzed by RT-qPCR using gene-specific primers for *HPRT*, *HEY1*, *HEY2*, and *HES4*. Data were normalized to the housekeeping gene *GAPDH* and represent the mean ± SD of three independent experiments (*P < 0.05, **P < 0.01, ns not significant, unpaired Student’s t- test). **C**, **D** Depletion of RBPJ leads to downregulation of Notch target genes in H1299 cells. Depletion of RBPJ in H1299 cells was achieved making use of the CRISPR9/Cas9 technology and the depleted cells were eventually treated with ISX for 12 h. **C** Nuclear extract (NE) from RBPJ-depleted and control H1299 cells was analyzed by Western blotting for RBPJ or TBP as a loading control. **D** Total RNA was purified from RBPJ-depleted and control H1299 cells treated for 12 h with ISX, the RNA was reverse transcribed and Notch target genes expression was analyzed by RT-qPCR using gene-specific primers for *HPRT*, *HEY1*, *HEY2*, and *HES4*. Data were normalized to the housekeeping gene *GAPDH* and represent the mean ± SD of three independent experiments (*P < 0.05, **P < 0.01, ***P < 0.001, unpaired Student’s t-test). **E**, **F** The NOTCH3-dependent induction of Notch target genes is RBPJ-dependent in H1299 cells. NICD3 was ectopically expressed in wildtype (control) or RBPJ-depleted H1299 cells. Control and RBPJ-depleted clones #1 or #2 were transfected with NICD3 or empty vector as a control. **E** Whole cell extract (WCE) was analyzed by Western blotting for NOTCH3 or GAPDH as a loading control. **F** Total RNA was purified, reverse transcribed and Notch target gene expression was analyzed by RT-qPCR using gene-specific primers for *HPRT*, *HEY1*, *HEY2*, and *HES4*. Data were normalized to the housekeeping gene *GAPDH* and represent the mean ± SD of three independent experiments (*P < 0.05, unpaired Student’s t-test).

To further analyze the molecular mechanism of ISX-mediated effects on Notch signaling, we performed loss- and gain-of-function experiments in H1299 cells. Using CRISPR/Cas9 technology, we depleted NOTCH3 in H1299 human lung cancer cells (Fig. 4A and Supplementary Fig. 6) and observed that NOTCH3 depletion significantly impairs the ISX-mediated induction of *HEY1, HEY2* but not of *HES4* (Fig. 4B); It is possible that induction of *HES4* is more NOTCH1 dependent. In order to investigate whether the ISX-mediated induction of Notch target genes depends on canonical transcription factor RBPJ that interacts with any of the activated NICDs, we made use of our RBPJ-depleted H1299 cells. We observed that the ISX-mediated induction of *HEY1*, *HEY2*, and *HES4* is impaired in RBPJ-depleted cells (Fig. 4D). As a further validation, we overexpressed activated NOTCH3 (NICD3) in both control and RBPJ- depleted H1299 cells (Fig. 4E) and observed a strong RBPJ-dependent, NICD3-mediated activation of *HEY1, HEY2*, and *HES4* (Fig. 4F). Thus, we conclude that activated NOTCH3 is required for the ISX-mediated induction of Notch target genes, and this process is dependent on the canonical transcription factor RBPJ.

### ISX decreases mitochondrial complex I activity and increases cytosolic Ca^2+^ resulting in *NOTCH3* activation

**Fig. 5:**
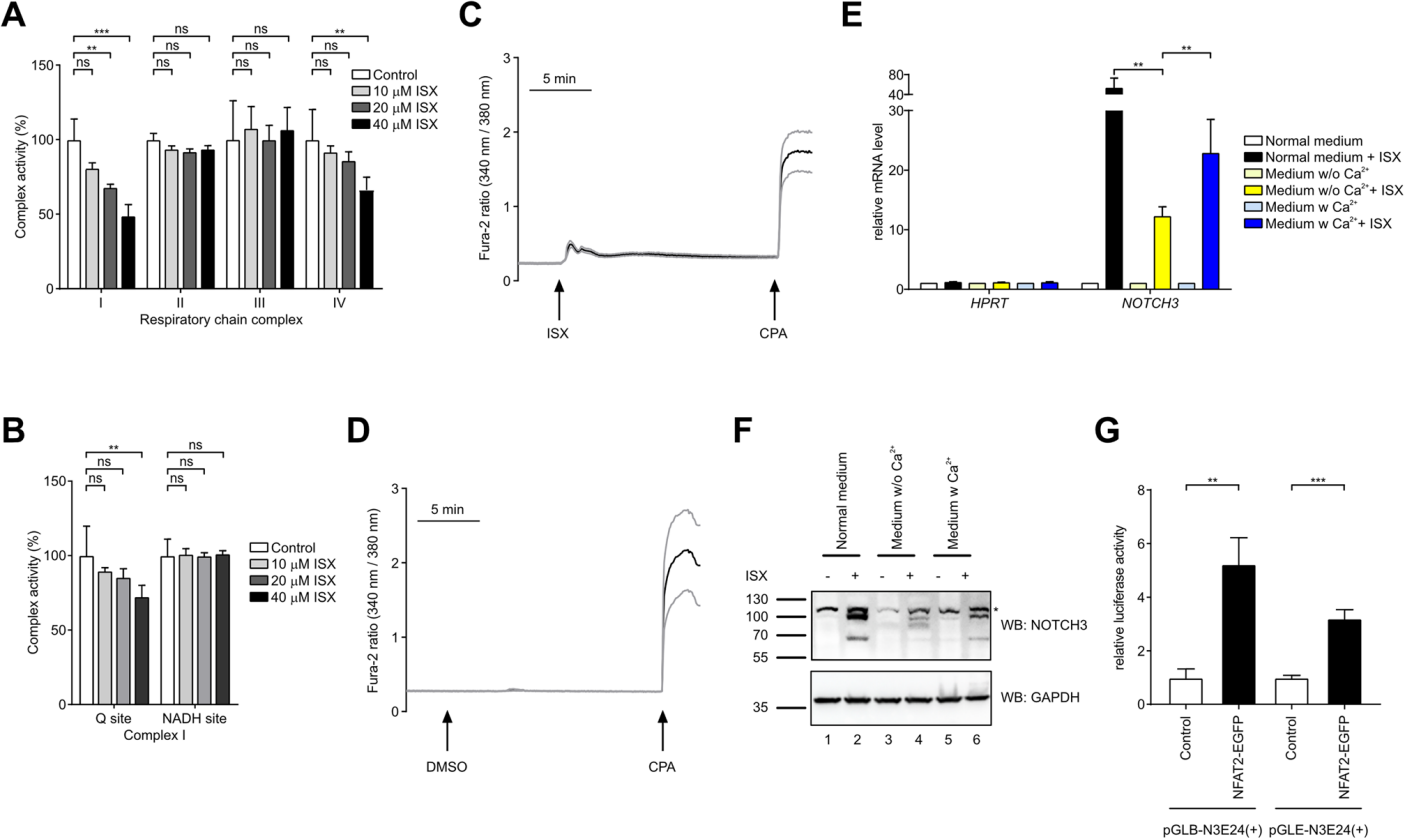
ISX decreases mitochondrial complex I activity and induces a Ca^2+^-dependent activation of NOTCH3. **A**, **B** Dose-dependent inhibition of respiratory chain complex I. **A** Bovine heart mitochondria were analyzed using respiratory chain complex specific activity assays. Assays were performed in presence of the indicated ISX concentrations or DMSO as a control and analyzed spectrophotometrically. Shown is the mean ± SD of three independent experiments, two-way ANOVA with Dunnett’s multiple comparisons test. **B** Bovine heart mitochondria were analyzed for the activities of the Q- and NADH-site of complex I in presence of the indicated ISX concentrations or DMSO as a control. Shown is the mean ± SD of three independent experiments, two-way ANOVA with Dunnett’s multiple comparisons test. **C**, **D** The ISX-mediated NOTCH3 induction requires Ca^2+^ and is associated with activation of Ca^2+^- dependent kinases. ISX increases cytosolic Ca^2+^ concentration in H1299 cells. Cytosolic Ca^2+^ level was measured as a quantification of Fura-2 (340/380) ratios from one respective experiment shown as mean (black line) ± SEM (gray area) after stimulation with ISX or DMSO as a control. CPA was used as a control for cell viability. See also exact numbers of cells and quantitation in Supplementary Table 5. **E**, **F** ISX-driven upregulation of *NOTCH3* is Ca^2+^- dependent. H1299 cells were treated with ISX for 12 h in normal medium, medium depleted of Ca^2+^ (w/o) or medium depleted of Ca^2+^ and re-supplemented with Ca^2+^ (w). **E** Notch target genes expression was analyzed by RT-qPCR using gene-specific primers for *HPRT* and *NOTCH3*. In Ca^2+^-free medium (w/o, yellow bar) NOTCH3 expression is reduced, whereas upon re-addition of 1 mM Ca^2+^ *NOTCH3* expression is restored (purple bar). Data were normalized to the housekeeping gene *GAPDH* and represent the mean ± SD of five independent experiments (**P < 0.01, unpaired Student’s t-test). **F** Whole cell extract (WCE) was analyzed by Western blotting for NOTCH3 or GAPDH as a loading control. **G** Luciferase assay using *NOTCH3* intronic exon 24-25 fragment shows NFAT stimulatory activity (see also Supplementary Fig. 10C) upon overexpression of NFAT2 in HeLa cells in presence of PMA/ionomycin.

ISX has been implicated in modulating mitochondrial activity, at least in hybrid molecule approaches [25]. To directly test a possible effect of ISX on the activity of the respiratory chain complexes, we used isolated bovine heart mitochondria. Increasing amounts of ISX significantly reduced complex I activity but not complex II, III or IV activity (Fig. 5A, B). Inhibition of complex I can result in the release of Ca^2+^ into the cytoplasm [26]. ISX treatment has been previously reported to lead to increased cytosolic Ca^2+^ levels [20]. In order to test whether Ca^2+^ levels are influenced by ISX, we loaded H1299 cells with the Ca^2+^-sensitive fluorescent dye Fura-2 and stimulated with ISX. The SERCA inhibitor, cyclopiazonic acid (CPA), was used as viability control at the end of each experiment. ISX induced a rise in Fura-2 ratio within 5 min in 56% (65 out of 116) of analyzed cells (Fig. 5C and Supplementary Table 5), whereas only 1% (1 out of 83 cells) responded to DMSO, used as a control. No significant differences were observed in the ΔFura-2 ratio to CPA in cells pretreated with ISX in comparison to DMSO-treated cells confirming that ISX did not reduce the viability of the H1299 cells (Fig. 5C, D and Supplementary Table 5). In order to find out whether the ISX-induced increase in cytosolic Ca^2+^ concentration depends mainly on the presence of Ca^2+^ in the extracellular milieu, we used the Ca^2+^-free Tyrode solution. Under these conditions, the response to ISX was reduced by ~40%, whereas the number of responding cells did not change significantly (Supplementary Table 5). Unexpectedly, more cells reacted to DMSO under Ca^2+^-free conditions. Even the ΔFura-2 ratio to CPA was. reduced by about half in the DMSO group and by 75% when cells were pretreated with ISX (Supplementary Table 5).

As ISX evoked an increase in cytosolic Ca^2+^, we also investigated whether ISX regulates the activity of protein kinases (including Ca^2+^-dependent kinases) by using a peptide array chip strategy (PamStation). We observed a significant increase in activity of several Ca^2+^-dependent kinases in ISX-treated cells compared to the DMSO control (Supplementary Fig. 7, Supplementary Tables 6 and 7). In particular, we observed increased activity for the serine/threonine DCAMKL1, DAPK2, and ERK1 kinases (Supplementary Figs. 8, 9A and Supplementary Table 6). There were also multiple kinases for which a decrease of activity was observed after 30 min of ISX treatment, namely SRC, EPHA3, and HCK (Supplementary Fig. 8). These data were further supported by Western blot experiments that show a significant increase in phospho-ERK1 upon 10 and 30 min of ISX treatment in H1299 cells (Supplementary Fig. 9B). Of note, the increase in the phosphorylation of ERK1 is not detectable at 12 h of ISX treatment (Supplementary Fig. 9B), suggesting that this ISX-induced phosphorylation is a quick and transient effect. As a next step into the characterization of the effects of ISX on the Notch signaling pathway, we investigated whether the ISX-mediated NOTCH3 induction is Ca^2+^-dependent. We observed that both *NOTCH3* transcript (Fig. 5E) and NOTCH3 protein (Fig. 5F) are induced by ISX in a Ca^2+^-dependent manner. This was also reflected on the expression of Notch target genes such as *HES4* (Supplementary. 9C).

To investigate whether Ca^2+^-induction is sufficient to mimic ISX effects in H1299 cells, we tested alternative Ca^2+^-inducing agonists, namely histamine, which is known to stimulate cells via Gq protein-coupled histamine receptors [27]. In Fura-2-loaded H1299 cells, histamine evoked a similar Ca^2+^ increase as ISX (Supplementary Fig. 9D). From 120 cells tested, 62 (52%) responded with an increase in the Fura-2 ratio signal. Again, the ΔFura-2 ratio of the viability control CPA was not reduced (*n* = 120/120). While a clear increase in cytosolic Ca^2+^ concentration was detected in Fura-2 loaded Ca^2+^ imaging experiments, there was no increase in *NOTCH3* transcript levels (Supplementary Fig. 9D, E). Given that NFAT has been proposed to regulate the expression of *NOTCH3* [22], we hypothesized that NFAT (nuclear factor of activated T- cells) transcription factors may be activated by Ca^2+^ and consequently activate the cryptic promoter of the *NOTCH3* gene.

NFAT1 protein levels increase upon ISX treatment in H1299 cells, hPASMCs as well as in acute myeloid leukemia (AML) THP1 and MOLM14 cells (Supplementary Fig. 10A). Furthermore, NFAT4 and NFAT5 bind close to the cryptic promoter (exon-24) within the *NOTCH3* locus (Supplementary Fig. 10B). Subsequently, we performed luciferase assays to analyze the intronic region between exon 24 and 25 of the *NOTCH3* locus harboring several NFAT consensus sites (Supplementary Fig. 10C). The intronic fragments harbors some transcriptional activity even in the absence of NFAT1 overexpression, or induction with ISX or PMA/ionomycin (Supplementary Fig. 10D). As expected, NFAT2-EGFP expression is cytoplasmic and only enters the nucleus upon ISX or PMA/ionomycin treatment (Supplementary Figure 10E). Importantly, overexpression of NFAT2 in the presence of PMA/ionomycin activates transcription of the internal *NOTCH3* cryptic promoter as measured in luciferase assays (Fig. 5G).

Together, our results suggest that Ca^2+^-induction is necessary, but not sufficient for the ISX-mediated upregulation of NOTCH3 signaling and that the cryptic promoter within the *NOTCH3* gene is regulated by NFAT transcription factors.

### Notch-activator strongly induces terminal differentiation of AML cells

**Fig. 6:**
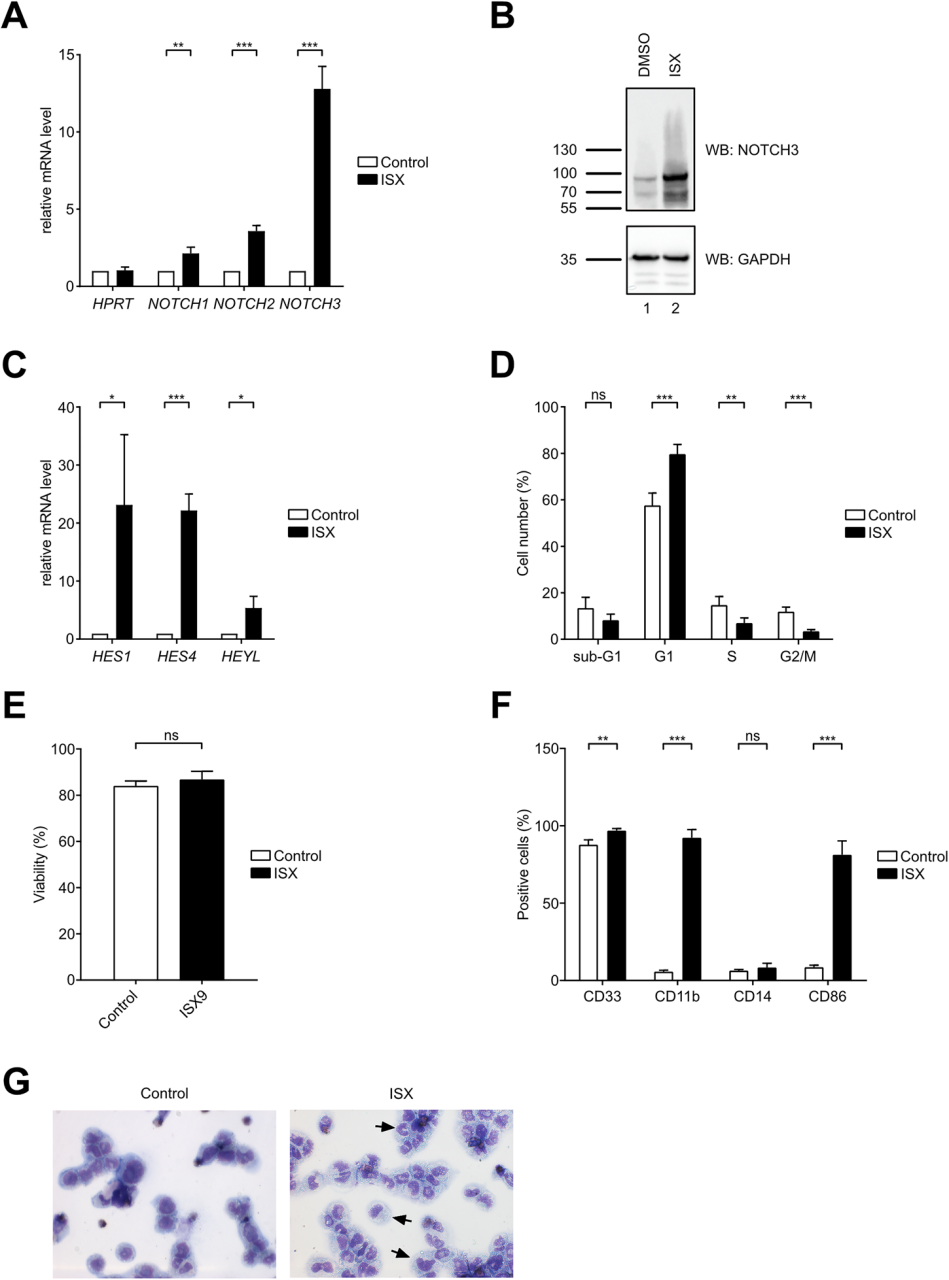
ISX promotes cell cycle arrest and differentiation in AML cells. **A**–**C** THP1 cells were treated with 20 µM ISX or DMSO as a control for 24 h. **A** Total RNA was isolated, reverse transcribed and Notch target genes expression was analyzed by RT-qPCR using gene-specific primers for *HPRT*, *NOTCH1*, *NOTCH2*, and *NOTCH3*. Data were normalized to the housekeeping gene *GAPDH* and represent the mean ± SD of three experiments (**P < 0.01, ***P < 0.001, unpaired Student’s t-test). **B** Whole cell extract (WCE) was analyzed by Western blotting versus NOTCH3 or GAPDH as a loading control. **C** Total RNA was purified from THP1 cells, reverse transcribed and Notch target genes expression was analyzed by RT- qPCR using gene-specific primers for *HES1*, *HES4* and *HEYL*. Data were normalized to the housekeeping gene *GAPDH* and represent the mean ± SD of three experiments (*P < 0.05, ***P < 0.001, unpaired Student’s t-test). **D**–**F** THP1 cells were treated with 10 μM ISX or DMSO as a control for 48 h. **D** Cell cycle progression of THP1 cells is arrested by ISX. THP1 cells were fixed and then incubated with DAPI. Cell cycle dynamics of the ISX and DMSO- treated THP1 cells were determined by the DNA content detected by DAPI staining. Shown is the mean ± SD of four independent experiments (**P < 0.01, ***P < 0.001, ns not significant, unpaired Student’s t-test). **E** THP1 cell viability remains unaffected upon ISX treatment. THP1 cells were stained with DAPI and acridine orange and the viability was determined by NucleoCounter NC 3000. Shown is the mean ± SD of 5–6 independent experiments (ns = not significant, unpaired Student’s t-test). **F** ISX strongly promotes the surface expression of differentiation markers in THP1 cells. Levels of surface antigens CD33, CD11b, CD14, and CD86 were analyzed by flow cytometry. Shown is the mean ± SD of three to six independent experiments (**P < 0.01, ***P < 0.001, ns not significant, unpaired Student’s t-test). **G** Differentiation of THP1 cells was also observed morphologically. THP1 cells were treated with ISX or DMSO as a control, cytospin was performed followed by May-Grünwald Giemsa staining. Differentiated cells are indicated by arrows.

Notch activation has been implicated as a tumor suppressor in acute myeloid leukemia (AML) by arresting proliferation and inducing differentiation [14]. Thus, we hypothesized that ISX-mediated induction of the Notch signaling pathway could be used as a novel therapeutic tool. We treated several genetically different AML cell lines, such as THP1, Kasumi, and MOLM14 cells with ISX. In THP1 and MOLM14 cells, strong NOTCH3 induction was observed at both transcript and at protein levels while NOTCH2 and NOTCH1 were poorly or not influenced (Fig. 6A, B and Supplementary Figs. 11A and 12A, B). In line with this, we also observed an induction of Notch target genes *HES1, HES4* and *HEYL* upon treatment with ISX (Fig. 6C and Supplementary Fig. 12C). Importantly, ISX treatment leads to effects on cell growth as cells are arrested in the G1 phase (Fig. 6D and Supplementary Fig. 12D). However, ISX treatment does not influence cell viability (Fig. 6E and Supplementary Fig. 12E). When looking at differentiation markers, ISX treatment results in a strong upregulation of CD11b and CD86 (Fig. 6F and Supplementary Fig. 12F), suggesting that this treatment leads to terminal differentiation of AML cells. This is also evident morphologically when staining cells with May-Grünwald-Giemsa stain (Fig. 6G and Supplementary Fig. 12G). Of note, induction of differentiation marker-encoding genes is observed upon ISX treatment and this can be reverted by cotreatment with an EP300 inhibitor (EP300i; Supplementary Fig. 11B) as EP300 is a very well-known component of the NOTCH, coactivator complex [28]. Similar effects were also observed in Kasumi cells upon treatment with ISX (Supplementary Fig. 13): cell cycle arrest in G1 is observed whereas viability and/or apoptosis remains unaffected (Supplementary Fig. 13A, B). In Kasumi cells, ISX-treatment also results in strong induction of differentiation as also seen in staining of cytospin samples (Supplementary Fig. 13C, D). Altogether, these data suggest that treatment of ISX induced terminal differentiation of AML cells, resembling expression of activated NOTCH [14].

## Discussion

Here, we identify the small molecule compound ISX as a robust Notch activator and show that ISX activates an alternative NOTCH3 transcript leading to expression of an active NOTCH3 protein. We show that ISX induction is working in several different settings, including an in vivo system in zebrafish and differentiation in AML. This is further supported in studies by other investigators using ISX for ex vivo applications in diverse differentiation assays [20, 21, 29]. Since Notch activation is used for ex vivo T-cell differentiation [30], and this has also been shown in the human system [31], it remains to be seen whether ISX can contribute to increase the percentage of ex vivo generated T- cells, given that our results indicate that ISX is a potent and specific inducer of NOTCH3 signaling. A human congenital disease, CADASIL, associated with vascular dementia has been linked to the *NOTCH3* gene [32]. In addition, recently it was shown that vascular ageing in the brain has been associated with a decline of NOTCH3 expression [33]. Thus, it will be interesting to test whether ISX treatment is able to counteract such effects.

Mechanistically, our data support the notion that ISX treatment leads to a rise of cytosolic Ca^2+^ via mitochondrial release. Our data suggest that ISX binds directly to complex I of the respiratory chain, thereby inhibiting it, which in turn leads to Ca^2+^ release and activation of Ca^2+^dependent kinases and NFAT transcription factors, leading to activation of the cryptic promoter within the *NOTCH3* gene locus (Supplementary Fig. S14).

Our data is in line with the notion that ISX-mediated induction of a Notch response takes a few hours. Thus, we assume that de novo transcription and translation are necessary for full Notch activation and that NOTCH3 activation is required for full activation of the Notch pathway. Our gene expression data after ISX induction indicates also an upregulation of the Notch ligand JAG2 (see Supplementary Table 1). Thus, it is possible that initial ligand-independent NOTCH3 activation is followed by subsequent interaction of JAG2/NOTCH1 interaction and hence a more robust upregulation of Notch target genes.

The observation that the ISX-mediated induction of *HEY1* and *HEY2* is not sensitive to the GSI treatment, while *HES4* induction is partially reduced by GSI treatment, suggests that ISX can induce different transcriptional programs. In line with this, genetic depletion of NOTCH3 has no significant effect on the ISX-mediated induction of *HES4* in contrast to the induction of *HEY1* and *HEY2*. One possible explanation is that ISX-mediated induction of HES4 is under the control of other NOTCH receptors and/or other signaling pathways that are also influenced by the ISX treatment.

In regard to therapeutic relevance, our study suggests that ISX can be used in settings where Notch is known to play a tumor suppressive role; for example, in AML or head- and-neck squamous cell carcinoma (HNSCC). Such differentiation-inducing approaches of ISX might also be applicable to certain forms of lung cancer, since expression of Notch pathway genes is associated with improved response to immune checkpoint blockade in small cell lung cancer patients [34]. Thus, ISX might become a potent compound to induce antigen presentation in cancer cells to improve efficacy of T-cell based therapies.

Altogether, this is, to our best knowledge, the first description of a pharmacological Notch-inducer, which is not only useful for the better understanding of Notch signaling, but also provides mechanistic insights into how this potent compound functions in ex vivo differentiation assays, paving the way for therapeutic applications.

## Materials and methods

### Constructs

The NOTCH-dependent reporter construct 12xCSL refers to pGA981/6 and was described previously [28]. Human *NOTCH3* specific cDNAs (aa 1554-2321 and aa 1663- 2321) were commercially synthesized (BioCat, Heidelberg) as EcoRI/XhoI fragments and inserted into the corresponding sites of pcDNA3 (Invitrogen), pcDNA3-Flag-1 [28], and pcDNA3-GFPoStp [35]. The RBPJ expression construct pcDNA3.1-Flag-mRBPJ WT was described previously [36].

## Supplementary information

Supplementary material

Table S1

Table S2

Table S3

Table S4

source files_Western_blots

## Data Availability

All data generated or analyzed during this study are included in this published article and its supplementary information files. RNA-Seq, ATAC-Seq, and ChIP-Seq data generated in this study are available at GEO under accession number GSE288501.
