# Supplementary material for: Small molecule-mediated activation of Notch signal transduction
Source: Cell Death Dis. 2026 Jul 2;17(1):653. doi: 10.1038/s41419-026-09044-x (PMC13396519; doi:10.1038/s41419-026-09044-x)
Supplement: Supplementary file 1 — Supplementary material [file 41419_2026_9044_MOESM1_ESM.pdf]

Small molecule-mediated activation of Notch signal transduction

Subhamita Dey[1*], Benedetto Daniele Giaimo[1*], Iva Katharina Zöllner[2], Hina Zarrin[2], Qingwen Yang[2], Jasmin Ballout[3], Martin Diener[3], Tobias Friedrich[1,4], Francesca Ferrante[1], Jan Dreute[1], M. Lienhard Schmitz[1], Astrid Weiss[5], Pierfrancesco Polo[6], Matthias Lauth[6], Bernd Gahr[7], Steffen Just[7], Marek Bartkuhn[4], Franz Oswald[2] and Tilman Borggrefe[1#]

[1]Institute of Biochemistry, Justus-Liebig-University Giessen, Germany

[2]Department of Internal Medicine I, Center for Internal Medicine, University Medical Center Ulm, Germany

[3]Institute of Veterinary Physiology and Biochemistry, Justus-Liebig University, Giessen, Germany

[4]Biomedical Informatics and Systems Medicine, Justus-Liebig University, Giessen, Germany

[5] Excellence Cluster Cardio-Pulmonary Institute (CPI), Giessen, Germany and Department of Internal Medicine II, Universities of Giessen and Marburg Lung Center, University Hospital Giessen, Justus Liebig University, Member of the German Center for Lung Research (DZL), Giessen, Germany.

[6]Center for Tumor and Immune Biology, Clinics for Gastroenterology, Endocrinology and Metabolism, Philipps University Marburg, Germany

[7]Molecular Cardiology, Department of Internal Medicine II, Ulm University, Germany

* = these authors contributed equally to this work

# = To whom the correspondence should be addressed:

Electronic address: tilman.borggrefe@biochemie.med.uni-giessen.de

SUPPLEMENTARY MATERIALS AND METHODS

Zebrafish strains, RNA microinjections and treatment procedures

All procedures and experiments in this study were carried out after appropriate institutional approval [Tierforschungszentrum (TFZ) der Universität Ulm, No. z.183], which conforms to the EU Directive 2010/63/EU. Care and breeding of zebrafish (*Danio rerio*) were carried out as previously described(1, 2). The strain Tg[12xRBP:EGFP] was established as described before(3). Briefly, vector pDEST-Tol2-12xRBP:EGFP and transposase were co-injected in TüAB embryos. Mosaic fish were raised and two rounds of outcrosses with TüAB were performed from a single positive founder to establish a stable line. For Notch expression analysis, embryos were treated with 0.003% 1-phenyl-2-thiourea (w/v) to prevent melanization and with 0.5 µM to 10 µM ISX. GSI was used as Notch inhibitor, whereas DMSO was used as a solvent control. Embryos were treated for 24 hpf (hours post fertilization) to 72 hpf and imaged in an Acquifer Imaging machine. Pictures were taken on an Olympus stereo microscope SZX16. For RNA extraction and following RT-qPCR experiments, per replicate, 25 embryos of the wildtype strain TüAB were treated with either 5 µM ISX, GSI or DMSO from 24 hpf until 48 hpf.

Zebrafish RT-qPCR

Each biological replicate consisted of 25 pooled embryos. RNA extraction was carried out using the RNeasy Mini Kit (Qiagen, Düsseldorf, Germany) according to the manufacturer's instructions. Total RNA (1 000 ng) was reverse transcribed to produce cDNA using Superscript III reverse transcriptase (Life Technologies, Carlsbad, CA, USA). Quantitative real-time PCR was carried out according to the standard protocols using SYBR Green (Roche, Basel, Switzerland) on a Roche Light Cycler 480 II. Two

house-keeping genes, *rpl13* and *slc25a5*, were used for normalization. Primer sequences are indicated in **Supplementary Table 8**.

Cell culture, treatments and transfection

Cell lines MOLM14 (acute myeloid leukemia, DSMZ, ACC 777) and THP1 (acute monocytic leukemia, DSMZ, ACC 16) were grown in Roswell Park Memorial Institute (RPMI-1640, Gibco 61870-010) medium 1640 (1x) with addition of 10% fetal calf serum [FCS, (#S0115, Biochrom)] and 1% penicillin/streptomycin (Thermo). Kasumi cells (acute myeloid leukemia, DSMZ, ACC 220) were grown in the same manner but with 20% FCS. Cell lines HeLa (ATCC, CCL 2) and HEK293 (ATCC, CRL 1573) were grown in Dulbeccos Modified Eagle Medium (DMEM, Sigma-Aldrich), supplemented with 10% FCS and 1% penicillin/streptomycin. Human non-small cell lung cancer H1299 cell line was grown in RPMI-1640 (Gibco 61870-010) supplemented with 10% FCS (Pan Biotech) and penicillin/streptomycin (Gibco). H69 cell line was grown in RPMI-1640 (Gibco 61870-010) supplemented with 10% FCS (Pan Biotech), penicillin/streptomycin (Gibco) and 1.25 μg/ml Amphotericin B (Sigma A2942-100ML). Human primary pulmonary artery smooth muscles cells (hPASMCs) were a generous gift of Vanessa Nebel (University of Giessen). hPASMCs were grown in Smooth Muscle Cell Growth Medium 2 (Promocell C-22062) supplemented with penicillin/streptomycin (Gibco). Cells were grown at 37°C in presence of 5% CO_2.

H1299 cells were treated with 20 μM ISX (Cayman Chemical 16165), 23.1 μM γ-secretase inhibitor DAPT (Alexis ALX-270-416-M025) or DMSO as a control for specific time points as indicated in the respective figures. THP1 and MOLM14 cells were treated with 20 μM or 10 μM ISX or DMSO as a control for 24 or 48 hours. Kasumi cells were treated with 10 μM ISX or DMSO as a control for 24 or 48 hours. hPASMCs were treated with 20 μM ISX or DMSO as a control for 24 or 48 hours.

Trypsin-mediated cell dissociation of cell monolayer leads to transient activation of NOTCH1 and upregulation of Notch response genes. Hence, trypsin was used for routine sub-culturing of adherent H1299 cells but not for the collection of treated cells for accurate interpretation of results(4).

For overexpression of activated NOTCH3 (NICD3), H1299 cells were transiently transfected using linear polyethylenimine (PEI, Polysciences). Briefly, 1×10^6 H1299 cells were seeded on 10 cm plate in 10 ml of medium and incubated for 16-24 hours. 20 µl of PEI were diluted in 309 µl of PBS, 20 µg of DNA were mixed with 325 µl of PBS, and the two solutions were combined together. After 30 min of incubation at room temperature, DNA solution was added dropwise to the cells. The medium was replaced with fresh one 6 hours after incubation at 37°C in presence of 5% CO_2.

Generation of CRISPR/Cas9 depleted H1299 cells

Genomic depletion of RBPJ and NOTCH3 was achieved by CRISPR/Cas9-based technique. The guide RNAs for targeting RBPJ was designed using Zhang Lab CRISPR Design Tool(5). The desired 5' overhangs were added and the oligos were phosphorylated, annealed and ligated into the dephosphorylated vector backbone pSpCas9(BB)-2A-Puro (PX459) V2.0 digested with BbsI. The RBPJ-depleted clones were generated using the combination of hRBPJ guide RNAs gRNA #1 and gRNA #2. The NOTCH3-depleted clones were generated using the combination of hNOTCH3 guide RNAs gRNA #1 and gRNA #2. H1299 cells were transfected with 10 µg of each px459 v2.0 plasmid together using Lipofectamine 2000 Transfection Reagent (Invitrogen) according to manufacturer's instructions. After at least 6 hours of incubation at 37°C, the medium was replaced with fresh one, and 48 hours post-transfection, cells were selected with puromycin. After establishing single-cell clones, the individual clones were expanded and further analyzed to purify the genomic DNA

(gDNA) that was further analyzed by PCR. gDNA was purified using standard procedure. Briefly, after washing twice in PBS, cells were resuspended in gDNA extraction buffer [10 mM Tris–HCl pH 7.5, 10 mM ethylenediaminetetraacetic acid (EDTA) pH 8.0, 10 mM NaCl, 0.5% N-Lauroylsarcosine sodium salt, 1 mg/ml proteinase K (Roche 03115852001)] and incubated overnight at 37°C. gDNA was precipitated with 100% ethanol in presence of 50 mM NaCl, washed with 70% ethanol, dried and resuspended in Tris-HCl/EDTA (TE) buffer pH 8.0. gDNA was used to validate genome editing by PCR screening with the PCR primers listed in **Supplementary Table 8** and the PCR products were analyzed by agarose gel electrophoresis. The depletion was further validated by Western blotting.

Cloning

All oligonucleotides used to generate the constructs expressing CRISPR/Cas9 guide RNAs (gRNAs) are listed in **Supplementary Table 8**. The pSpCas9(BB)-2A-Puro (PX459) V2.0 (Addgene), pcDNA 3.1 Flag2 were commercially acquired. The cDNA for murine NFAT2 (synonyms: NFATC1; NFATC; Accession: NP_001157581) was PCR amplified from a cDNA library of mouse peripheral blood mononuclear cells (mPBMCs) using the primers indicated in **Supplementary Table 8**. After digestion with Acc65I and XhoI the PCR product was inserted into the corresponding sites of pcDNA3-EGFP-ATG, resulting in pcDNA3-mNFAT2-EGFP. For the human NOTCH3 Exon 24 luciferase reporter constructs, Exon 24 (ENSE00002724304 in transcript ENST00000263388.7, 566bp, location: Chr 19:15.178.090; Chr 19: 15.177.525) flanked by 65 bp of intron 23-24 and 632 bp of intron 24-25 was synthesized commercially (Biocat) and inserted into the SmaI site of pGL3-Basic and pGL3-Enhancer (Promega) in both orientations, resulting in pGLB-N3E24(+), pGLE-N3E24(+), (sense orientation), pGLB-N3E24(-) and pGLE-N3E24(-), (antisense orientation), (see Supplemetary Fig. S10C).

Respiratory chain complex activity assays

Activity assays were performed according to Spinazzi et al.(6). Briefly, Complex I activity was quantified by measuring NADH oxidation (ε_{340nm} = 6.2 $mM^{-1*}cm^{-1}$). Sucrose gradient purified bovine heart mitochondria (10 µg) were resuspended in 25 mM potassium-phosphate buffer at pH 7.5 with 2 mg/ml fatty acid-free BSA and 20 µM oxidized cytochrome *c*. The NADH-site assay was performed in 25 mM Tris-HCl, pH 8.5 with 10 µM Rotenone, 2 µM Antimycin A and 1.2 mM Ferricyanide. The Q-site assay was performed in 25 mM potassium-phosphate buffer pH 7.5 and supplemented with 3 mg/ml fatty acid-free BSA and 70 µM decylubiquinone. Reactions were started by adding 120 µM NADH. Rotenone (10 µM)-sensitive activity was subtracted to determine complex I specific activity. Complex II activity was quantified by measuring 2,6-dichlorophenolindophenol (DCPIP) reduction (ε_{600nm} = 19.1 $mM^{-1*}cm^{-1}$). Bovine heart mitochondria (5 µg) were resuspended in 25 mM potassium-phosphate buffer pH 7.5 with 1 mg/ml fatty acid-free BSA, 10 µM Rotenone, 2 µM Antimycin A, 20 mM succinic acid and 80 µM DCPIP. 10 minutes prior to the assay start, succinic acid was added to the samples. The assay was started by adding 70 µM decylubiquinone. Malonate (10 mM)-sensitive activity was subtracted to determine complex II specific activity. Complex III activity was quantified by measuring cytochrome *c* reduction (ε_{550nm} = 18.5 $mM^{-1*}cm^{-1}$). Bovine heart mitochondria (5 µg) were resuspended in in 25 mM potassium-phosphate buffer pH 7.5 with 500 µM KCN, 100 µM EDTA, 2 µM Rotenone and 100 µM $DBQH_2$. The assay was started by adding 75 µM oxidized cytochrome *c*. Antimycin A (2 µM)-sensitive activity was subtracted to determine complex III specific activity. Complex IV activity was quantified by measuring cytochrome *c* oxidation (ε_{550nm} = 18.5 $mM^{-1*}cm^{-1}$). Bovine heart mitochondria (5 µg) were resuspended in 25 mM potassium-phosphate buffer pH 7.0 with 2 µM Antimycin

A. The assay was started by adding 75 µM reduced cytochrome *c*. KCN A (500 µM)-sensitive activity was subtracted to determine complex IV specific activity.

PAM station

For kinome profiling all experiments were performed in technical triplicates, where H1299 cells were incubated with the vehicle control (DMSO) or ISX for 30 minutes. Next, cells were washed with PBS and scraped down in M-PER lysis buffer including protease and phosphatase inhibitors (Thermo Fisher Scientific, Waltham, MA, USA). After homogenization by passing through a syringe, the lysates were incubated for 1 hour at 4°C on a rotating wheel before centrifugation at 13 000 g for 20 minutes. The supernatant containing the protein extract was divided in 12 µl aliquots and stored at -80°C. Subsequent peptide-based kinase activity profiling was carried out by the PamStation 12 platform (PamGene International, s-Hertogenbosch, Netherlands) using the phospho-tyrosine kinase (PTK) and serine/threonine kinase (STK) chips chips as previously described(7). Briefly, 10 µg protein lysate were applied on PTK chips while only 2 µg were needed for STK chips. The corresponding reaction buffer including ATP was prepared as recommended by the manufacturer (PamGene). Active kinases in the individual cell lysates phosphorylate their distinct sets of peptides on the chip and phosphorylated peptides are recognized by phospho-specific FITC-labeled antibodies. Antibody binding to the target sequence is detected by fluorescence and documented by a CCD camera. Data acquisition, i.e. peptide substrate phosphorylation, was performed using the Evolve3 software (version 3.1.0.5 PamGene). The prediction of differentially activated, upstream kinases in ISX-treated cells versus DMSO control samples was based on the phosphorylation of distinct substrates (*i.e.* peptides) immobilized on the chip surface. Software-based image analysis by the Bionavigator (BN6 version 6.3.67.0, PamGene) integrated the various

signals per array over time into one single value for each peptide for each sample. Numerical raw data were log2-transformed and normalized (*e.g.* by centering, $x_{center} = x - mean[x]$) for the three technical replicates. The analysis of corresponding upstream kinases responsible for selective peptide phosphorylation was performed using Bionavigator software, which allows for kinase identification using post-translational modification (PTM) databases such as human protein reference database (HPRD), PhosphoSitePlus, or databases with in silico predictions such as PhosphoNET, which contains predictions for a large number of kinases.

Ca^{2+} imaging

One thousand H1299 cells were transferred to each glass coverslip in a four well chamber with RPMI medium for 24 hours [37 °C, 5 % CO_2 (v/v)]. Prior to the beginning of the Ca^{2+} imaging experiments, the medium was removed and cells were incubated in Tyrode solution containing 6 µM of the Ca^{2+}-sensitive dye fura-2 acetoxymethylester (fura-2-AM, Thermo Fisher Scientific) and an equal volume of pluronic F-127 [20 % (v/v) stock solution in DMSO; Thermo Fisher Scientific] at room temperature for approximately 60-90 minutes. After washing with 500 µl Tyrode solution, cells were transferred into the imaging chamber and Ca^{2+} imaging experiments were started. Experiments were carried out at room temperature either with Tyrode solution containing $CaCl_2$ or not (Ca^{2+}-free). Cells were alternately excited at 340 and 380 nm and emission > 440 nm was measured with an imaging analysis system (Till Photonics, Martinsried, Germany) combined with an epifluorescence setup and an inverted microscope (Olympus IX-50, Olympus, Hamburg, Germany). The fura-2 ratio (340/380 nm) of single H1299 cells (= regions of interest, ROI) was recorded reflecting changes in the cytosolic Ca^{2+} levels. Data were sampled at 0.2 Hz. After a stabilization phase of 3 minutes, cells were stimulated with ISX (20 µM), histamine (100 µM) or DMSO

followed by cyclopiazonic acid (CPA; 10 µM; inhibitor of sarcoplasmatic/endoplasmatic reticulum Ca^{2+}-ATPase / SERCA) as viability control. The increase in fura-2 ratio was calculated as Δfura-2 ratio compared to baseline parameters before drug administration. A response to the respective drug was accepted, when two conditions were fulfilled simultaneously: 1. The amplitude of the change exceeded the 4-fold standard deviation of the scattering in the fura-2 ratio during the control period just prior to addition of the drug; 2. The amplitude of the change in the fura-2 ratio exceeded an absolute value of 0.1.

RNA extraction, RT-qPCR and RNA-Seq

Total RNA was purified using Trizol reagent (Ambion) accordingly to the manufacturer's instructions. 1 µg of RNA was reverse transcribed into cDNA using random hexamers and M-MuLV reverse transcriptase (NEB). RT-qPCRs were assembled with Absolute QPCR ROX Mix (Thermo Scientific) or SybrGreen (Thermo Fisher Scientific), gene-specific oligonucleotides and double-dye probes (*see* Supplementary Table 8) and analyzed using a QuantStudio 3 Real Time PCR system (Applied Biosystem). Data were normalized to the housekeeping gene Glyceraldehyde-3-phosphate dehydrogenase (*GAPDH*).

For RNA-Seq purposes, total RNA was purified using the RNeasy Mini Kit (Qiagen 74104), the QIAshredder (Qiagen #79654) and the DNase I (Qiagen 79254) accordingly to manufacturer´s instructions. Samples were sequenced on a NovaSeq6000 device at Novogene UK.

RNA-Seq analysis

Raw sequencing reads were adapter and quality trimmed using *Trim Galore (https://github.com/FelixKrueger/TrimGalore)*. Trimmed reads were aligned against the

human reference genome (hg19) using HISAT2 (8) and stored as BAM files. Genomic alignment's ‘summarizeOverlaps’ function using the filtered BAM files and the hg19 GTF file (downloaded from Illumina's iGenomes) was used to generate read counts per gene. Read counts per gene were normalized and used to calculate differentially expressed genes using DESeq2 (9). Gene over-representation analysis and Gene Set Enrichment Analysis was performed using the clusterProfiler (10) R package.

ChIP-Seq and ATAC-Seq

For ChIP-Seq experiments, cells were crosslinked in 1% FMA for 10 min at room temperature. Only in the case of the RBPJ ChIP-Seq, cells were washed twice with PBS, fixed for 1 hour at room temperature in 10 mM dimethyladipimate (DMA, Thermo Scientific 20660) dissolved in PBS and, after washing once in PBS crosslinked in 1% FMA for 30 min at room temperature. The FMA reaction was blocked by adding 1/8 volume of 1 M glycine pH 7.5 and incubating for 5 min at room temperature. ChIP was essentially done as previously described(11) using an anti-RBPJ antibody (Cell Signaling Technology, 5313S), an anti- H3K27ac (Diagenode, pAb-174-050), an anti-H3K4me1 (Abcam, ab8895) or an anti-H3K4me3 (Diagenode, pAb-003-050). Chromatin from *Drosophila melanogaster* Schneider cells was used for spike-in purposes in presence of 2 µg of anti-His2Av antibody (Active Motif 61686) for each immunoprecipitation.

Libraries were prepared using the Diagenode MicroPlex Library Preparation kit v3 (Diagenode C05010001) following the manufacturer’s instructions with few modifications. Libraries were purified with Agencourt AMPure XP Beads (Beckman Coulter, #A63881), quantified and analyzed on an Agilent Tapestation device. Finally, sequencing was done on a NovaSeq6000 device at Novogene UK.

ATAC-Seq was done using the ATAC-Seq kit (Active Motif 53150) accordingly to manufacturer´s instructions and samples were sequenced on a NovaSeq6000 device at Novogene UK.

ChIP-Seq and ATAC-Seq analysis

Raw sequencing reads for ChIP-Seq and ATAC-Seq were adapter and quality trimmed using *Trim Galore*. Trimmed reads were aligned against the human reference genome (hg19) using HISAT2 with "--no-spliced-alignment" parameter and stored as BAM files. BAM files were filtered for PCR duplicates using Picard tools (https://broadinstitute.github.io/picard/). Normalized coverage tracks were generated using deepTools' *bamCoverage* function (12). Peak calling was performed using MACS2 (13) with the corresponding input. Peaks were annotated to their corresponding gene using ChIPseeker (14). Heat map of RBPJ binding was generated using deepTools' *computeMatrix* and *plotHeatmap* functions. Snapshots were generated using GVIZ (15). Motif identification was performed using MEME-suite (16).

Giemsa staining

After pelleting (1 000 rpm, 5 min, 4°C) cells were resuspended at $0.5x10^6$/ml in ice-cold PBS. Cells (50×10^4) were then centrifuged to a microscope glass slide with a Cytospin 4 Cytocentrifuge (Thermo) using Epredia™ EZ Double Cytofunnel™ (Thermo) at 1 000 rpm for 10 min, followed by fixation with 100% methanol (100%). Fixed cells were subsequently stained by May-Grünwald solution (#63590, Merck), Giemsa working solution (1:20 dilution of stock, #T862.1, Carl Roth) and analyzed by microcopy.

FACS analysis

Cells (app. 2x10^6) were pelleted, and incubated with Human BD Fc Block™ (20 min, RT) and subsequently washed with washing buffer [2% FCS, Biochrom), 0.02% Sodium azide (Merck #6688) in phosphate buffered saline (PBS)]. Cells were then incubated with isotype control for compensation (1:10) and corresponding antibodies (*see* **Supplementary Table 9**) for staining (20 min on ice, protected from light from this step on). After fixation (fixation buffer, 2% PFA in PBS, 10 min). 20.000 single cell events were processed and recorded by a BD FACSymphonyTM A1 cell Analyzer (BD Biosciences). Analysis was performed by FlowJo (BD Biosciences, San José, USA). Isotype controls and antibodies used are shown in **Supplementary Table 10**.

Cell cycle analysis

Cells were fixed in 70% ice-cold ethanol and stored at -20° until analysis. After washing, cells were incubated in 400 µl of staining solution [1µg/ml DAPI (4′,6-Diamidino-2-Phenylindol), 0.1% triton-X-100 in PBS] for 5 min at 37°C. The stained cell suspension was loaded on a NC-slide A8 (Chemometec). Measurement was performed using the Nucleocounter NC-3000™ device (Chemometec) according to the manufaturers´ instructions. Analysis was performed using the Nucleoview software (Chemometec).

Viability assay

Cells were harvested and loaded to the Via-Cassette™ prefilled with AO (Acridinium orange) and DAPI. Measurements were performed using the Nucleocounter NC-3000™ device (Chemometec) according to the manufaturers´ instructions. Analysis was performed using the Nucleoview software.

Luciferase assays

HeLa$^{RBPJ-KO}$ cells were seeded in 48 well plates at a density of 2 × 10^5 cells. Transfection was performed with Lipofectamine 2000 reagent as described above using 500 ng of reporter plasmid alone or together with 100 ng of RBPJ expression plasmid (see above) and various amount of NOTCH3 expression plasmids (given in the corresponding figure legend). After 24 hours, luciferase activity was determined from eight independent experiments. For analysis of NFAT2 dependent transactivation of the Notch3 specific exon 24 reporter constructs (see figure S10C), HeLa cells (22.000) were seeded in 48 well plates. 24 hours later cells were transfected with 250 ng of reporter constructs alone or together with 50 ng of NFAT2 expression constructs. Twenty-two hours after transfection, cells were treated with PMA (80 nM) and Ionomycin (2 µM) or DMSO as a control. Six hours after treatment, cells were harvested and luciferase activity was determined from 4 independent experiments. All measurements were performed with 20 µl of cleared lysate with a Centro LB 960 luminometer (Berthold) by using the luciferase assay system from Promega.

Treatment of HEK293 cells with Ionomycin and ISX

HEK293 cells were plated on double chamber coverslips (Nunc Labtec) with a density of 80.000 per chamber.). Cells were transfected with 200 ng of the NFAT2-EGFP construct with the Lipofectamine 2000 transfection reagent (Invitrogen) according to the manufacturer's instructions. Twentyfour hours after transfection cells were treated with 2 µM Ionomycin (Merck) or 10 µM ISX or DMSO as a control. Images were taken from living cells after 6 hours as described in the "fluorescence microscopy" section.

Fluorescence microscopy

HeLa cells were plated (1x10^5 cells/cm^2) on chamber coverslips (Nunc Labtek). Cells were transfected with 150 ng of *NOTCH3* specific expression plasmids (indicated in

the respective figure legend) after 18 hours. 24 h after transfection, cells were fixed (4% paraformaldehyde, Merck), stained with DAPI and imaged using an IX71 fluorescence microscope (Olympus) equipped with a digital camera (C4742, Hamamatsu) and a HBO103W/2 mercury lamp, (Osram). The following filter sets were used: GFP detection, ex: HQ470/40, em: HQ525/50, DAPI detection, ex: D360/50, em: D460/50.

Preparation of nuclear extracts, whole cell extract (WCE) and Western Blotting

Whole cell extracts were prepared as follows: cells were washed twice with PBS and resuspended in lysis buffer [20 mM Tris-HCl pH 8.0, 150 mM NaCl, 1% NP-40 (v/v), 10% glycerol (v/v), 0.5 mM Na_3VO_4, 10 mM NaF, 1 mM PMSF, 1x protease inhibitor cocktail mix] and incubated on ice for 15 min. The suspension was centrifuged at 13,200 rpm for 15 min at 4°C. The clear supernatant was transferred to a fresh tube and the amount of protein was quantified by Bradford protein assay solution (Sigma-Aldrich). Extracts were boiled in presence of sodium dodecyl sulfate (SDS) loading buffer and analyzed by Western blotting.

Nuclear extracts (NEs) were prepared as follows: cells were washed twice with PBS and resuspended in sucrose buffer [320 mM sucrose, 3 mM $CaCl_2$, 2 mM MgAc, 0.1 mM EDTA, 10 mM Tris-HCl pH 8.0, 1 mM dithiothreitol (DTT), 0.5 mM PMSF, 0.5% NP-40, 1x Protease inhibitor], incubated 20 min on ice and vortexed in 20 seconds intervals. Cell lysis was ensured by staining with Trypan Blue followed by microscopic examination. Lysates were centrifuged at 4 000 rpm at 4°C for 5 min. The cell pellet was washed with pre-cooled PBS and resuspended in high salt buffer (20 mM HEPES pH 7.9, 25% glycerol, 1.5 mM $MgCl_2$, 800 mM KCl, 0.2 mM EDTA, 1% NP-40, 0.5 mM PMSF, 1x Protease inhibitor mix, 0.5 mM DTT). Lysates were incubated for 20 min on ice in the cold room followed by centrifugation for 15 min at 14 000 rpm at 4°C. The

supernatants representing the NEs were collected and protein concentration was measured by Bradford protein assay solution (Sigma-Aldrich). In the case of THP1 and MOLM14 cells, NEs were prepared as follows: After washing the cells pellet with PBS, cell were lysed in hypotonic buffer (20mM Hepes pH 7.9, 20mM NaCl, 5mM $MgCl_2$, 10% glycerol, 0.2mM PMSF) by incubating 20 minutes on ice and centrifuging 10 minutes at 4000 rpm at 4°C. Pellet was washed in pre-cooled PBS and resuspended in Buffer C (20mM Hepes pH 7.9, 1mM $MgCl_2$, 300mM NaCl, 0.2% NP-40, 25% glycerol, 0.2mM PMSF, 1x Protease inhibitor mix, 0.3mM DTT) After 20 minutes incubation on ice, samples were centrifuged 14000 rpm at 4°C for 15 minutes. The supernatants representing the NEs were collected and protein concentration was measured by Bradford protein assay solution (Sigma-Aldrich). NEs were boiled in presence of SDS loading buffer and analyzed by Western blotting. For Western blotting, proteins were resolved in SDS polyacrylamide gels and transferred to a nitrocellulose membrane (Amersham) by wet blotting. Membranes were blocked with 5% milk / TBST [1x Tris-buffered-saline (TBS), 0.1% Tween 20] and incubated over night with desired primary antibodies against ERK 1/2 (1:1 000; Cell Signaling Technology 9102), GAPDH (1:2 500; abcam ab8245), H3 (1:1 000; abcam ab1791), NOTCH1 (1:1 000; Cell Signaling Technology 4147), NOTCH2 (Cell Signaling Technology 5732), NOTCH3 (1:1000; Cell Signaling Technology 5276), phospho-ERK 1/2 (p-ERK 1/2, 1:1 000; Cell Signaling Technology 4370), RBPJ (1:1 000; Cell Signaling Technology 5313), TBP (1:1 000, abcam ab818) diluted in 5% milk / TBST. Membranes were washed in TBST and incubated 1 hour at room temperature with secondary antibody diluted 1:5 000 in 5% milk/ TBST [anti-rabbit IgG HRP (Cell Signaling Technology 7074) or anti-mouse IgG HRP (Cell Signaling Technology 7076)]. Membranes were finally washed in TBST and incubated at room temperature

with ECL (solution. Chemiluminescence was detected with a Vilber Fusion FX7 system.

Expression of NOTCH3 variants in HEK293 cells

Transfection of HEK293 cells with Notch3 specific expression plasmids was performed using the Lipofectamine 2000 transfection reagent (Invitrogen, #11668019) according to the manufacturer's instructions. Cells were lysed 24 hours after transfection using 600 µl CHAPS lysis buffer containing protease inhibitors. After an incubation on ice for 1 h, we centrifuged the samples for 30 minutes at 14 000 rpm and 4 °C. The supernatant was collected and the protein concentration was determined with a Bradford protein assay (Biorad, # 5000006). Protein lysates (20 µg) 6x Laemmli buffer and applied it to a 10% SDS polyacrylamide gel. After gel electrophoresis, we blotted the proteins at RT on a PVDF membrane (Merck, Cat. # IPVH00010). Membranes were blocked for 1 hour at room temperature with 5 % BSA (Serva, Cat. No.:9048-46-8) dissolved in TBS with 0.1% Tween-20, prior to incubation with the primary antibody [Flag-M5, mouse monoclonal, Sigma, #F4042; anti-Notch3, D11B8, rabbit monoclonal, Cytiva, #5276). We used horseradish peroxidase (HRP) conjugated secondary antibodies against mouse (Cytiva, # NA931V) or rabbit (Cytiva, #NA934V, Cat. No.: 112-035-071) for detection with ECL solution (Cytiva, # RPN2209). The resulting chemiluminescence signal was documented with high performance chemiluminescence films (Cytiva, Cat. No.: 28906837).

Cell viability and cell cycle and morphological analyses

Cell cycle analysis of AML cells (THP1, Kasumi1 and MOLM14) were performed using fluorescence image cytometer NucleoCounter NC-3000 (Denmark). 2 million cells were fixed for 2 hours in 70% ethanol. Cells were stained with 1 µg/ml DAPI and

analyzed to measure by fluorescence DNA content. For analyses we used the NucleoView NC-3000 software, version 1.4. For morphological analysis cells were stained with May-Grünwald-Giemsa stains after cytospin. For microscopic images we used 400x magnification.

Data and statistical analysis

Results are represented as mean ± SD of the number of experiments (n) as indicated in the figure legend for each experiment. Statistical analyses are indicated in the respective figure legend and have been done with GraphPad Prism or with R.

Analysis of the Kozac sequence

The search for Kozac sequences within the NOTCH3 locus has been done using TIS Predictor, available at https://www.tispredictor.com/ (17).

SUPPLEMENTARY FIGURES

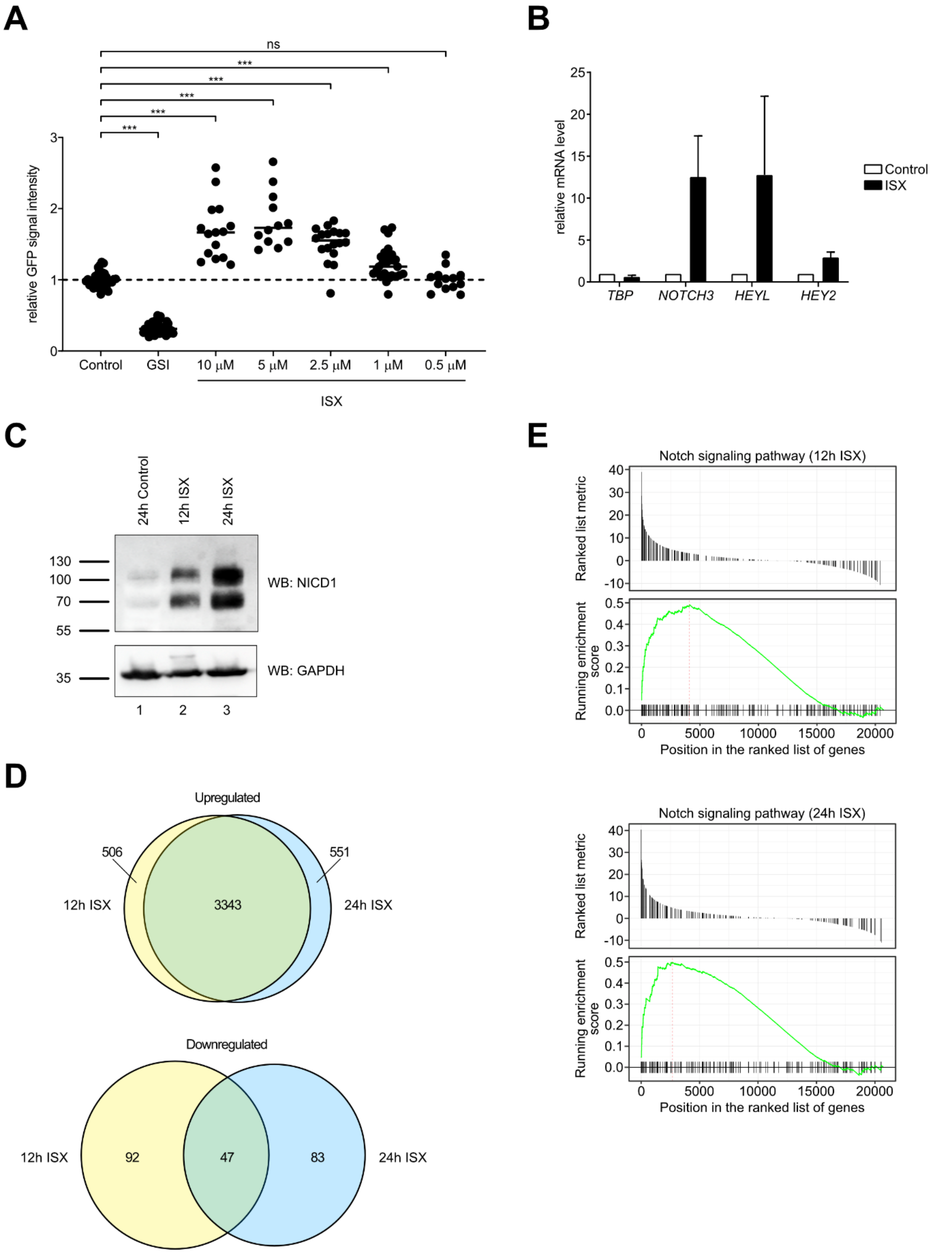

Supplementary Figure 1. ISX induces the expression of Notch in zebrafish and in lung cancer cells. (**A**) ISX-induced Notch reporter activation in zebrafish embryos is dose-

dependent. Tg[12xRBP:EGFP] embryos were treated for 48 hours with different doses of ISX: 10 μM and 5 μM of ISX result in a strong activation of the Notch reporter while a weaker activation is observed at 2.5 μM and 1 μM of ISX. Treatment with 0.5 μM ISX did not activate the Notch reporter (***P < 0.001, ns = not significant). (**B**-**E**) ISX treatment induces the Notch signaling pathway in human lung cancer cells. (**B**) ISX treatment leads to the upregulation of Notch target genes in H69 small cell lung carcinoma cells. H69 cells were treated with 20 μM ISX or DMSO as a control for 24 hours. Total RNA was purified, reverse transcribed and Notch target genes expression was analyzed by RT-qPCR using gene-specific primers for *TBP*, *NOTCH3*, *HEYL* and *HEY2*. Data were normalized to the housekeeping gene *GUSB* and represent the mean ± SD of two independent experiments. (**C**). ISX treatment leads to increased cleaved and active Notch intracellular domain 1 (NICD1) protein level. H1299 cells were treated for 12 and 24 hours with 20 μM ISX or DMSO as a control. Whole cell extracts (WCE) were analyzed by Western blotting versus NICD1 or GAPDH as a loading control. (**D**-**E**) H1299 cells were treated with 20 μM ISX or DMSO as a control for 12 or 24 hours and, after purification, the RNA was analyzed by deep sequencing. (**D**) Venn diagram showing the overlap for genes up- or downregulated upon 12 and 24 hours of ISX treatment in H1299 cells. (**E**) Gene Set Enrichment Analysis (GSEA) for genes deregulated upon 12 or 24 hours of ISX treatment in H1299 cells.

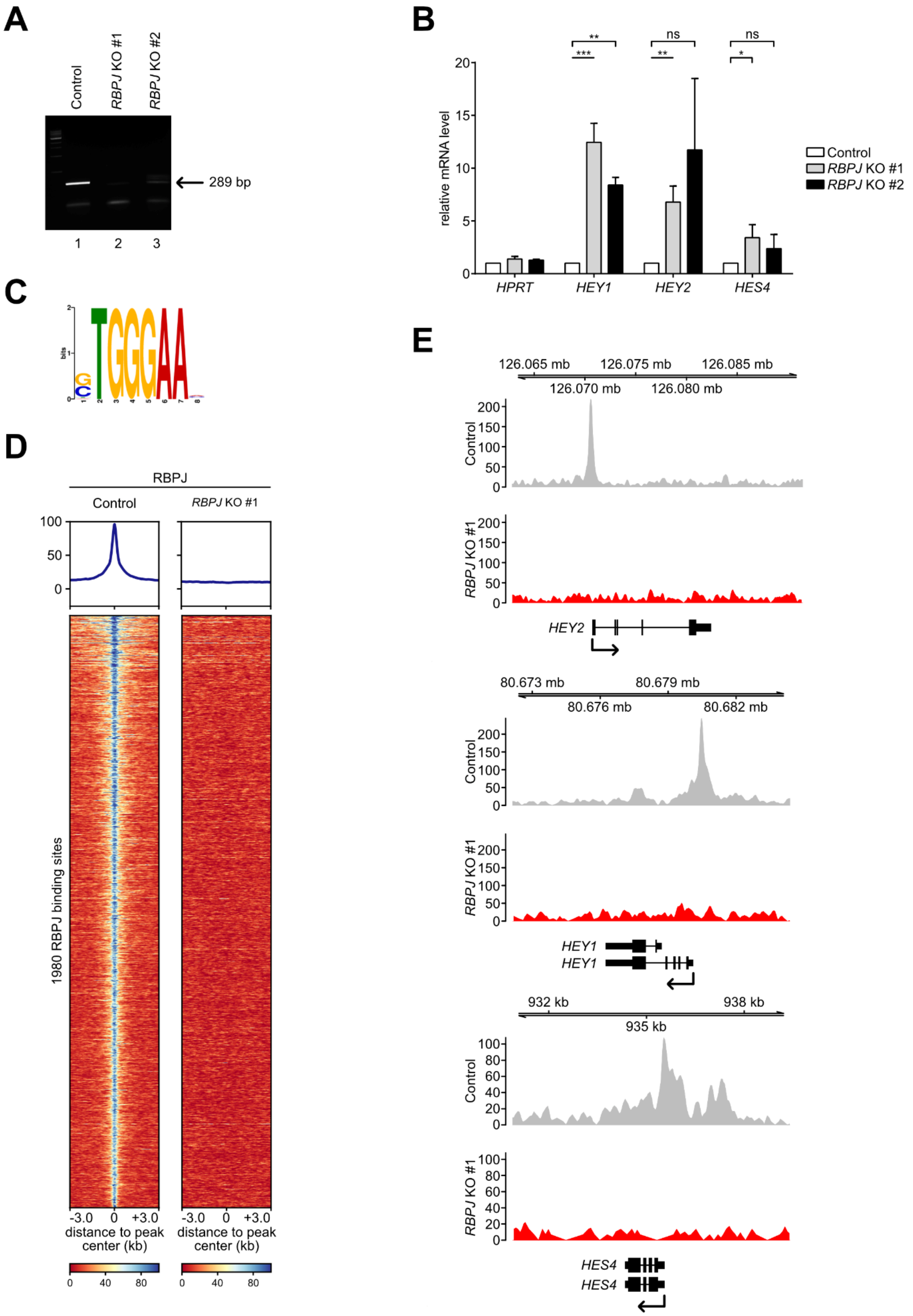
A
Control
RBPJ KO #1
RBPJ KO #2
289 bp
1 2 3
B
relative mRNA level
HPRT
HEY1
HEY2
HES4
Control
RBPJ KO #1
RBPJ KO #2
ns
C
D
RBPJ
Control
RBPJ KO #1
1980 RBPJ binding sites
distance to peak center (kb)
distance to peak center (kb)
E
126.065 mb
126.075 mb
126.085 mb
126.070 mb
126.080 mb
Control
RBPJ KO #1
HEY2
80.673 mb
80.679 mb
80.676 mb
80.682 mb
HEY1
HEY1
932 kb
938 kb
935 kb
HES4
HES4

Supplementary Figure 2. Identification of RBPJ/NOTCH target genes in H1299 cells. Depletion of RBPJ in H1299 cells was achieved making use of the CRISPR9/Cas9 technology. (**A**) Genomic DNA (gDNA) was purified and analyzed by PCR using primers specific for the *RBPJ* locus. (**B**) Total RNA was purified from RBPJ-depleted and control H1299 cells, the RNA was reverse transcribed and Notch target genes expression was analyzed by RT-qPCR using gene-specific primers for *HPRT*, *HEY1*, *HEY2* and *HES4*. Data were normalized to the housekeeping gene *GAPDH* and represent the mean ± SD of three independent experiments (**P* < 0.05, ***P* < 0.01, ****P* < 0.001, ns = not significant, unpaired Student's t-test). (**C**-**E**) Characterization of RBPJ occupancy in H1299 cells. RBPJ binding was analyzed in control or RBPJ-depleted H1299 cells by ChIP-Seq. (**C**) MEME analysis of the ChIP-Seq identified the canonical RBPJ binding motif. (**D**) Heatmap showing the RBPJ binding sites identified in H1299 cells. The signal identified in control cells is absent in RBPJ-depleted cells. (**E**) Representative snapshots showing the RBPJ binding at *HEY2*, *HEY1* and *HES4* loci.

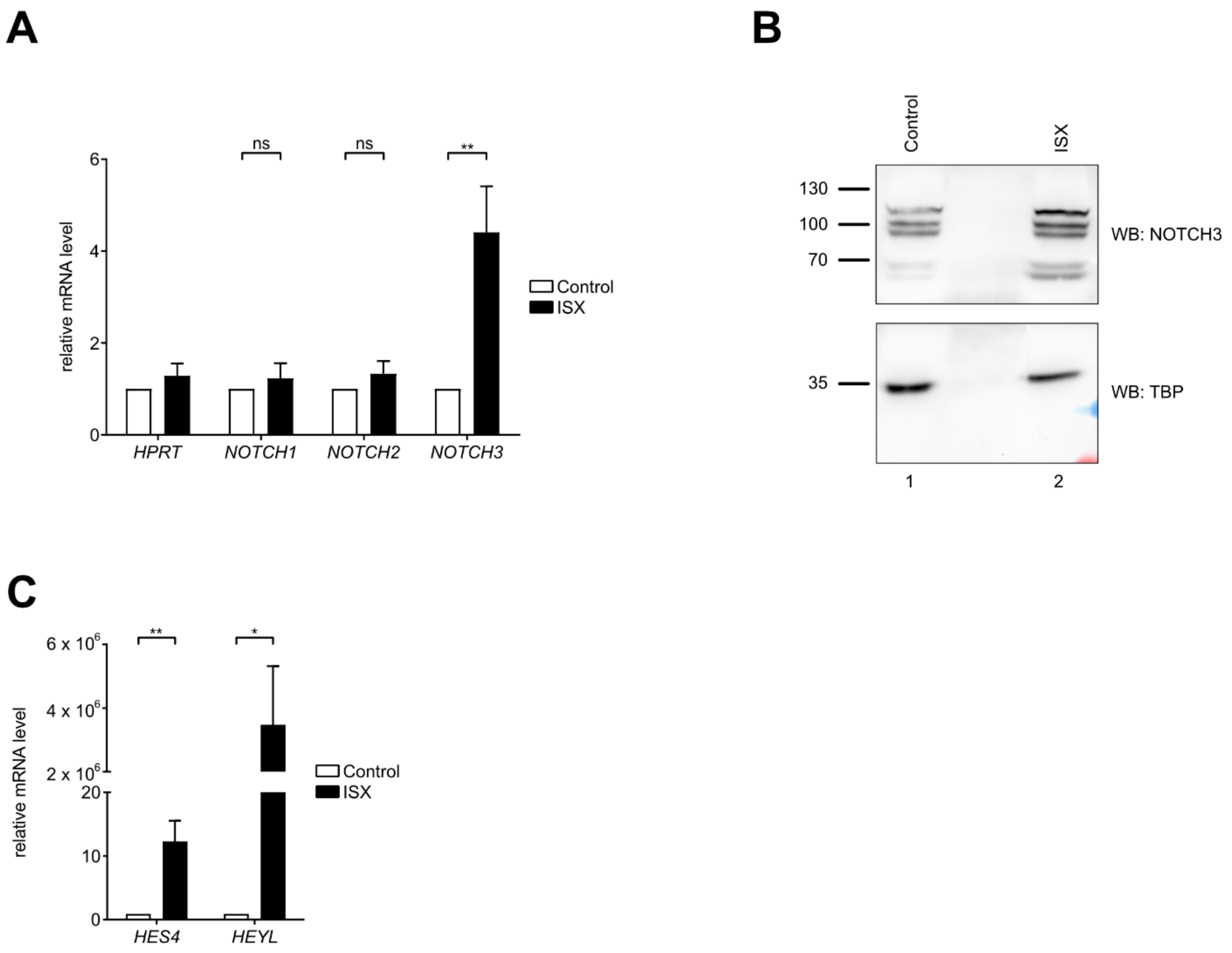

Supplementary Figure 3. ISX treatment induces the Notch signaling pathway in human primary pulmonary artery smooth muscles cells (hPASMCs). (**A-C**) Human primary pulmonary artery smooth muscles cells (hPASMCs) were treated with 20 µM ISX or DMSO as a control for 24 hours. (**A** and **C**) Total RNA was purified, reverse transcribed and analyzed by qPCR. (**A**) Expression of NOTCH receptors was analyzed by RT-qPCR using gene-specific primers for *HPRT*, *NOTCH1*, *NOTCH2* or *NOTCH3*. (***P* < 0.01, ns = not significant, unpaired Student's t-test). (**B**) ISX treatment leads to increased cleaved and active NOTCH3 protein level. hPASMCs were treated for 24 hours with 20 µM ISX or DMSO as a control. Whole cell extracts (WCE) were analyzed by Western blotting versus NOTCH3 or TBP as a loading control., (**C**) Notch target genes expression was analyzed by RT-qPCR using gene-specific primers for *HES4* and *HEYL*. Data were normalized to the housekeeping gene *GAPDH* and represent the mean ± SD of three experiments. (**P* < 0.05, ***P* < 0.01, unpaired Student's t-test).

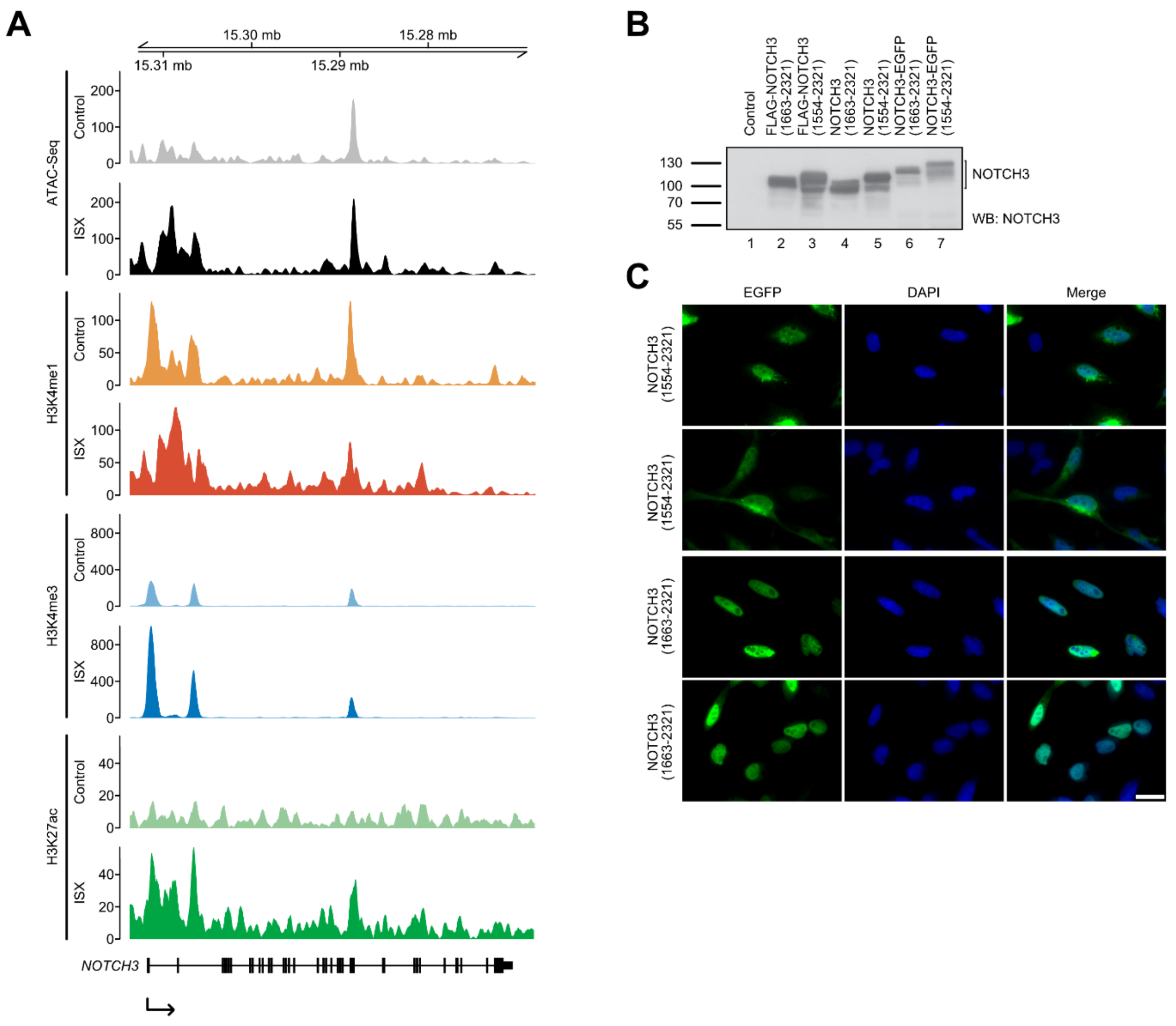

Supplementary Figure 4. Analysis of the chromatin configuration of the *NOTCH3* locus in response to ISX treatment and expression analysis of the NOTCH3 protein fragments. (**A**) H1299 cells were treated with 20 µM ISX or DMSO as a control for 12 hours and the chromatin configuration was analyzed via ATAC-Seq or ChIP-Seq versus H4K4me1, H3K4me3 or H3K27ac. Analysis of the *NOTCH3* locus indicates two strong peaks in ATAC-Seq signal at exon-1 and exon-23 within the which are enriched for H3K4me1, H3K4me3 and, upon treatment with ISX, with a strong enrichment in H3K27ac. (**B**) Western blotting for performed to analyze the protein expression of NOTCH3 C-terminal fragments either FLAG-tagged (lanes 2 to 5) or GFP-tagged (lanes 6 and 7). (**C**) Cellular localization of NOTCH3 C-terminal fragments: C-terminal GFP-tagged NOTCH3-fragments localize within the nucleus as they co-localize with the DAPI staining (blue). Images are shown in duplicates.

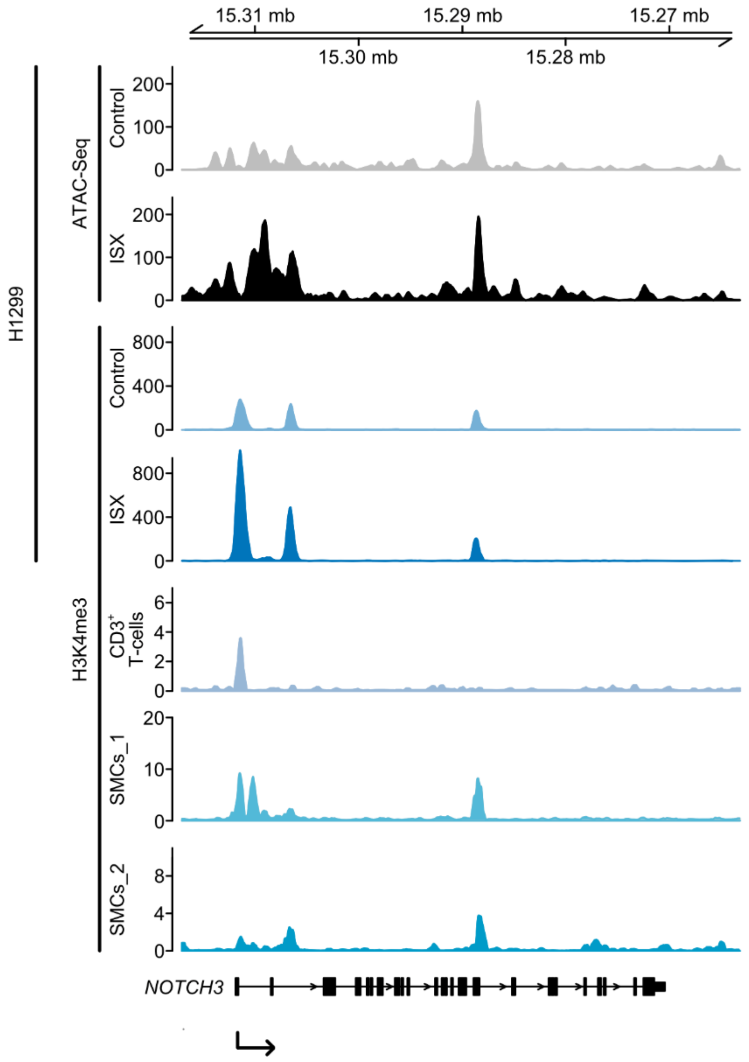

Supplementary Figure 5. The cryptic promoter at the *NOTCH3* locus found in primary human smooth muscles cells (SMCs) but not in primary human CD3^{+} T-cells. ENCODE ChIP-Seq data versus H3K4me3 in primary cells were analyzed to investigate the enrichment of this promoter mark at the *NOTCH3* locus. Shown are from top to bottom: ATAC-Seq in H1299 cells treated with DMSO as a control or with ISX; H3K4me3 ChIP-Seq in H1299 cells treated with DMSO as a control or with ISX; H3K4me3 ChIP-Seq in flow sorted CD3^{+} primary cells from cord blood of a 37 years male (ENCSR395YXN); (SMCs_1) H3K4me3 ChIP-Seq in human primary stomach smooth muscle tissue obtained from a healthy 84 years female donor (ENCSR532FEO) ; (SMCs_2) H3K4me3 ChIP-Seq in primary stomach smooth muscle tissue obtained from a 59 years old male (ENCSR168PQI)

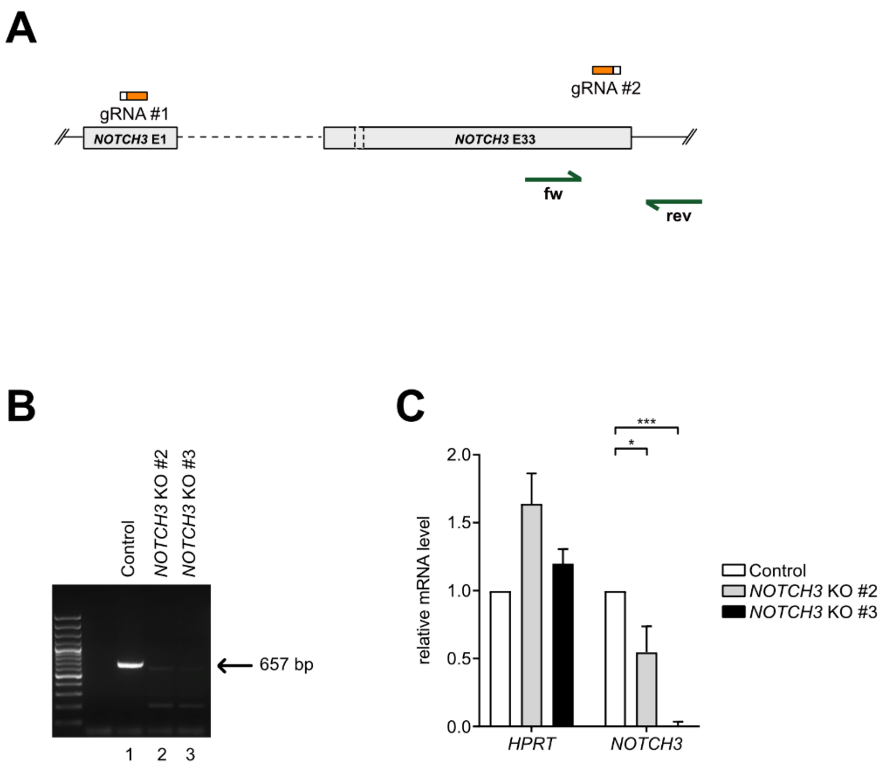

Supplementary Figure 6. *NOTCH3* is efficiently targeted in H1299 cells using the CRISPR/Cas9 technology. Depletion of NOTCH3 in H1299 cells was achieved using the CRISPR9/Cas9 technology. (**A**) Scheme of the CRISPR/Cas9 strategy used to target the *NOTCH3* locus. (**B**) Genomic DNA (gDNA) was purified and analyzed by PCR using primers specific for the *NOTCH3* locus. (**C**) Total RNA was purified, reverse transcribed and *NOTCH3* expression was analyzed by RT-qPCR using gene-specific primers for *HPRT* and *NOTCH3*. Data were normalized to the housekeeping gene *GAPDH* and represent the mean ± SD of three independent experiments (**P* < 0.05, ****P* < 0.001, unpaired Student's t-test).

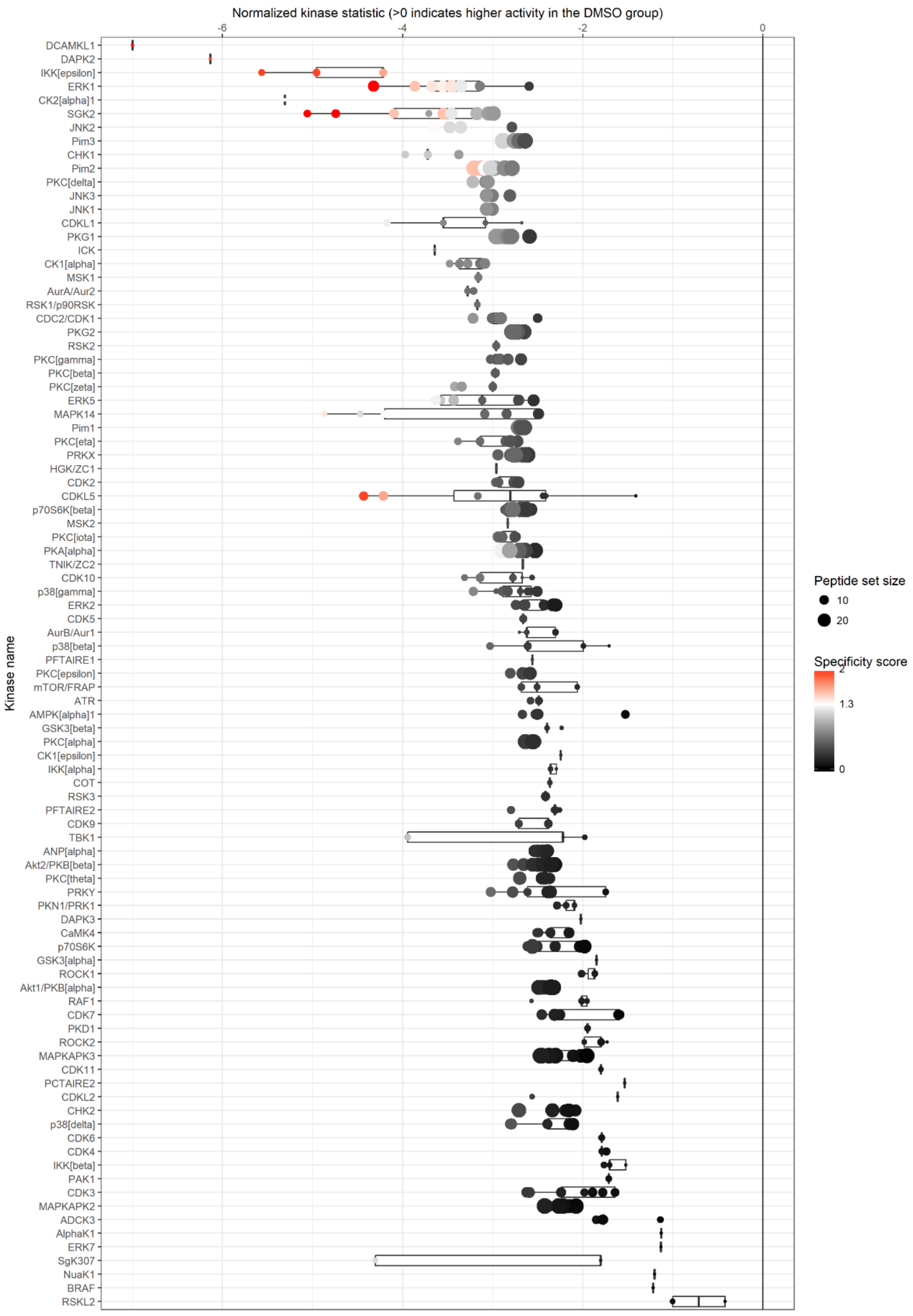

Supplementary Figure 7. Serine/threonine kinases are generally downregulated in DMSO control conditions compared to ISX-exposed H1299 cells. This plot shows all

putative upstream serine/threonine kinases accordingly to their normalized kinase statistics, *i.e.* predicted relative kinase activity, where top ranked kinases (*e.g.* DCAMKL1, DAPK2, and ERK1) are upregulated in ISX-treated cells compared to DMSO control conditions. In detail, the x-axis indicates the values for the normalized kinase statistic with values larger than 0 indicate a higher kinase activity in the DMSO treated control group compared to ISX exposed cells. Each point of the plot represents the outcome of an individual analysis with a varying rank-cut off for adding upstream kinases for peptides (permutation tests). The size of each dot depicts the size of the peptide set which was used for the corresponding analysis. Significance is defined by the mean specificity score [negative $\log_{10}$ (p value) with $p < 0.05$] where the p value refers to the statistical analysis by a t test between the respective peptide sets, *i.e.* their intensity of phosphorylation, that was used to predict the activity of the given kinase.

i.e. listed kinases are down in 30 min treatment			
Kinase Uniprot ID	Kinase Name	Mean Specificity Score	Median Kinase Statistic
P12931	**Src**	2,2235	2,1570
P29320	**EphA3**	2,0713	4,2525
P06213	**InSR**	1,7583	2,3144
P08631	**HCK**	1,7083	2,1569
P07947	**Yes**	1,5264	2,1730
P06241	**Fyn**	1,5045	2,3664
P07948	**Lyn**	1,4933	2,2436
P21709	**EphA1**	1,4929	3,7601
Q16288	**TRKC**	1,3109	2,1138
P52333	JAK3	1,2390	3,2010
P16234	PDGFR[alpha]	1,2365	3,0914
P06239	Lck	1,2051	1,9900
P10721	Kit	1,2048	2,1148
P54764	EphA4	1,1366	2,8830
P08069	IGF1R	1,1158	2,0275
P08581	Met	1,1109	1,7607
Q15303	HER4	1,0781	1,8480
P30530	Axl	1,0432	1,6354

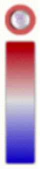

i.e. listed kinases are up in 30 min treatment			
Kinase Uniprot ID	Kinase Name	Mean Specificity Score	Median Kinase Statistic
O15075	**DCAMKL1**	2,27866	-6,99972
Q9UIK4	**DAPK2**	1,75799	-6,13398
Q14164	**IKK[epsilon]**	1,66524	-4,21699
P68400	**CK2[alpha]1**	1,34975	-5,30664
Q9HBY8	**SGK2**	1,34482	-3,54834
P27361	ERK1	1,21207	-3,45323
P45984	JNK2	1,10587	-3,35488
Q9P1W9	Pim2	1,04087	-2,98701
O14757	CHK1	1,00101	-3,72017

Supplementary Figure 8. Characterization of ISX-regulated kinases in H1299 cells. Serine/threonine kinases listed according to downregulated kinases after 30 minutes of treatment with 20 μM ISX (left side) and upregulated kinases after 30 minutes of treatment with 20 μM ISX (right side).

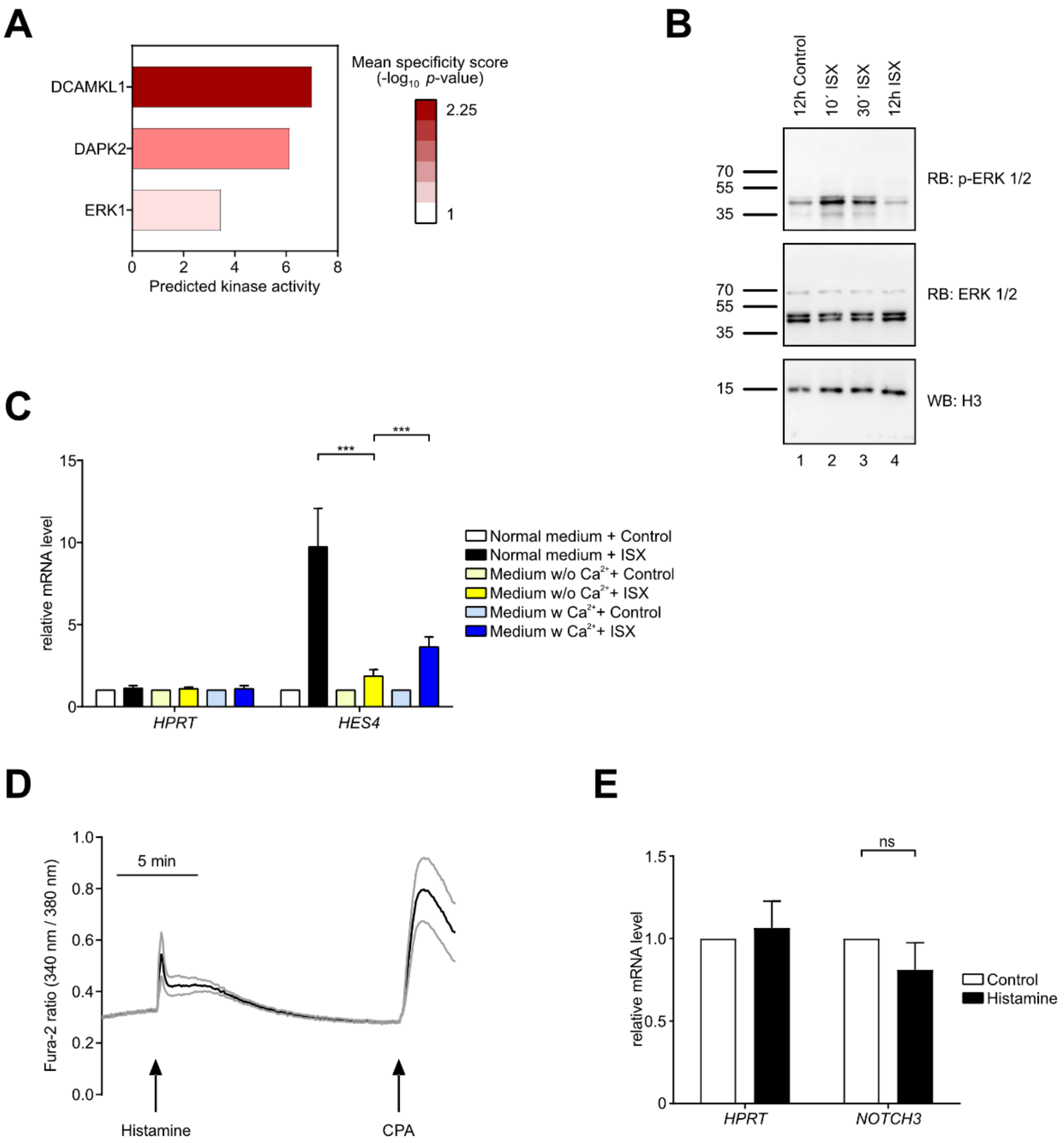

Supplementary Figure 9. Further **Characterization of intracellular ISX-effects in regard to kinases and Calcium-release:** (**A**) Kinome profiling reveals three kinases which show a deregulated activity in ISX-treated cells versus DMSO control conditions after 30 minutes of incubation. Predicted kinase activity is calculated on the basis of differential phosphorylation of particular substrate peptides presented on STK chips. Significance is defined by the mean specificity score [negative $\log_{10}$ (p value) with $p < 0.05$] where the p value refers to the statistical analysis by a t test between the respective peptide sets, *i.e.* their intensity of phosphorylation, that was used to predict the activity of the given kinase. (**B**) ISX treatment

leads to activation of ERK 1/2 in H1299 cells. WB = Western blot; RB = reblot. H1299 cells were treated with 20 μM ISX for 10 minutes, 30 minutes or 12 hours or with DMSO for 12 hours as a control. Whole cell extract (WCE) was analyzed by Western blotting versus p-ERK 1/2, ERK 1/2 or H3 as a loading control. (**C**-**E**) Induction of Ca^{2+} is not sufficient to induce *NOTCH3* in H1299 cells. (**C**) H1299 cells were treated with ISX for 12 hours in normal medium, medium depleted of Ca^{2+} (w/o) or medium depleted of Ca^{2+} and re-supplemented with Ca^{2+} (w). Total RNA was purified, reverse transcribed and Notch target gene expression was analyzed by RT-qPCR using gene-specific primers for *HPRT* and *HES4*. Data were normalized to the housekeeping gene *GAPDH* and represent the mean ± SD of five independent experiments (***$P < 0.001$, unpaired Student's t-test). (**D**-**E**) Histamine increases cytosolic Ca^{2+} in H1299 cells but does not lead to induction of *NOTCH3*. H1299 were treated with 100 μM of histamine or H_2O as a control for 24 hours. (**D**) Cytosolic Ca^{2+} level was measured as a quantification of Fura-2 (340/380) ratios shown as mean (black line) ± SEM (gray area) after stimulation with CPA or with histamine. (**E**) Total RNA was purified, reverse transcribed and *NOTCH3* expression was analyzed by RT-qPCR using gene-specific primers for *HPRT* and *NOTCH3*. Data were normalized to the housekeeping gene *GAPDH* and represent the mean ± SD of three independent experiments (ns = not significant, unpaired Student's t-test).

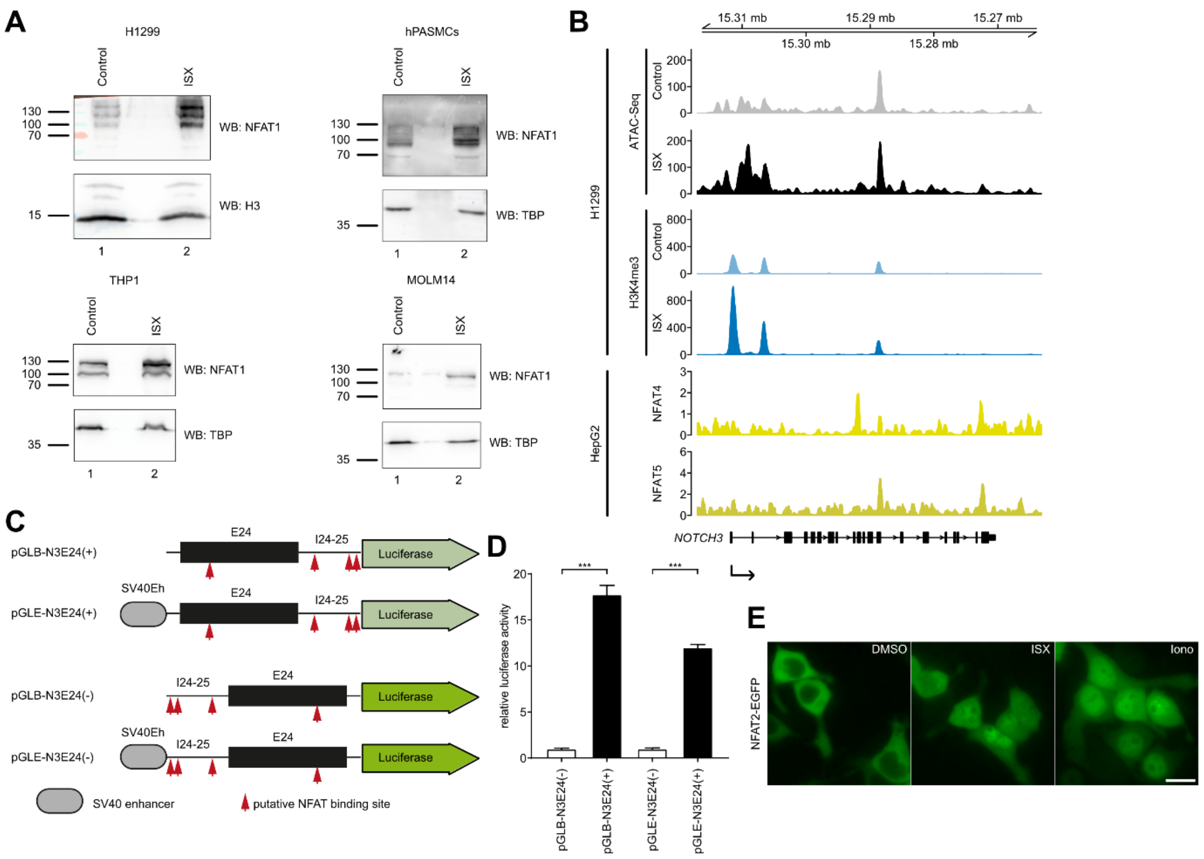

Supplementary Figure 10. Characterization of the NFAT-NOTCH3 link upon ISX treatment. (**A**) NFAT1 is induced upon ISX treatment. H1299 cells, human primary pulmonary artery smooth muscles cells (hPASMCs) and acute myeloid leukemia (AML) THP1 or MOLM14 cells were treated with 20 µM ISX or DMSO as a control (H1299: 12 hours; hPASMCs, THP1 and MOLM14: 24 hours). Nuclear extracts from H1299, THP1 and MOLM14 cells or whole cell extracts (WCE) from hPASMCs were analyzed by Western blotting versus NFAT1 and TBP or H3 as loading controls. (**B**) NFAT4 and NFAT5 transcription factors bind to the cryptic promoter of the *NOTCH3* locus in hepatocellular carcinoma HepG2 cells. ENCODE ChIP-Seq data versus NFAT4 or NFAT5 in hepatocellular carcinoma HepG2 cells were analyzed to investigate their binding at the cryptic promoter of the *NOTCH3* locus. Shown are from top to bottom: ATAC-Seq in H1299 cells treated with DMSO as a control or with ISX; H3K4me3 ChIP-Seq in H1299 cells treated with DMSO as a control or with ISX; NFAT4 ChIP-Seq in HepG2 cells (ENCSR635SKD); NFAT5 ChIP-Seq in HepG2 cells (ENCSR565BVI).

(C) Schematic representation of luciferase constructs of the cryptic promoter region within the Notch3 locus (between exon 24-25). Upper two constructs contain four putative NFAT binding

sites. (D) Basal activity of the luciferase constructs expressed in HeLa cells indicating a strong basal activity and dependence of directionality. E) Nuclear translocation of NFAT2 only occurs upon ISX-stimulation or PMA/ionomycin-stimulation but not in control DMSO-treated cells.

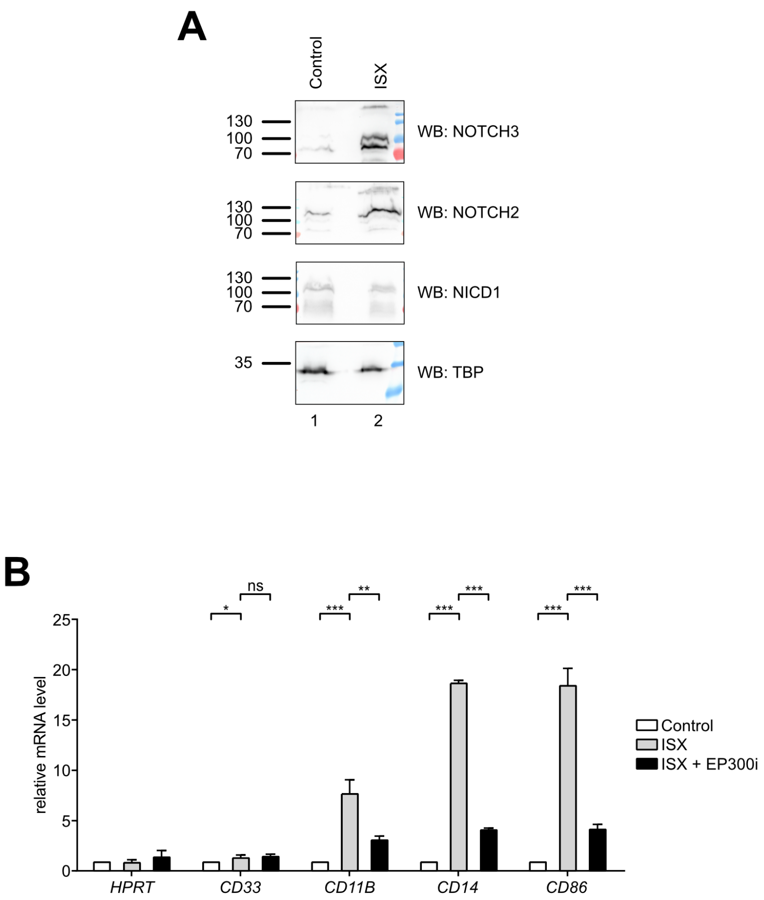

Supplementary Figure 11. (A) ISX promotes NOTCH2 and NOTCH3 but not NOTCH1 activation in THP1 cells. THP1 cells were treated with 20 µM ISX or DMSO as a control for 24 hours. Nuclear extract (NE) was analyzed by Western blotting versus NOTCH3, NOTCH2, cleaved NOTCH1 (NICD1) or TBP as a loading control. (B) The Notch-associated coactivator complex is required to activate the expression of differentiation markers in THP1. THP1 cells were treated with 20 µM ISX, with 20 µM ISX in combination with 10 µM EP300 inhibitor (EP300i) or DMSO as a control for 24 hours. Total RNA was isolated, reverse transcribed expression of differentiation markers was analyzed by RT-qPCR using gene-specific primers for *HPRT*, *CD33*, *CD11B*, *CD14* and *CD86*. Data were normalized to the housekeeping gene *GAPDH* and represent the mean ± SD of three experiments (**P* < 0.05, ***P* < 0.01, ****P* < 0.001, ns = not significant, unpaired Student’s t-test).

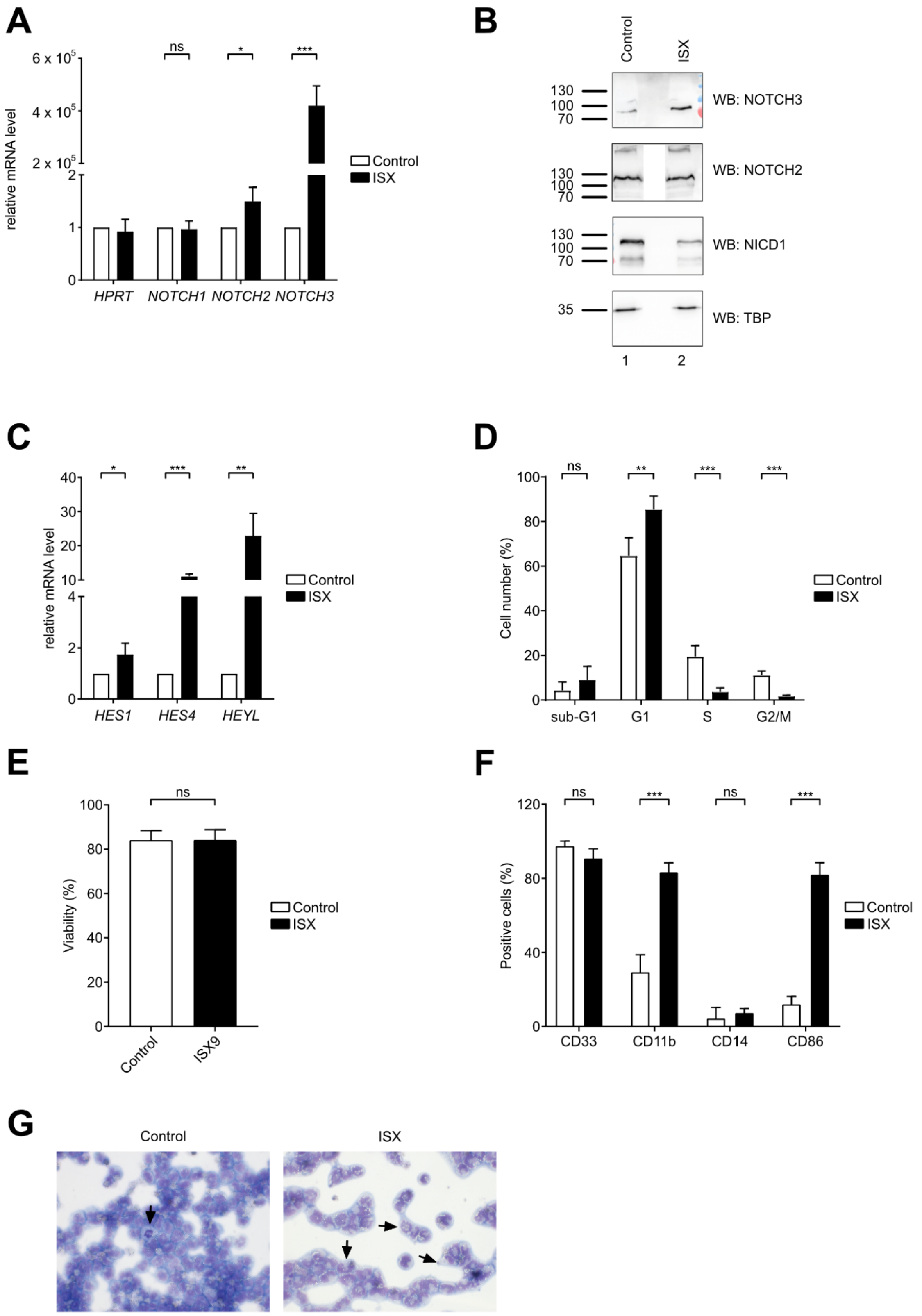

Supplementary Figure 12. ISX promotes cell cycle arrest and differentiation in MOLM14 cells. (**A-C**) MOLM14 cells were treated with 20 μM ISX or DMSO as a control for 24 hours.

(**A**) Total RNA was isolated, reverse transcribed and Notch target genes expression was analyzed by RT-qPCR using gene-specific primers for *HPRT*, *NOTCH1*, *NOTCH2* and *NOTCH3*. Data were normalized to the housekeeping gene *GAPDH* and represent the mean ± SD of three experiments (*P < 0.05, ***P < 0.001, ns = not significant, unpaired Student's t-test). (**B**) Nuclear extract (NE) was analyzed by Western blotting versus NOTCH3, NOTCH2, cleaved NOTCH1 (NICD1) or TBP as a loading control. (**C**) Total RNA was purified from MOLM14 cells, reverse transcribed and Notch target genes expression was analyzed by RT-qPCR using gene-specific primers for *HES1*, *HES4* and *HEYL*. Data were normalized to the housekeeping gene *GAPDH* and represent the mean ± SD of three experiments (*P < 0.05, **P < 0.01, ***P < 0.001, unpaired Student's t-test). (**D-G**) MOLM14 cells were treated with 10 µM ISX or DMSO as a control for 24 hours. (**D**) Cell cycle progression of MOLM14 cells is arrested by ISX. MOLM14 cells were fixed and then incubated with DAPI. Cell cycle dynamics of the ISX and DMSO-treated MOLM14 cells were determined by the DNA content detected by DAPI staining. Shown is the mean ± SD of four independent experiments (**P < 0.01, ***P < 0.001, ns = not significant, unpaired Student's t-test). (**E**) Cell viability of MOLM14 cells remains unaffected upon ISX treatment (ns = not significant, unpaired Student's t-test). (**F**) ISX strongly promotes the surface expression of differentiation markers in MOLM14 cells. Levels of surface antigens CD33, CD11b, CD14, and CD86 were analyzed by flow cytometry. Shown is the mean ± SD of three to six independent experiments (***P < 0.001, ns = not significant, unpaired Student's t-test). (**G**) Differentiation of MOLM14 cells was also observed morphologically. MOLM14 cells were treated with ISX or DMSO, cytospin was performed followed by May-Grünwald Giemsa staining. Differentiated cells are indicated by arrows.

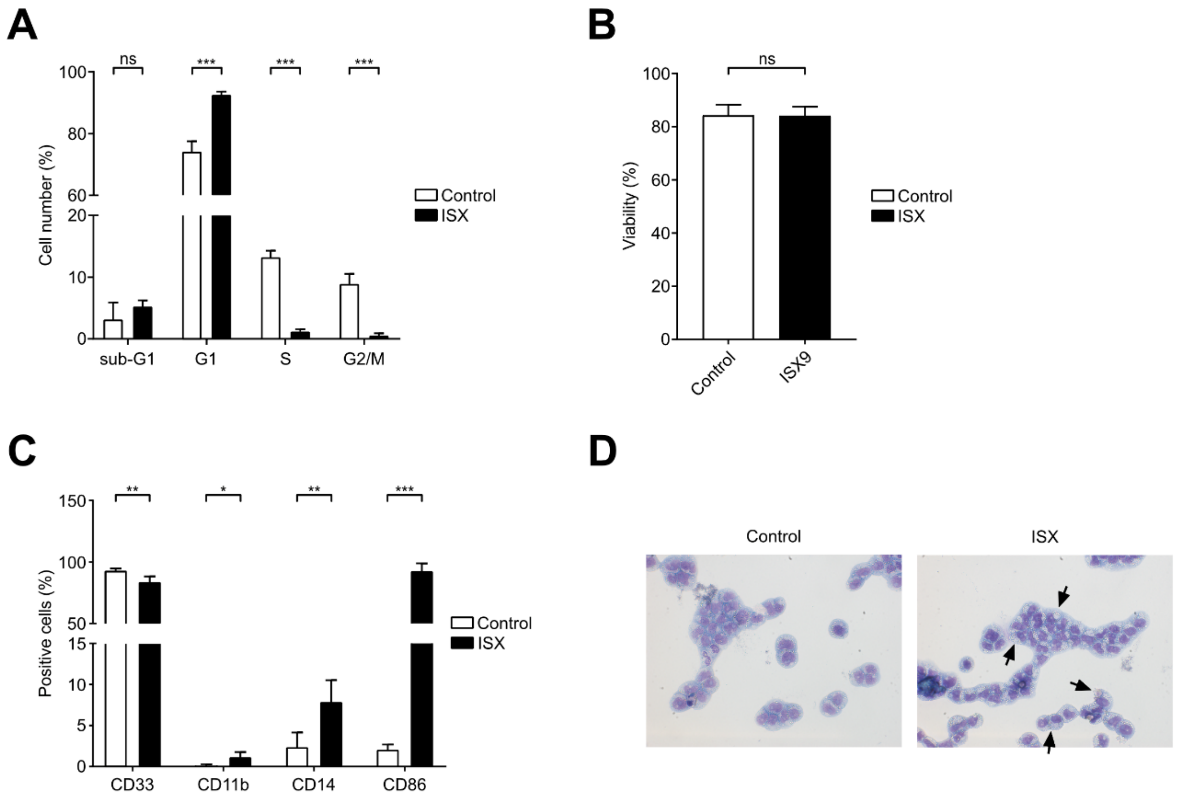

Supplementary Figure 13. ISX promotes cell cycle arrest and differentiation in Kasumi cells. (**A**-**D**) Kasumi cells were treated with 10 µM ISX or DMSO as a control for 24 hours. (**A**) Cell cycle progression of Kasumi cells is arrested by ISX. Kasumi cells were fixed and then incubated with DAPI. Cell cycle dynamics of the ISX and DMSO-treated Kasumi cells were determined by the DNA content detected by DAPI staining. Shown is the mean ± SD of four independent experiments (****P* < 0.001, ns = not significant, unpaired Student's t-test). (**B**) Kasumi cells viability remains unaffected upon ISX treatment (ns = not significant, unpaired Student's t-test). (**C**) ISX strongly promotes the surface expression of differentiation markers in Kasumi cells. Levels of surface antigens CD33, CD11b, CD14, and CD86 were analyzed by flow cytometry. Shown is the mean ± SD of three to five independent experiments (**P* < 0.05, ***P* < 0.01, ****P* < 0.001, unpaired Student's t-test). (**D**) Differentiation of Kasumi cells was also observed morphologically. Kasumi cells were treated with ISX or DMSO, cytospin was performed followed by May-Grünwald Giemsa staining. Differentiated cells are indicated by arrows.

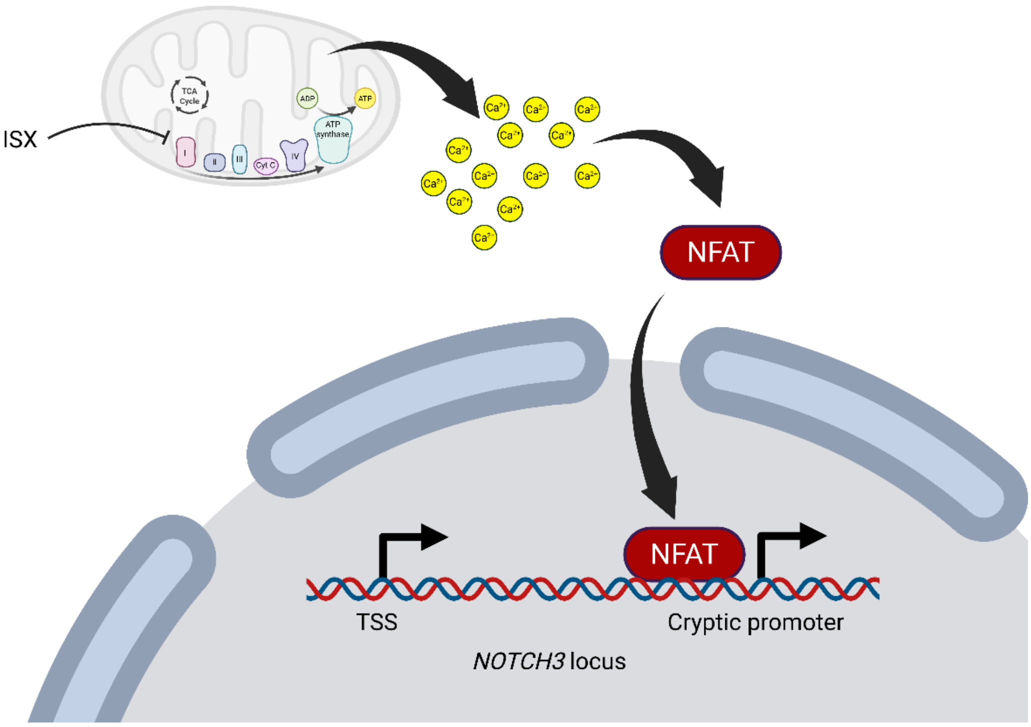

Supplementary Figure 14. Model for the ISX-mediated regulation of the *NOTCH3* locus. ISX inhibits the mitochondrial complex I leading to calcium (Ca^{2+}) release from the mitochondria. Ca^{2+} leads to nuclear stabilization of NFAT proteins which activate the cryptic promoter at the NOTCH3 locus finally resulting in a γ-secretase-independent NOTCH3-mediated pathway activation. Created in BioRender. Giaimo, B. (2026) https://BioRender.com/6eu7e67

SUPPLEMENTARY TABLES

Supplementary Table 1. DESeq2 results and normalized read counts per gene for ISX (12 or 24 hours vs DMSO) and RBPJ depletion (clone B6 vs eV control) measured by RNA-Seq.

Supplementary Table 2. Over-representation analyses based on GO ["Biological Process" (BP)] database for genes upregulated by ISX (12 and 24 hours) in H1299 cells**.**

Supplementary Table 3. Gene Set Enrichment Analysis (GSEA) for genes deregulated by ISX (12 or 24 hours) in H1299 cells.

Supplementary Table 4. Table showing all identified RBPJ binding sites in H1299 cells using ChIP-Seq.

Supplementary Table 5. Increase of cytosolic Ca^{2+} (Δ Fura-2 ratio, 340/380 nm) and number of responding cells to respective agonist in Ca^{2+} containing and Ca^{2+} free buffer (Tyrode's solution). Cells were stimulated with 20 µM ISX or 10 µM cyclopiazonic acid (CPA, viability control); 0.01 % DMSO was used as a control. Δ Fura-2 ratio are means ± SEM (from at least 3 independent experiments). *p < 0.05 (student's t-test, compared to Ca^{2+} free conditions with the same agonist), $^{\#}$ $p < 0.05$ (χ^2 test, compared to DMSO alone).

	Agonist			CPA		
	Δ Fura-2-ratio	Responder		Δ Fura-2-ratio	Responder	
		n	%		n	%
**Ca^{2+} buffer**						
ISX	0.52 ± 0.04*	65/116	56	1.67 ± 0.18*	87/116	75

Control	0.17	1/83#	1	1.56 ± 0.18	83/83	100
**Ca^{2+}-free buffer**						
ISX	0.32 ± 0.03	43/62	69	0.42 ± 0.04	54/62	87
Control	0.23 ± 0.02	19/59#	32	0.89 ± 0.09	57/59	97

Supplementary Table 6. Serine/threonine kinases, differentially regulated in ISX-treated cells compared to DMSO control. Predicted relative kinase activity given as arbitrary units is increased under ISX treatment. Significance is defined by the mean specificity score [negative $\log_{10}$ (*p* value) with $p < 0.05$] where the *p* value refers to the statistical analysis by a *t* test between the respective peptide sets, *i.e.* their intensity of phosphorylation, that were used to predict the activity of the given kinase. The list includes only kinases with significant changes in activity ($p < 0.05$) as well as those that only show a trend ($p < 0.1$, depicted in grey italic font).

Kinase name	Predicted relative kinase activity	Activity in ISX treated cells versus DMSO control	Mean Specificity Score	p value	Kinase Uniprot ID
**CK2[alpha]1**	5.31	Up under ISX treatment	1.35	0.045	P68400
**DAPK2**	6.13	Up under ISX treatment	1.76	0.017	Q9UIK4
**DCAMKL1**	7.00	Up under ISX treatment	2.28	0.005	O15075
**IKK[epsilon]**	4.22	Up under ISX treatment	1.67	0.022	Q14164
**SGK2**	3.55	Up under ISX treatment	1.34	0.045	Q9HBY8
CHK1	*3.72*	Up under ISX treatment	*1.00*	*0.100*	*O14757*
ERK1	*3.45*	Up under ISX treatment	*1.21*	*0.061*	*P27361*
JNK2	*3.35*	Up under ISX treatment	*1.11*	*0.078*	*P45984*
Pim2	*2.99*	Up under ISX treatment	*1.04*	*0.091*	*Q9P1W9*

Supplementary Table 7. Tyrosine kinases, differentially regulated in ISX-treated cells compared to DMSO control. Predicted relative kinase activity given as arbitrary units is reduced under ISX treatment. Significance is defined by the mean specificity score [negative $\log_{10}$ (*p* value) with $p < 0.05$] where the *p* value refers to the statistical analysis by a *t* test between the respective peptide sets, *i.e.* their intensity of phosphorylation, that were used to predict the activity of the given kinase. The list includes only kinases with significant changes in activity ($p < 0.05$) as well as those that only show a trend ($p < 0.1$, depicted in grey italic font).

**Kinase name**	**Predicted relative kinase activity**	**Activity in ISX treated cells versus DMSO control**	**Mean Specificity Score**	**p value**	**Kinase Uniprot ID**
**EphA1**	-3.76	Down under ISX treatment	1.49	0.032	P21709
**EphA3**	-4.25	Down under ISX treatment	2.07	0.008	P29320
**Fyn**	-2.37	Down under ISX treatment	1.50	0.031	P06241
**HCK**	-2.16	Down under ISX treatment	1.71	0.020	P08631

**InSR**	-2.31	Down under ISX treatment	1.76	0.017	P06213
**Lyn**	-2.24	Down under ISX treatment	1.49	0.032	P07948
**Src**	-2.16	Down under ISX treatment	2.22	0.006	P12931
**TRKC**	-2.11	Down under ISX treatment	1.31	0.049	Q16288
**Yes**	-2.17	Down under ISX treatment	1.53	0.030	P07947
Axl	*-1.64*	Down under ISX treatment	*1.04*	*0.091*	*P30530*
EphA4	*-2.88*	Down under ISX treatment	*1.14*	*0.073*	*P54764*
HER4	*-1.85*	Down under ISX treatment	*1.08*	*0.084*	*Q15303*
IGF1R	*-2.03*	Down under ISX treatment	*1.12*	*0.077*	*P08069*

JAK3	*-3.20*	Down under ISX treatment	*1.24*	*0.058*	*P52333*
Kit	*-2.11*	Down under ISX treatment	*1.20*	*0.062*	*P10721*
Lck	*-1.99*	Down under ISX treatment	*1.21*	*0.062*	*P06239*
Met	*-1.76*	Down under ISX treatment	*1.11*	*0.077*	*P08581*
PDGFR[alpha]	*-3.09*	Down under ISX treatment	*1.24*	*0.058*	*P16234*

Supplementary Table 8. Oligos used for CRISPR/Cas9 cloning and primers used for CRISPR/Cas9 screening and RT-qPCR experiments.

**CRISPR/Cas9 guides**	
hRBPJ gRNA #1	5'- TCA TGC CAG TTC ACA GCA GT -3'*
hRBPJ gRNA #2	5'- TCC TTC TAC ATG CAA GTA TC -3'*
hNOTCH3 gRNA #1	5'-GTG GCG GCG ACA TCG GGC GA -3'*
hNOTCH3 gRNA #2	5'- TAT CAG TGG CCA ATT CGA GG -3'*
**CRISPR/Cas9 screening**	
hRBPJ screen fw	5'- ATC ATC TGT ACT GTC TTG G -3'

hRBPJ screen rev	5‘- AGA TGA ATA AAA AAG GCT CC -3‘	
hNOTCH3 screen fw	5‘- CAG GGC TAA CTT GAG CTC CC -3‘	
hNOTCH3 screen rev	5‘- CCA GGA GCA CTC CAA CTG AC -3‘	
**Cloning**		
mNFAT2_fw	5´-ATG TTA CCA TGC CAA GTA CCA GCT TTC CAG -3´	
mNFAT2_rev	5´-ATC TCG AGG TAA AAA CCT CCT CTC AGC TCA -3´	
**RT-qPCR**		
	Homo sapiens	Probe
CD11B fw	5‘- TCGGGTCATGCAGCATCAAT -3‘	82
CD11B rev	5‘- CACCAAGAACACCAGGCTGA -3‘	
CD14 fw	5‘- ACTGACGCTCGAGGACCTAA -3‘	34
CD14 rev	5‘- AGCTGGAAAGTGCAAGTCCT -3‘	
CD33 fw	5‘- GACCAGAGCAGGAGTGGTTC -3‘	17
CD33 rev	5‘- TGAGGCAGAGACAAAGAGCG -3‘	
CD86 fw	5‘- ACTGACGCTCGAGGACCTAA -3‘	SybrGreen
CD86 rev	5‘- AGCTGGAAAGTGCAAGTCCT -3‘	
GAPDH fw	5‘- ACACCCACTCCTCCACCTTT -3‘	45
GAPDH rev	5’- TGACAAAGTGGTCGTTGAGG -3‘	
HES1 fw	5’- GAAGCACCTCCGGAACCT -3‘	60
HES1 rev	5’- GTCACCTCGTTCATGCACTC -3‘	
HES4 fw	5‘- GCTCAGCTCAAAACCCTCAT -3‘	78
HES4 rev	5‘- CTCACGGTCATCTCCAGGAT -3‘	
HEY1 fw	5’- CAGGGAGCCAGCATGAAG -3’	17
HEY1 rev	5’- GAGCCGAACTCAAGTTTCCA -3’	
HEY2 fw	5’- CCAGCAGTGCATCAGTATGTC -3’	60

HEY2 rev	5’- CAGGCACTTACGAAACACGA -3’	
HEYL fw	5’- TCCCCACTGCCTTTGAGA -3’	78
HEYL rev	5’- TTTCAAGTGATCCACCGTCA -3’	
HPRT fw	5’- TGACCTTGATTTATTTTGCATACC -3’	73
HPRT rev	5’- CATCTCGAGCAAGACGTTCA -3’	
NOTCH1 fw	5’- GTGTGCACTGCGAGGTCA -3’	27
NOTCH1 rev	5’- CACAGATGCCCAGTGAAGC -3’	
NOTCH2 fw	5’- GACATTGATGACTGCCTTGC -3’	51
NOTCH2 rev	5’- GGCACTTATCCCCAGTGAAA -3’	
NOTCH3 fw	5’- CCTAGTCCTGGCTCCGAAC -3’	67
NOTCH3 rev	5’- CATCGGGGAAGCAGTGAT -3’	
RBPJ fw	5’- CAGCAAGCGGATAAAAGTCA -3’	21
RBPJ rev	5’- AACTGTCTGGGATCGTAGTCG -3’	
	Danio rerio	
her3.fwd	5’-AGCGCCTGTTCTAACAATATCC-3’	
her3.rev	5’-TGCAGATGCCTCAGATGTTTCA-3’	
rpl13.fwd	5’-TCTGGAGGACTGTAAGAGGTATGC-3’	
rpl13.rev	5’-AGACGCACAATCTTGAGAGCAG-3’	
slc25a5.fwd	5’-CTTCCTCAGCAGTCAATTGTGCC-3’	
slc25a5.rev	5’-GTAACCTGGCTTCAGGTGGTGCTG-3’	

Supplementary Table 9. Antibodies and isotype controls used for FACS analysis.

Antibody	Label	Supplier	Cat
anti-human CD11b	PE	Thermo Fisher	#12-0118-42
anti-human CD33	FITC	Thermo Fisher	#MA5-16981
anti-human CD86	APC	Thermo Fisher	#MA1-10294
anti-human CD14	APC	BD Biosciences	# 557831

mouse IgG1 isotype control	APC	Thermo Fisher	MA5-18093
mouse IgG2b isotype control	PE	BD Biosciences	# 559529
human Fc block	n.a.	BD Biosciences	# 564220

Supplementary Table 10. NOTCH3 specific oligonucleotides used for amplification of transcript specific fragments.

**Name**	**Sequence**
F1	5´-TGGCTACAATGGTGATAACTGTGAGG-3´
F2	5´-GCTGAGTGGGTCCCTCTCTTACC-3´
F3	5´-GAGAGGCTGTCTTCATTCCCTCTTG-3´
F4	5´-CCATTGTGGCTGATCTACATGCTCC-3´
F5	5´-ATGCCTGTAATCCCAGCTACTTGG-3´
F6	5´-AGCAAGACACGTTTTAGGGGTGC-3´
F8	5´-GTGGAGCGCCTGGACTTCC-3´
R1	5´-CTCGCAGGGAGACAGGACAG-3´

Supplementary References:

1. Kessler M, Berger IM, Just S, Rottbauer W. Loss of dihydrolipoyl succinyltransferase (DLST) leads to reduced resting heart rate in the zebrafish. Basic Res Cardiol. 2015;110(2):14.
2. Westerfield M. The Zebrafish Book: A Guide for the Laboratory Use of Zebrafish (Brachydanio Rerio). M. Westerfield. Book. 1993.
3. Kawakami K. Transgenesis and gene trap methods in zebrafish by using the Tol2 transposable element. Methods Cell Biol. 2004;77:201-22.
4. Liu W, Morgan KM, Pine SR. Activation of the Notch1 Stem Cell Signaling Pathway during Routine Cell Line Subculture. Front Oncol. 2014;4:211.
5. Cong L, Ran FA, Cox D, Lin S, Barretto R, Habib N, et al. Multiplex genome engineering using CRISPR/Cas systems. Science. 2013;339(6121):819-23.
6. Spinazzi M, Casarin A, Pertegato V, Salviati L, Angelini C. Assessment of mitochondrial respiratory chain enzymatic activities on tissues and cultured cells. Nat Protoc. 2012;7(6):1235-46.
7. Weiss A, Neubauer MC, Yerabolu D, Kojonazarov B, Schlueter BC, Neubert L, et al. Targeting cyclin-dependent kinases for the treatment of pulmonary arterial hypertension. Nat Commun. 2019;10(1):2204.
8. Kim D, Paggi JM, Park C, Bennett C, Salzberg SL. Graph-based genome alignment and genotyping with HISAT2 and HISAT-genotype. Nat Biotechnol. 2019;37(8):907-15.
9. Love MI, Huber W, Anders S. Moderated estimation of fold change and dispersion for RNA-seq data with DESeq2. Genome Biol. 2014;15(12):550.
10. Yu G, Wang LG, Han Y, He QY. clusterProfiler: an R package for comparing biological themes among gene clusters. OMICS. 2012;16(5):284-7.

11. Ferrante F, Giaimo BD, Bartkuhn M, Zimmermann T, Close V, Mertens D, et al. HDAC3 functions as a positive regulator in Notch signal transduction. Nucleic Acids Res. 2020;48(7):3496-512.
12. Ramirez F, Dundar F, Diehl S, Gruning BA, Manke T. deepTools: a flexible platform for exploring deep-sequencing data. Nucleic Acids Res. 2014;42(Web Server issue):W187-91.
13. Zhang Y, Liu T, Meyer CA, Eeckhoute J, Johnson DS, Bernstein BE, et al. Model-based analysis of ChIP-Seq (MACS). Genome Biol. 2008;9(9):R137.
14. Yu G, Wang LG, He QY. ChIPseeker: an R/Bioconductor package for ChIP peak annotation, comparison and visualization. Bioinformatics. 2015;31(14):2382-3.
15. Hahne F, Ivanek R. Visualizing Genomic Data Using Gviz and Bioconductor. Methods Mol Biol. 2016;1418:335-51.
16. Bailey TL, Johnson J, Grant CE, Noble WS. The MEME Suite. Nucleic Acids Res. 2015;43(W1):W39-49.
17. Gleason AC, Ghadge G, Chen J, Sonobe Y, Roos RP. Machine learning predicts translation initiation sites in neurologic diseases with nucleotide repeat expansions. PLoS One. 2022;17(6):e0256411.
