# Supplementary material for: Small molecule-mediated activation of Notch signal transduction
Source: Cell Death Dis. 2026 Jul 2;17(1):653. doi: 10.1038/s41419-026-09044-x (PMC13396519; doi:10.1038/s41419-026-09044-x)

**FIGURE 2A**


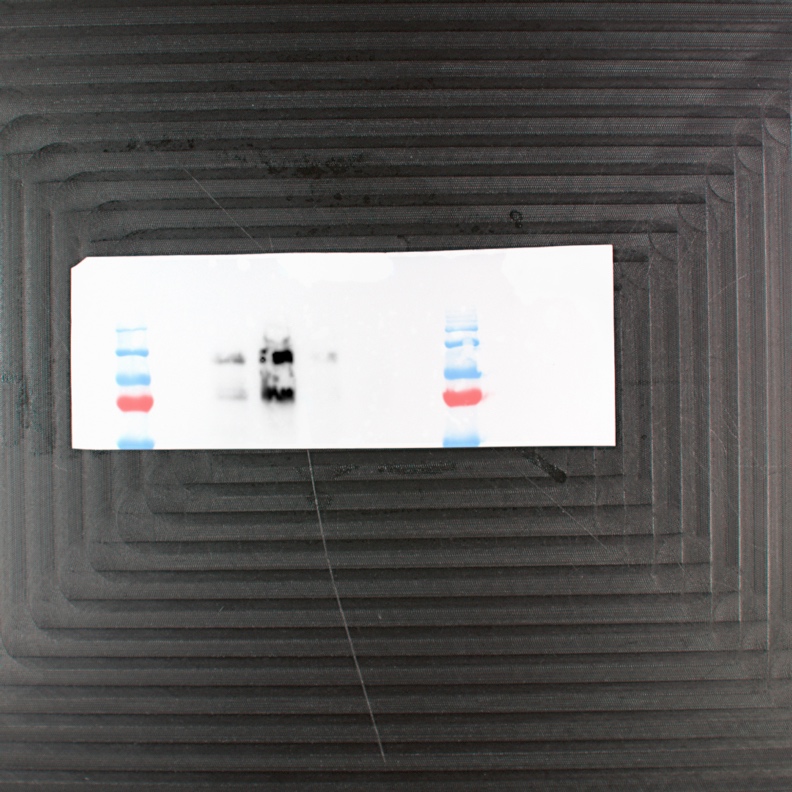

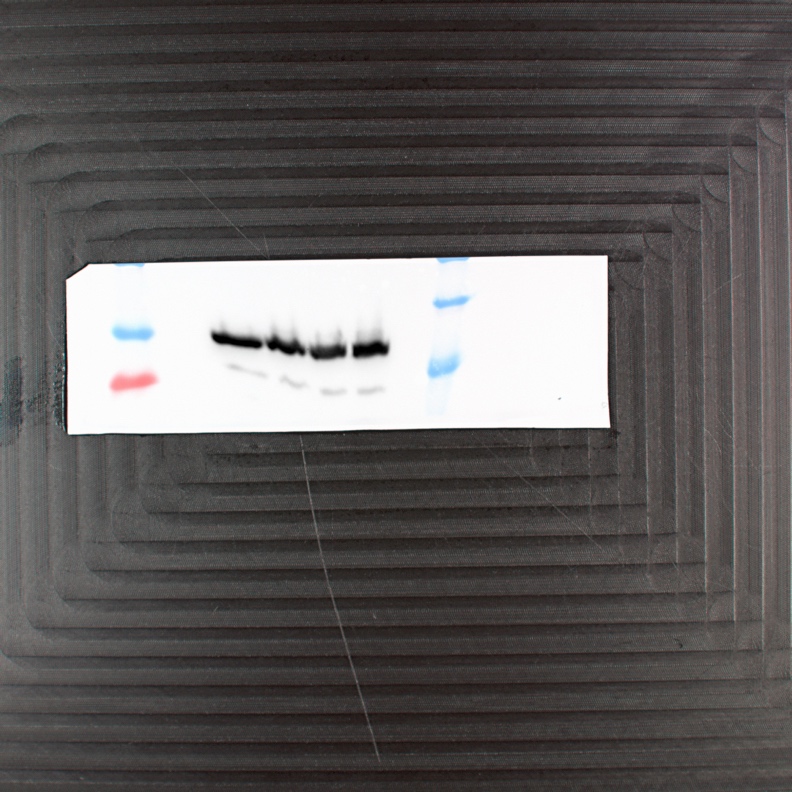


**FIGURE 2D**


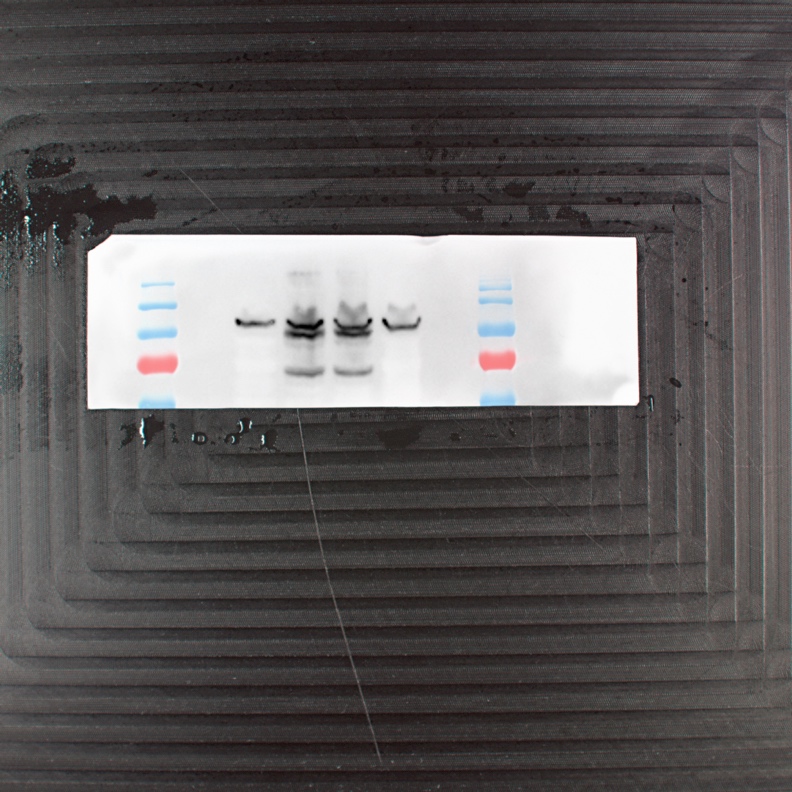

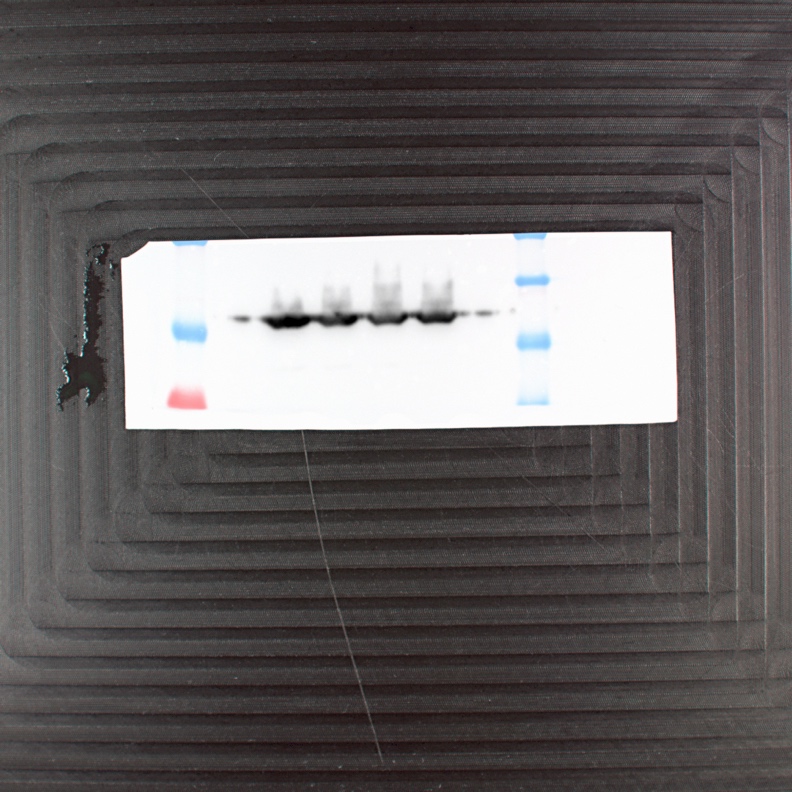


**FIGURE 3H**


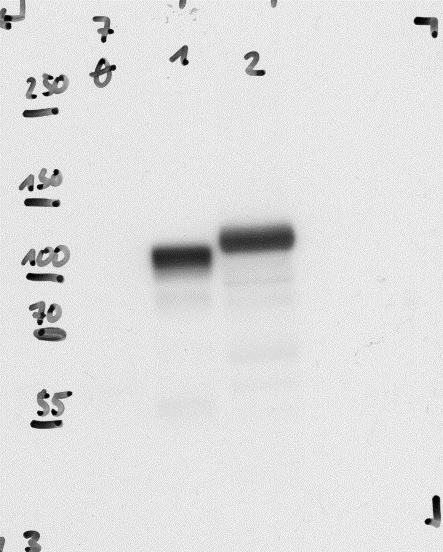


**Figure 4A**


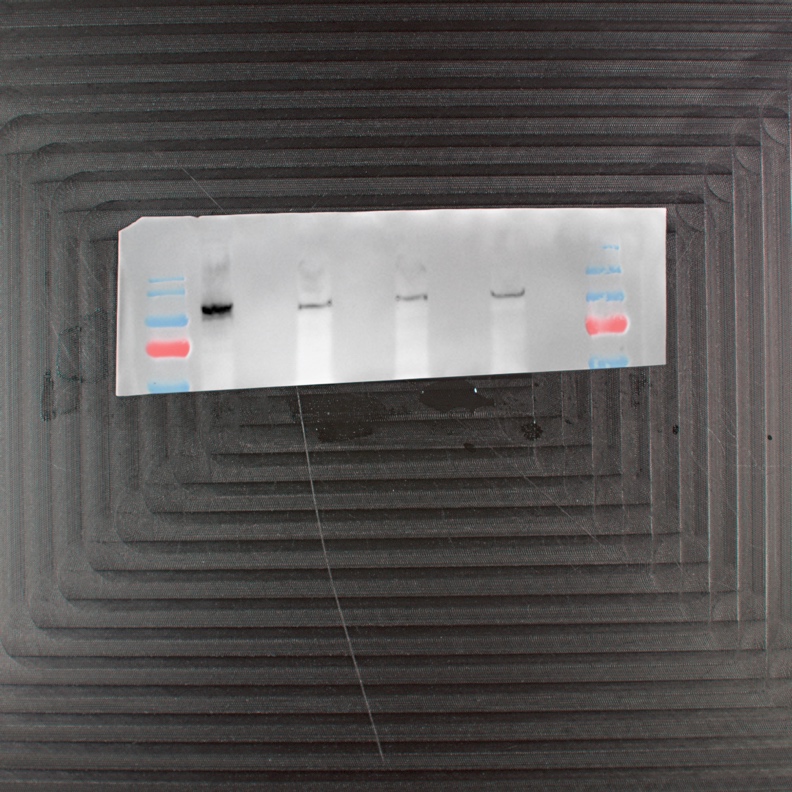

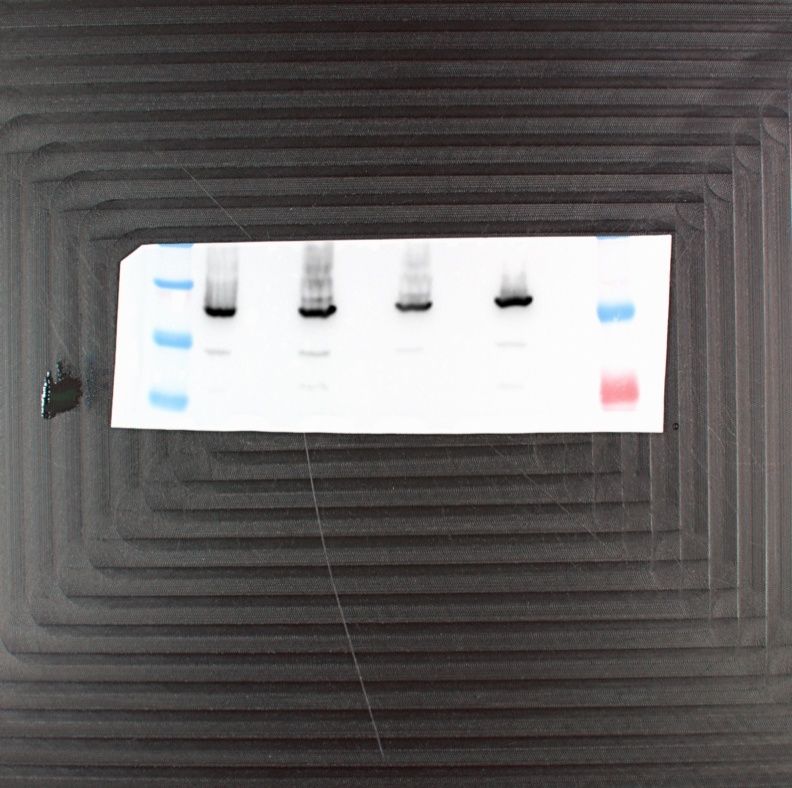


**Figure 4C**


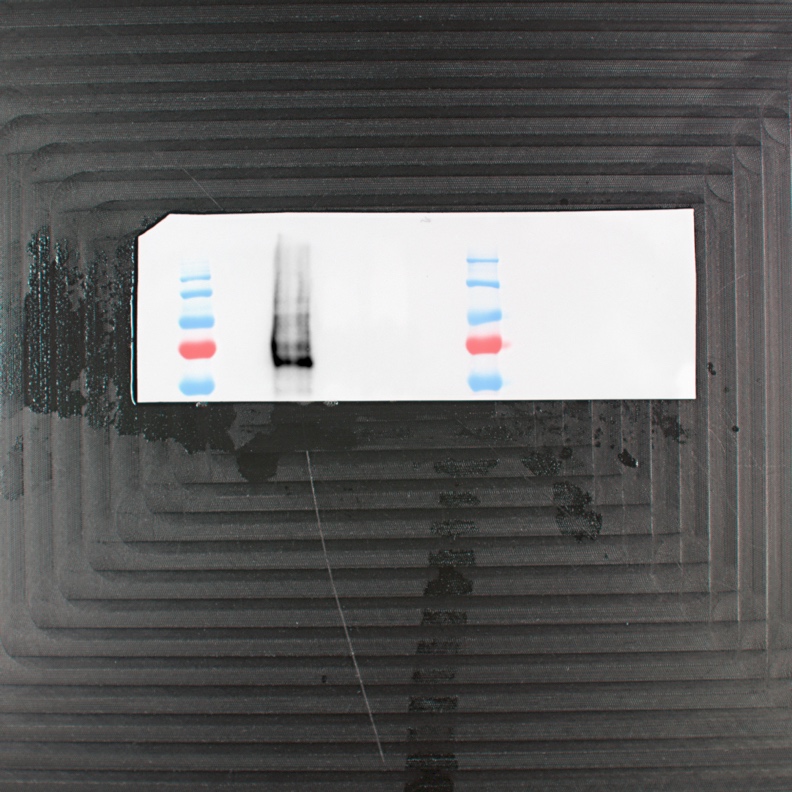

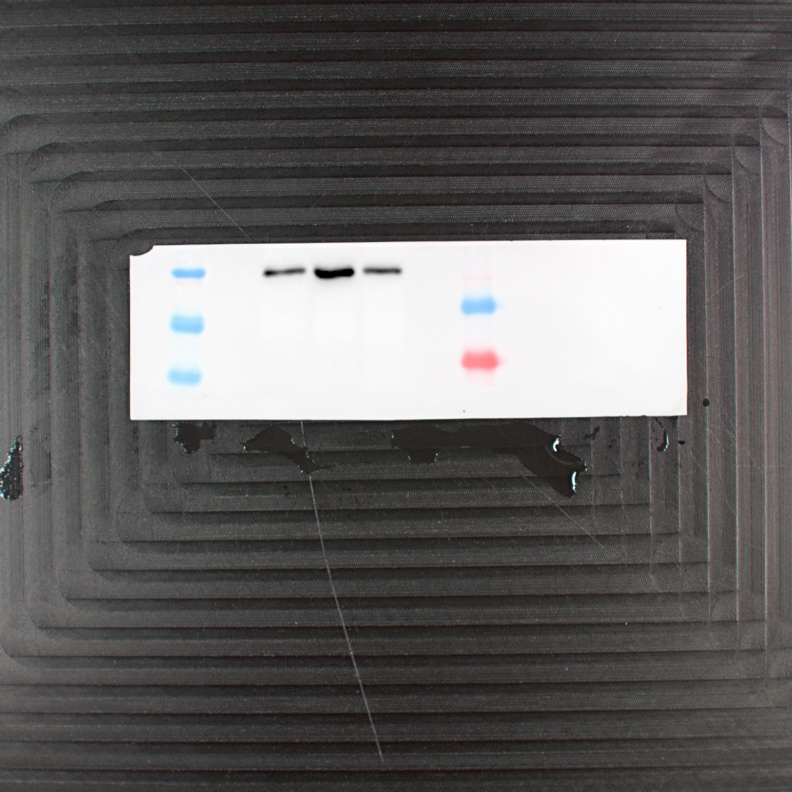


**Figure 4E**


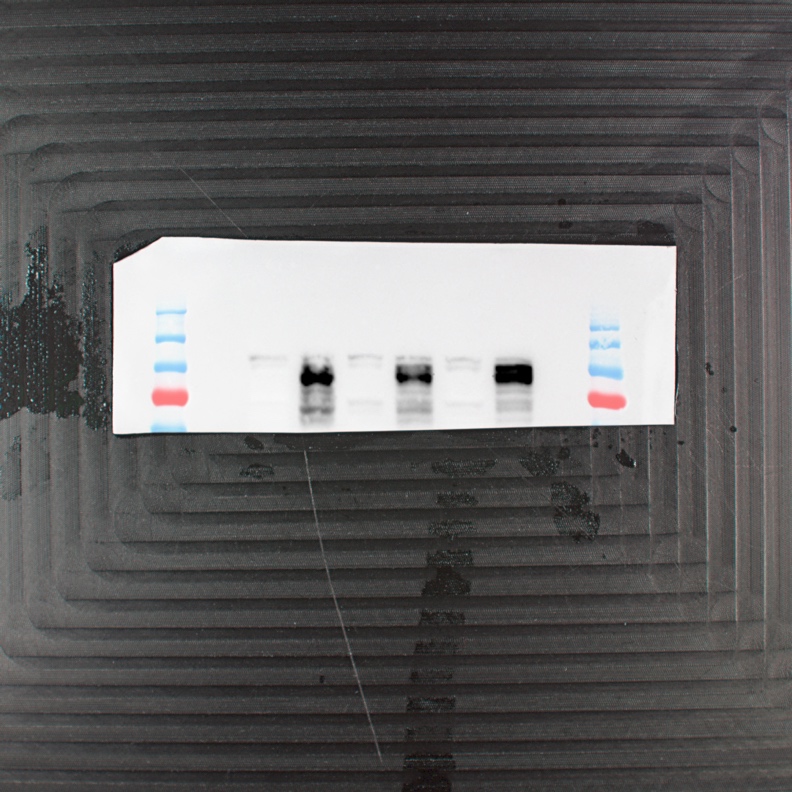


1

2

3

4

5

6


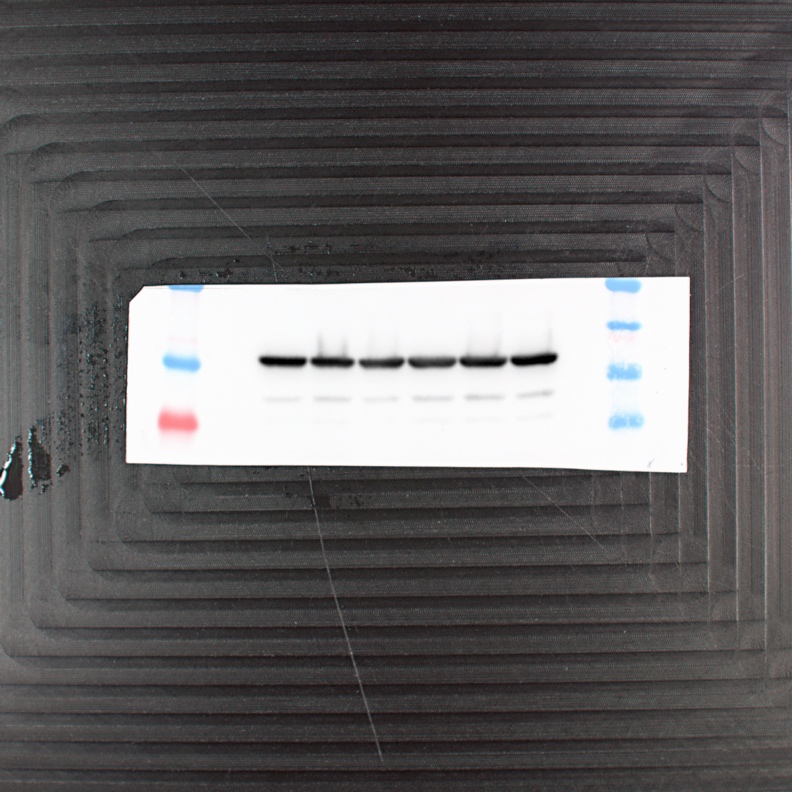


**Figure 5F**


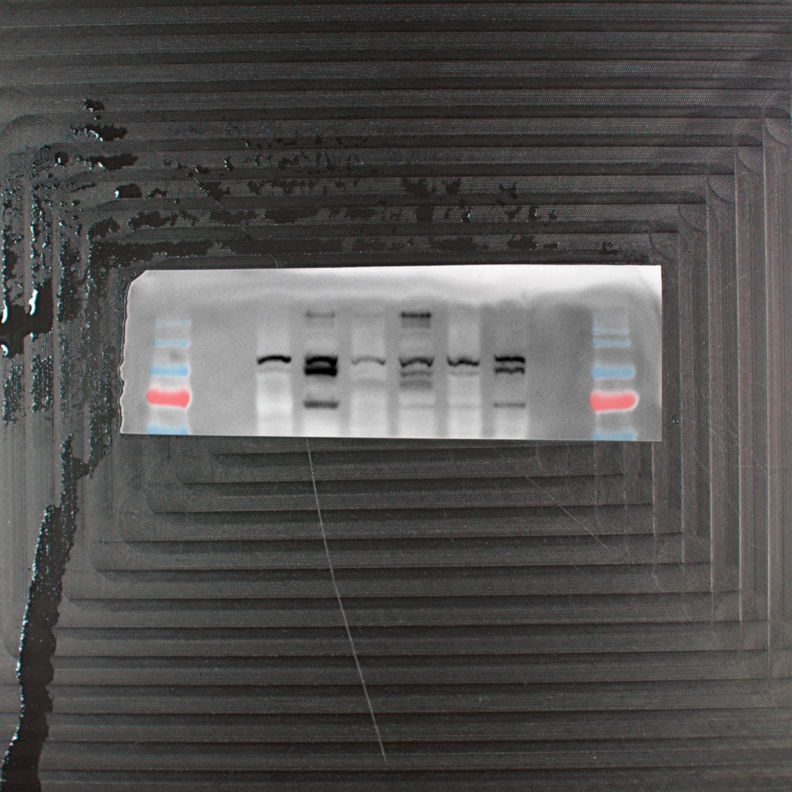

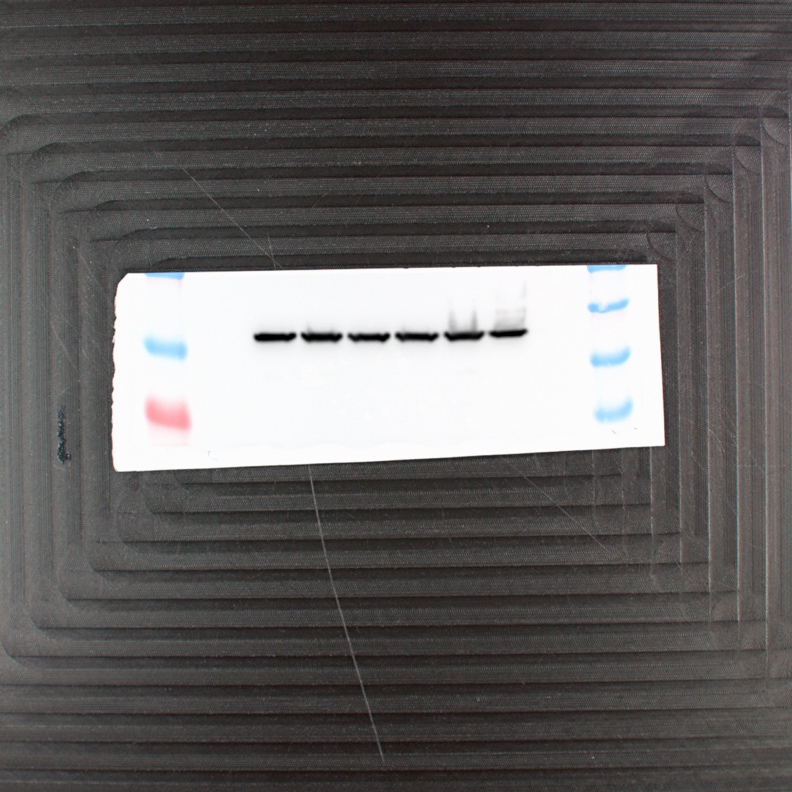


**Figure 6B**

1

2

3

4


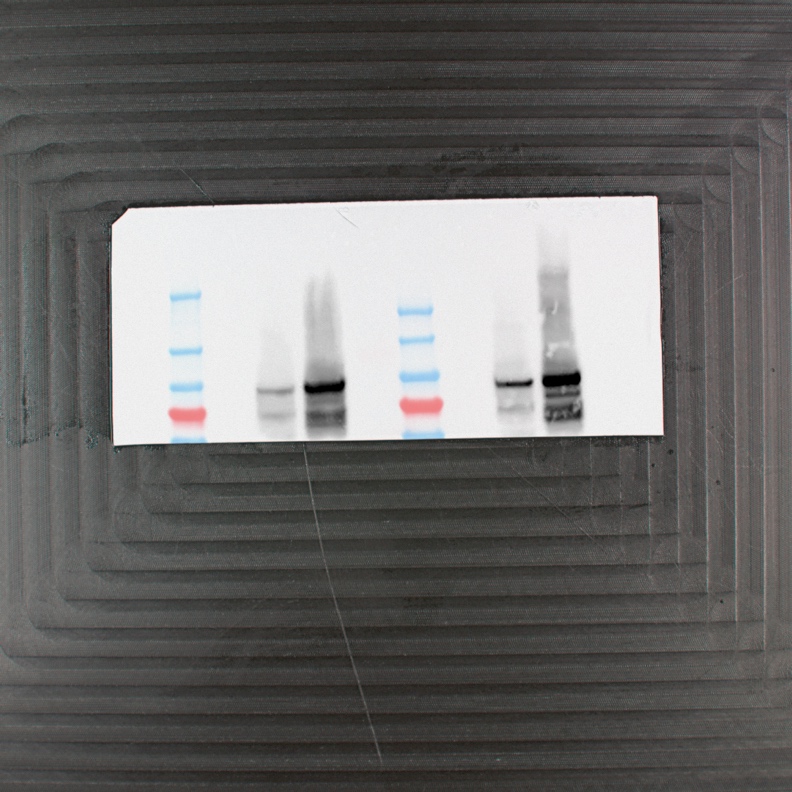

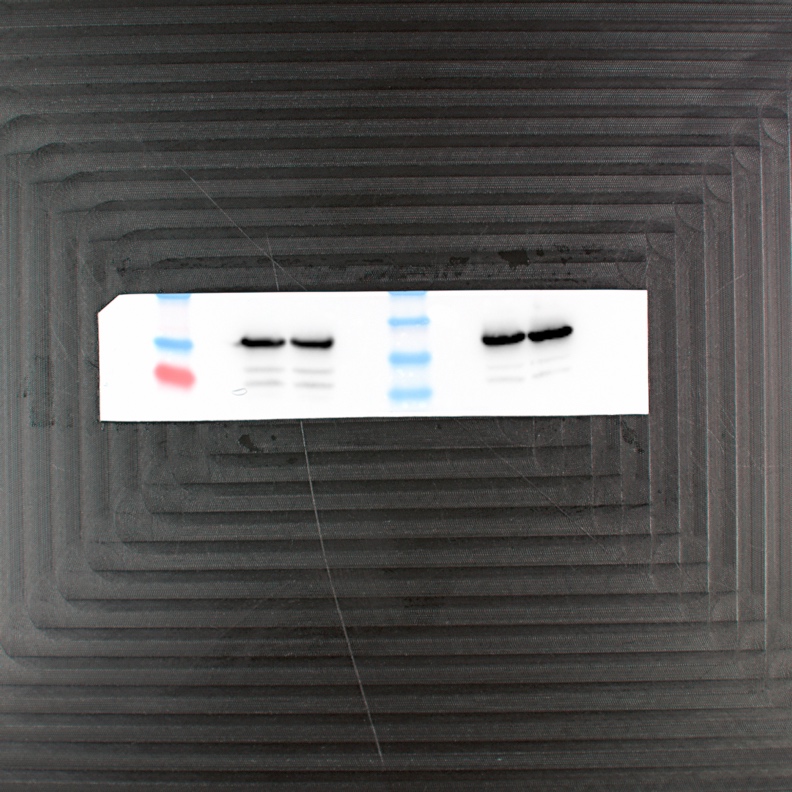


**Supplementary Figure 1C**


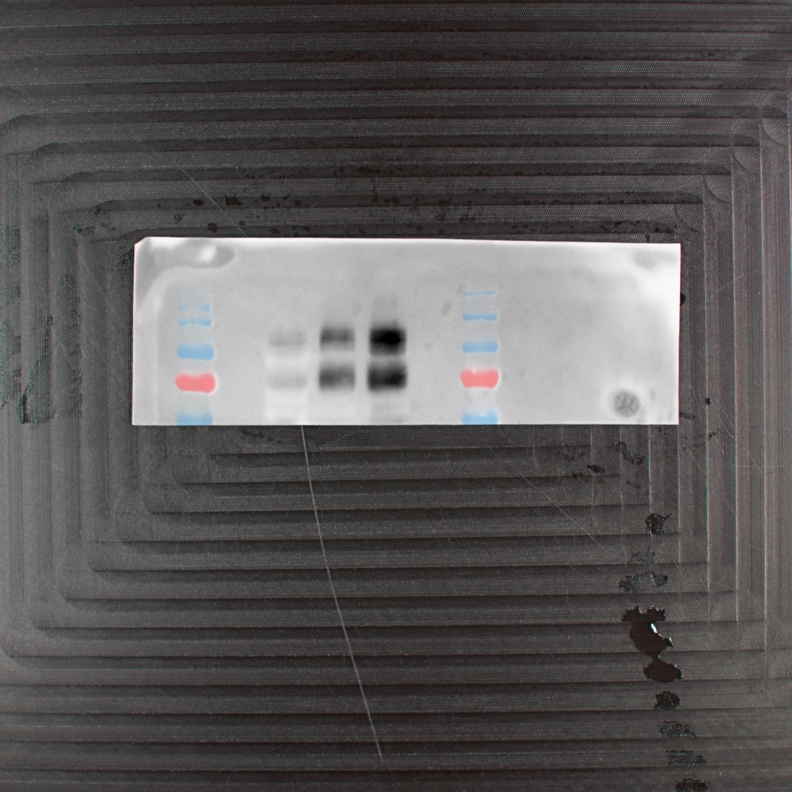

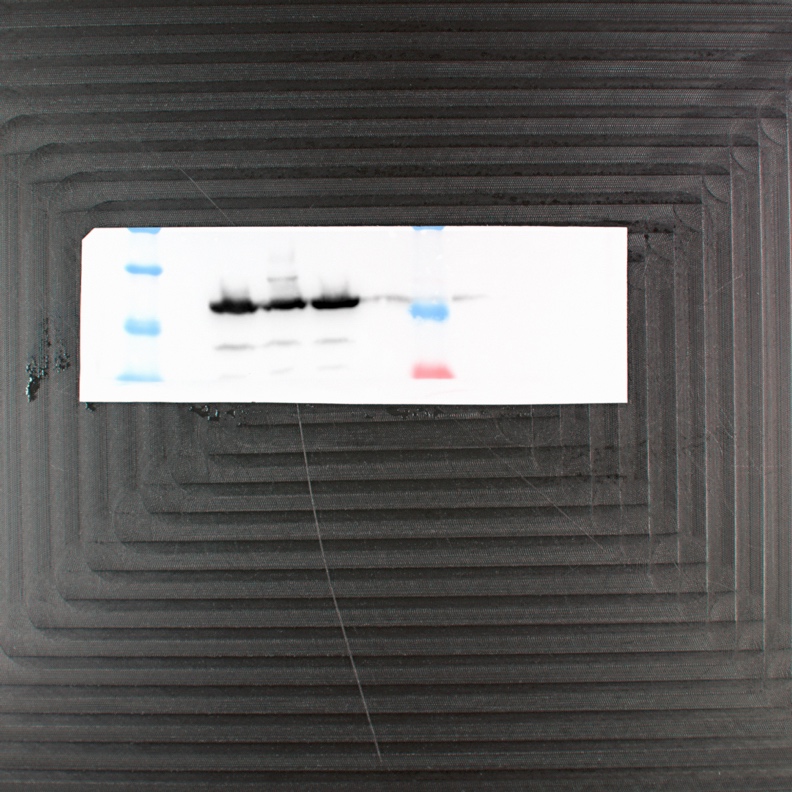


**Supplementary Figure 2A**


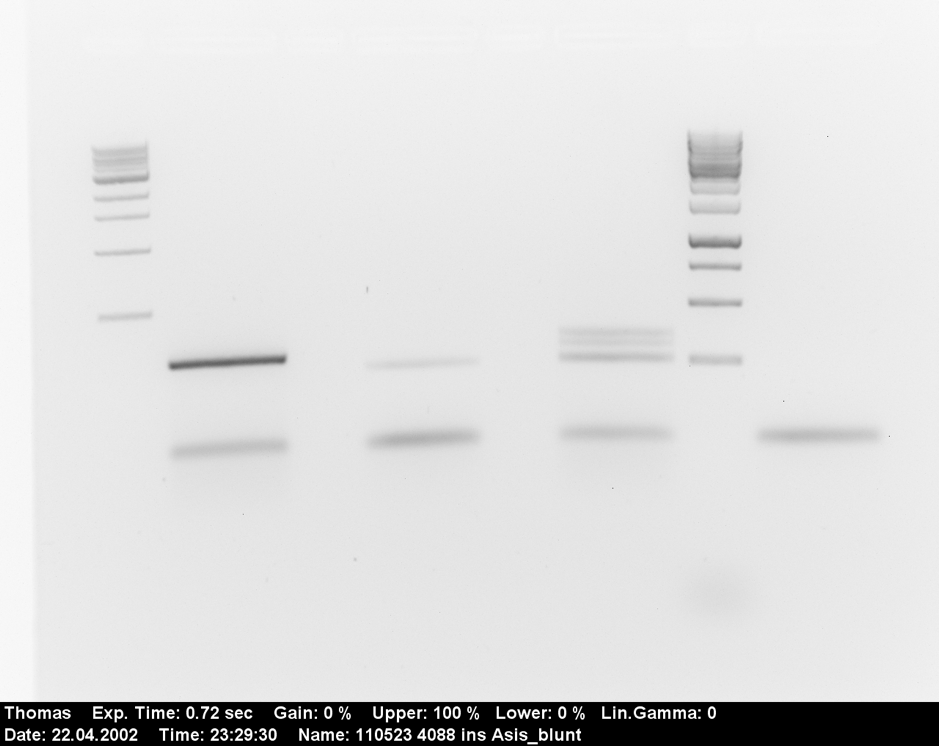


**Supplementary Figure 3B**

**
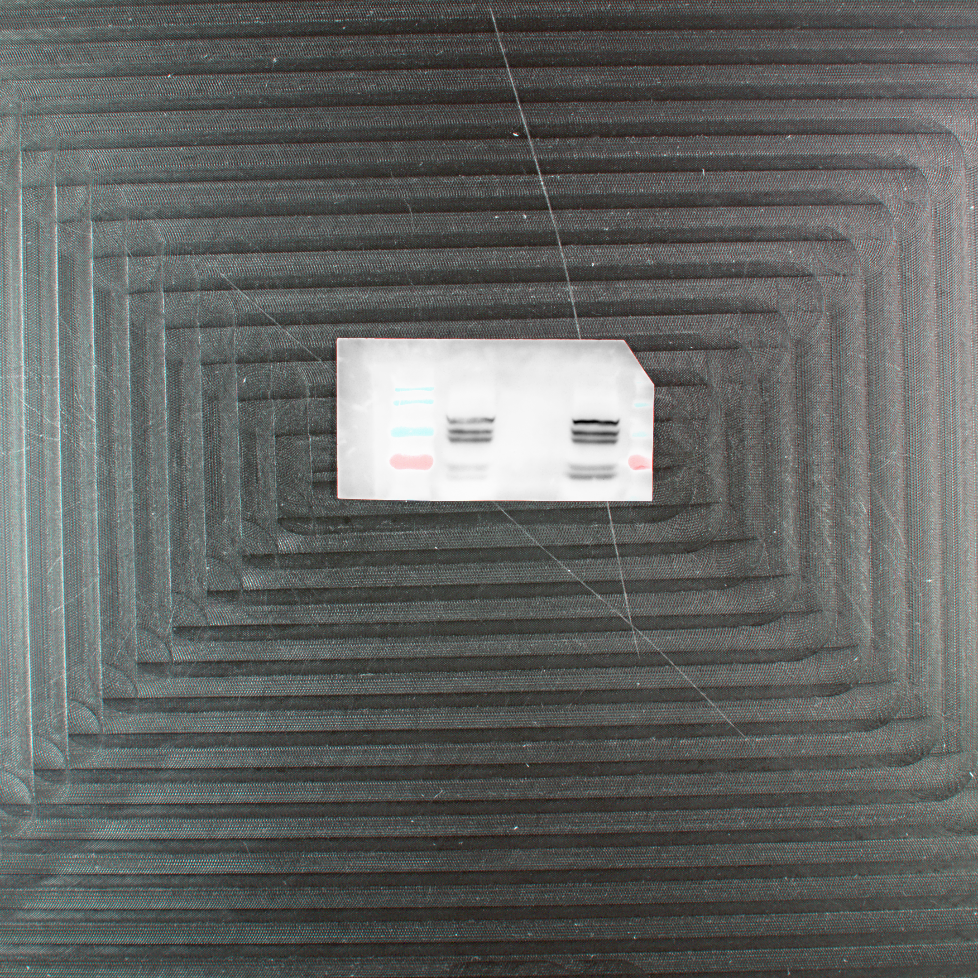
**

**
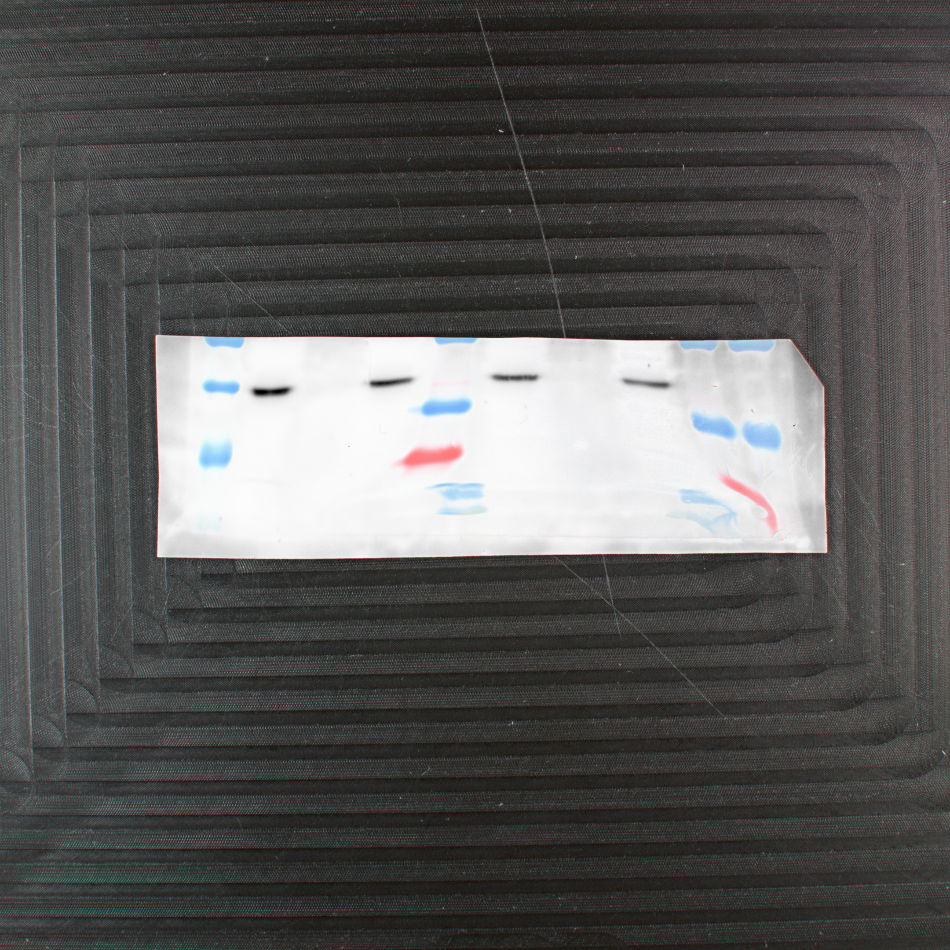
**

**Supplementary Figure 4B**


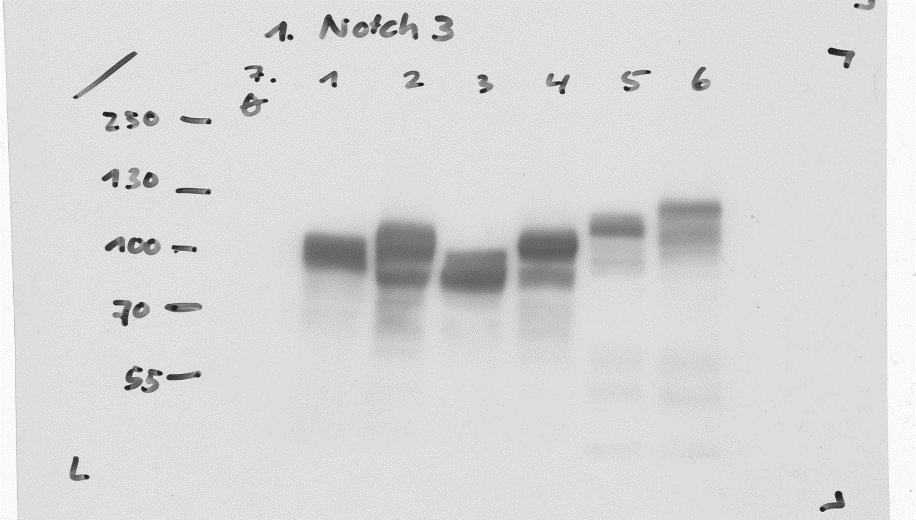


**Supplementary Figure 6B**


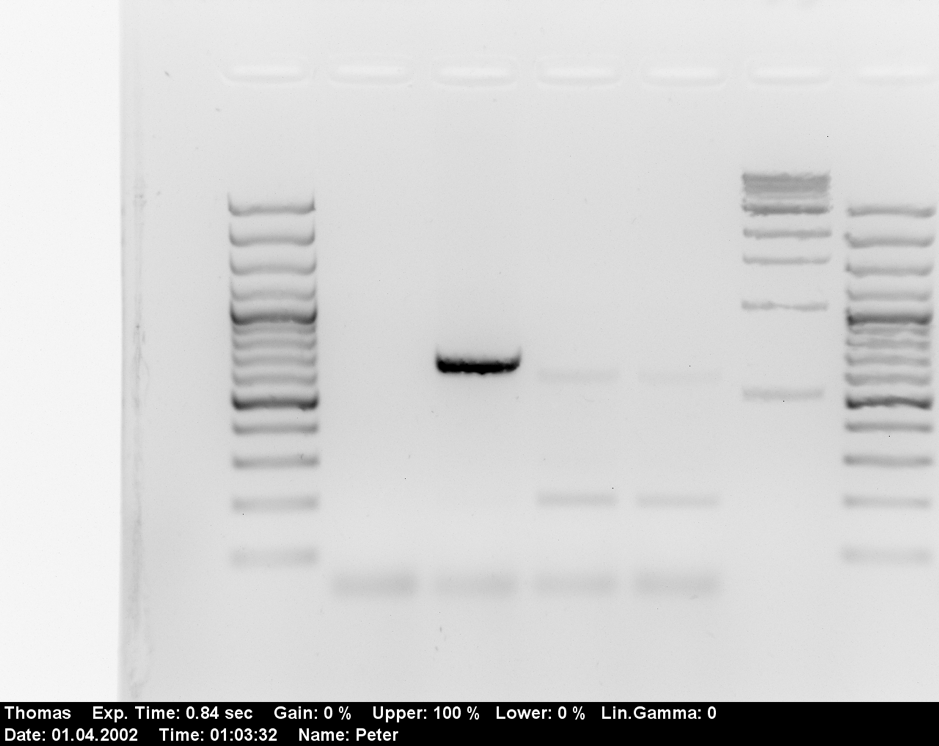


**Supplementary Figure 9B**


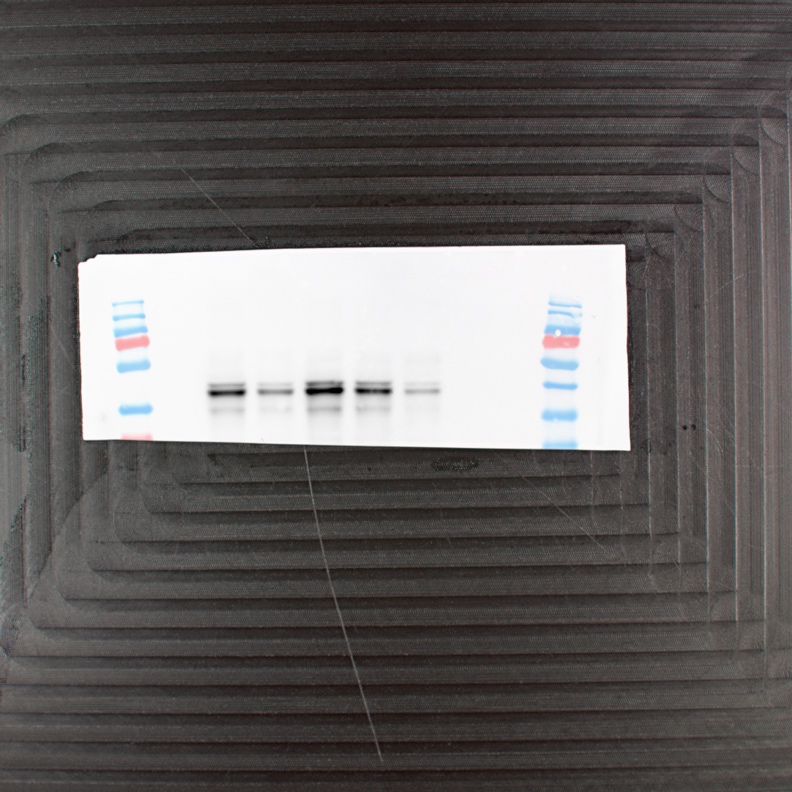

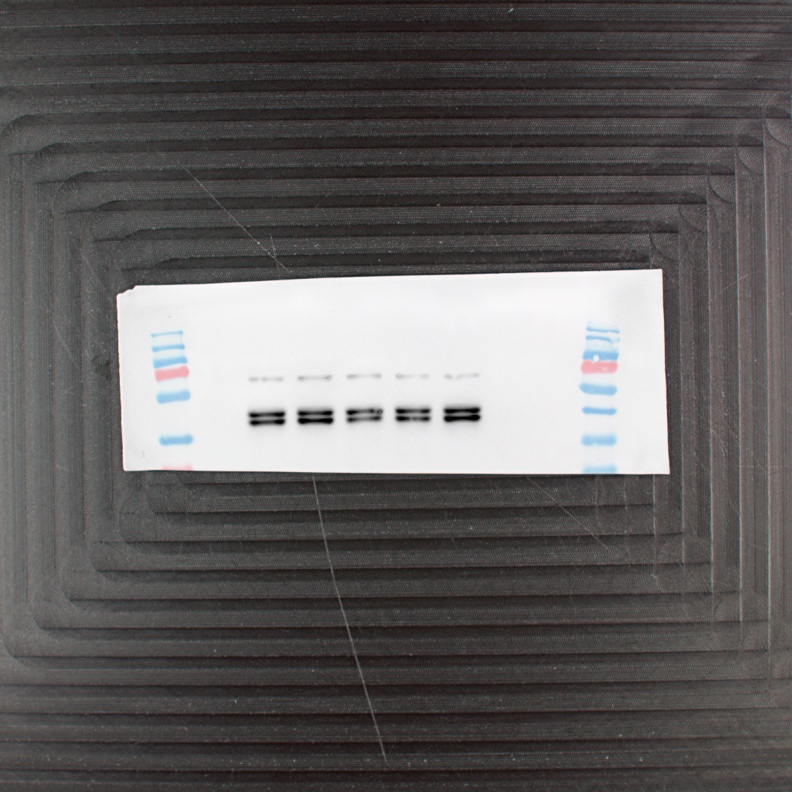

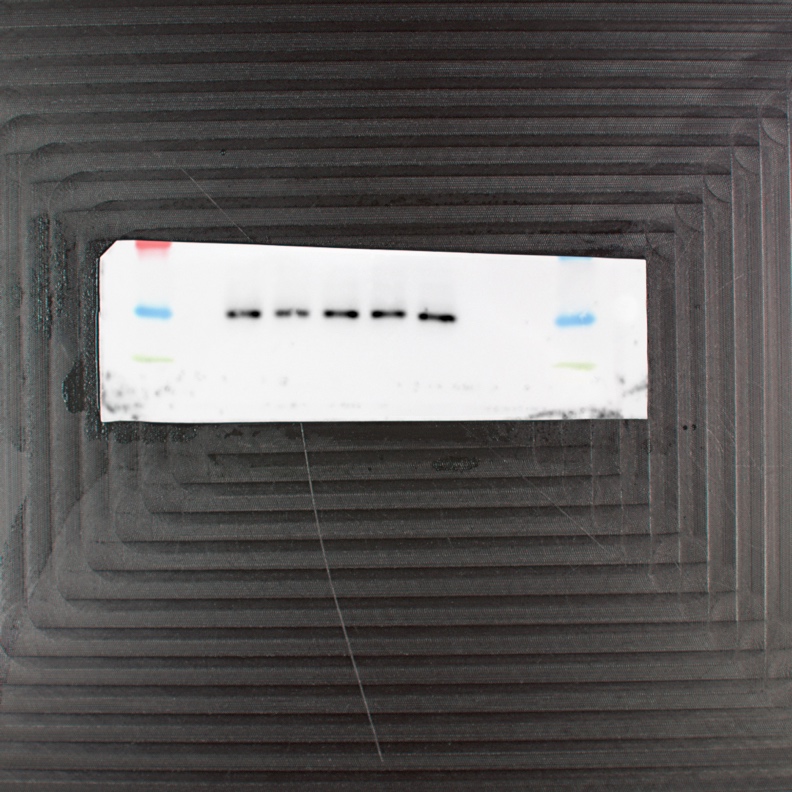


**Supplementary Figure 10A**


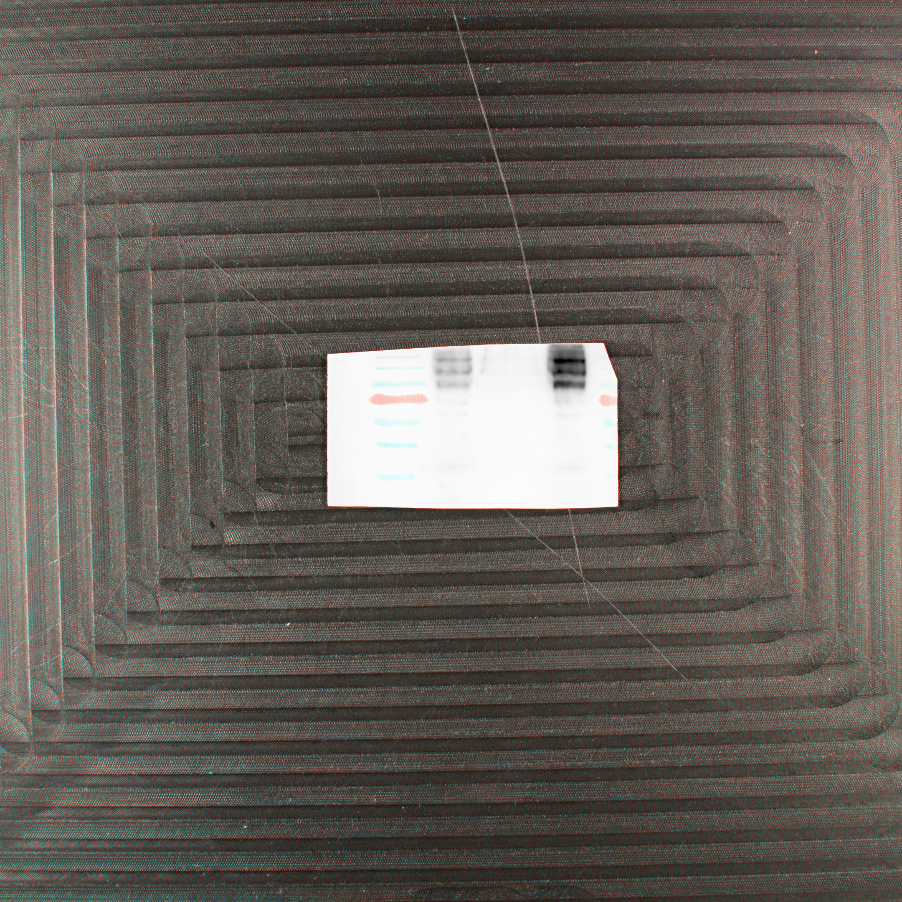


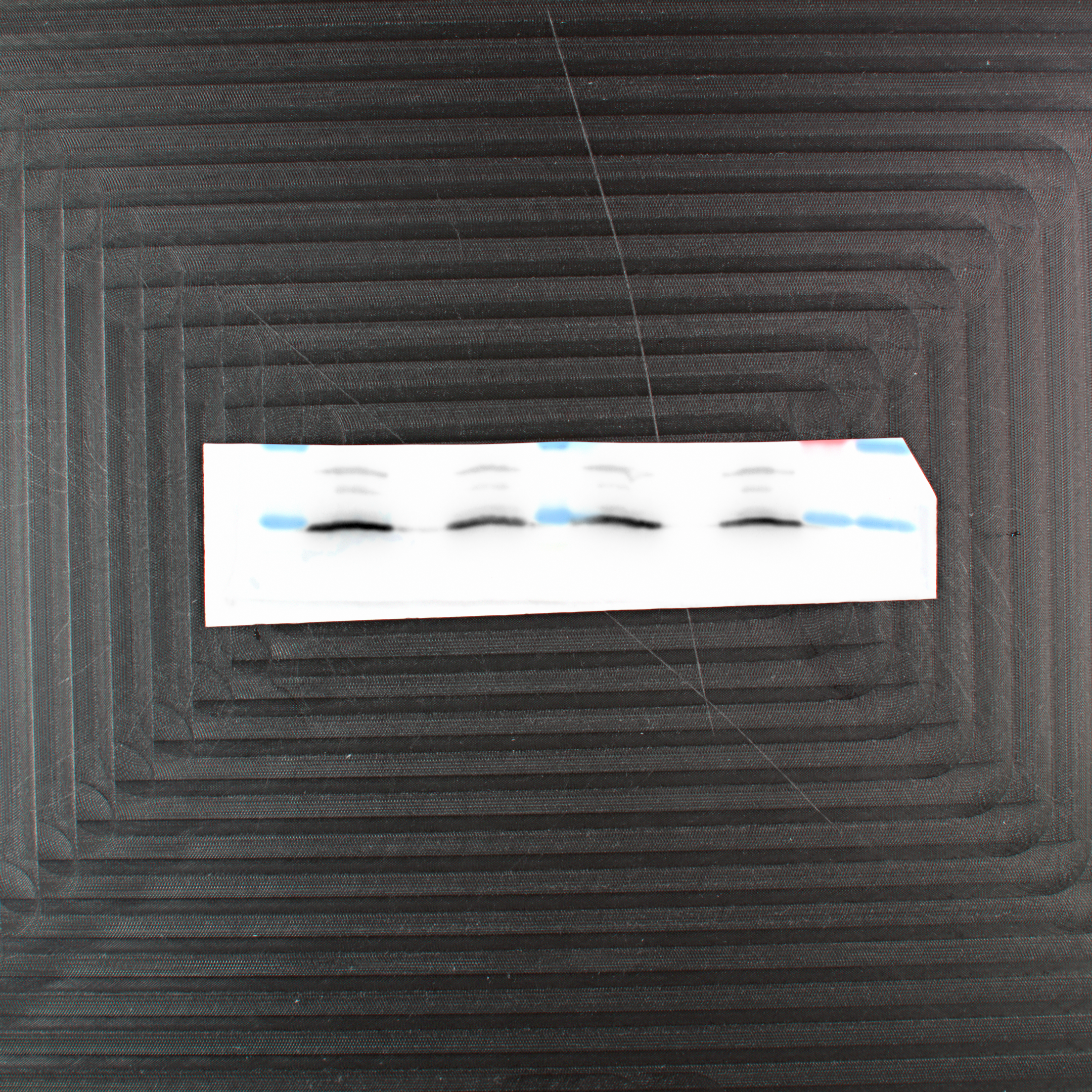

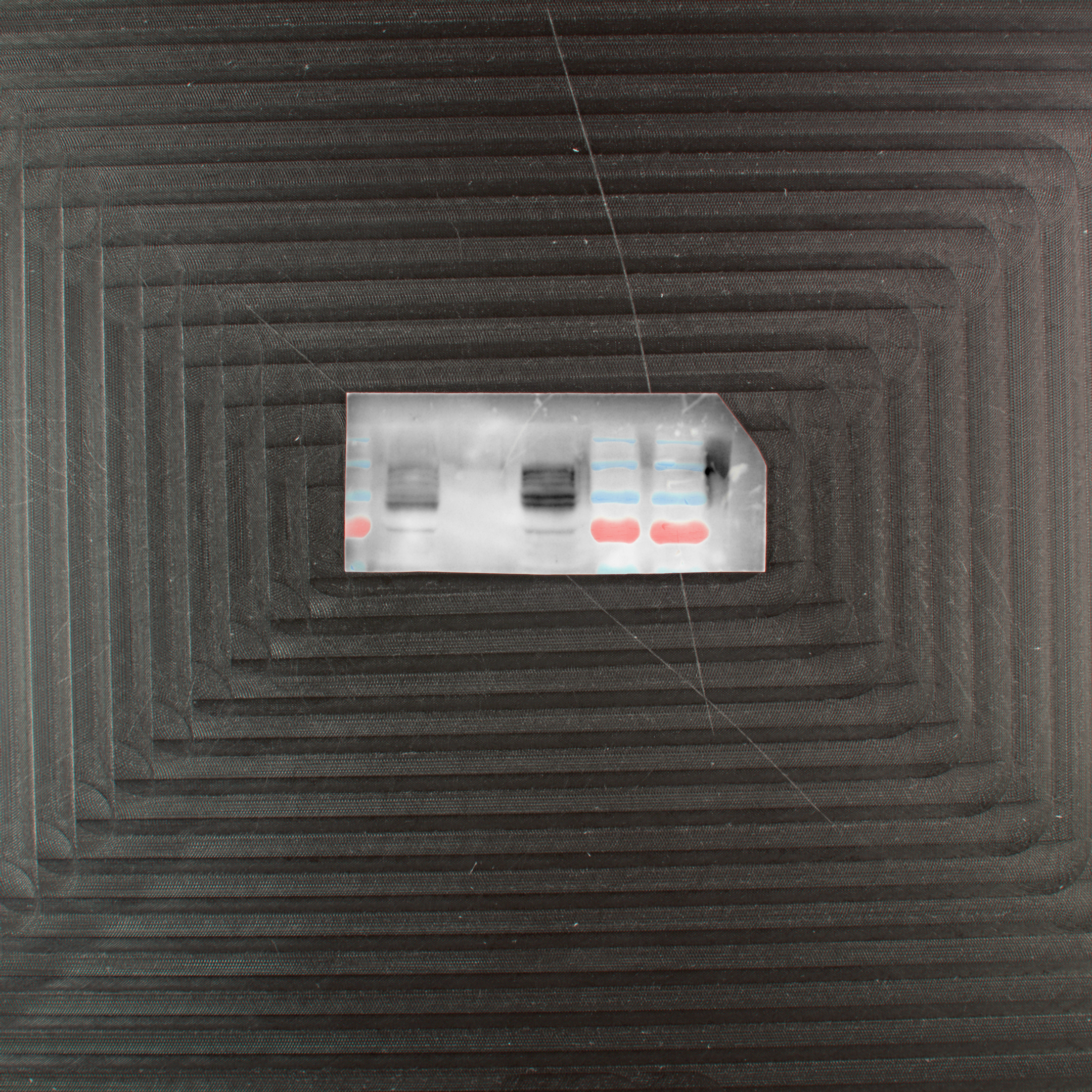


**
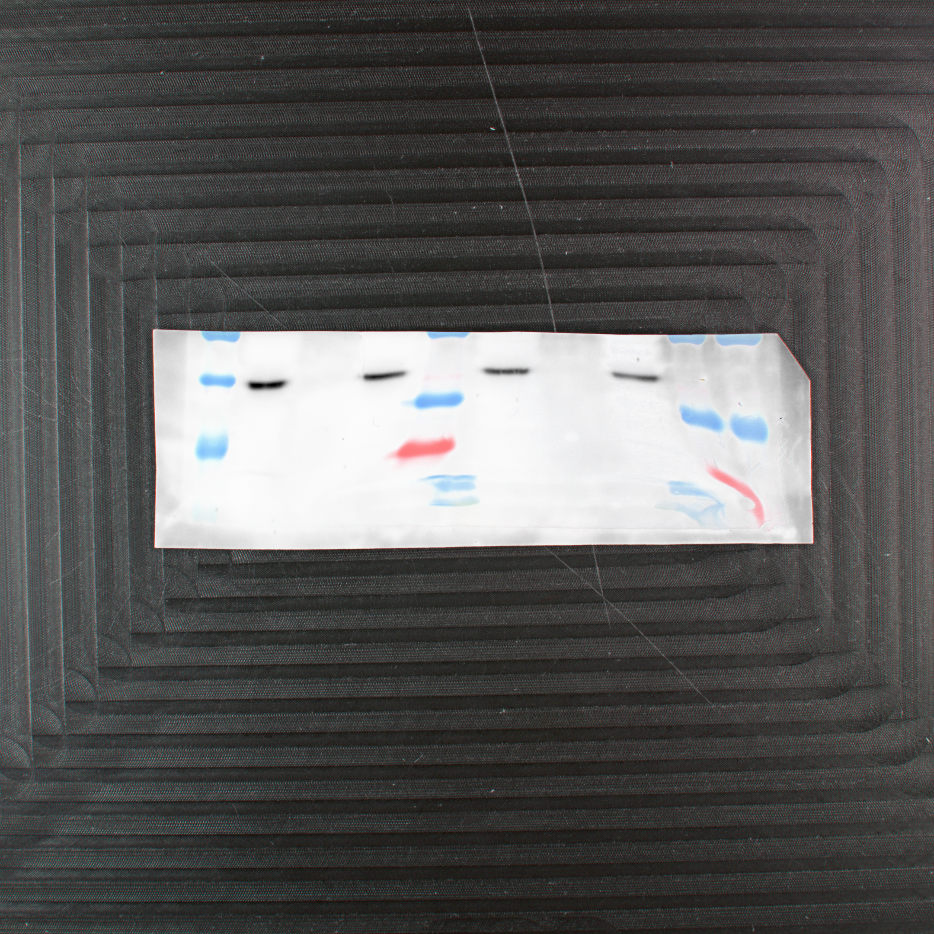
**


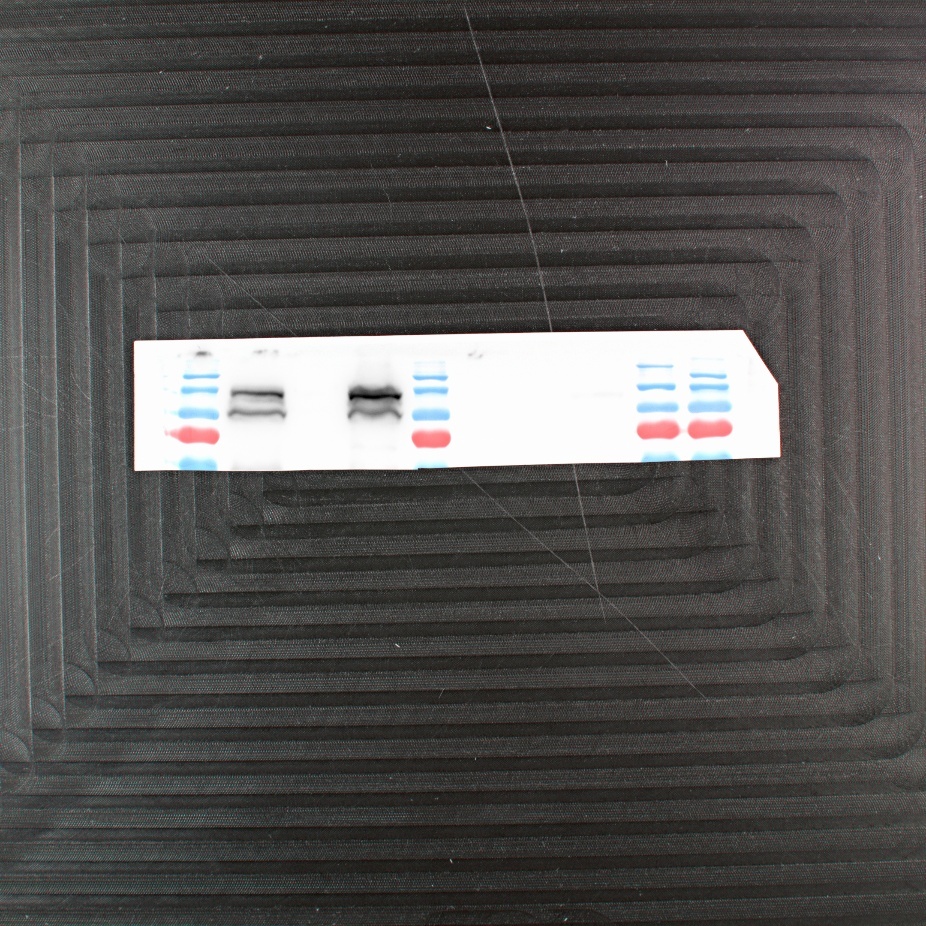


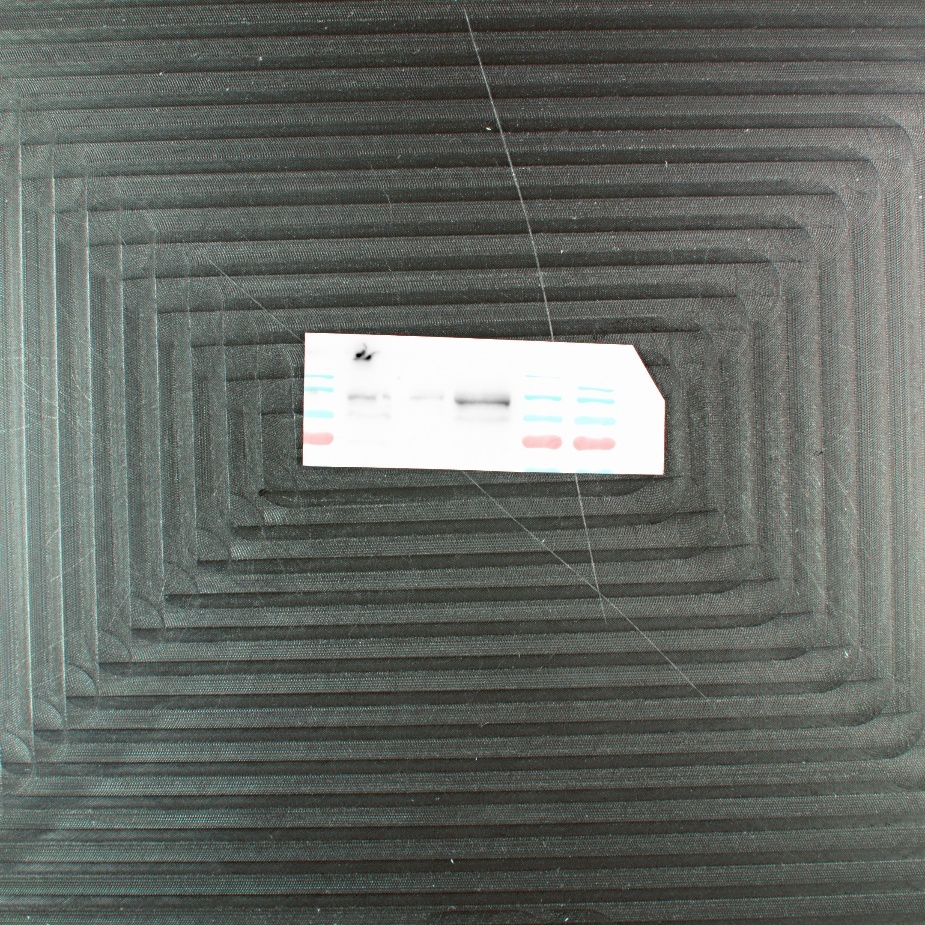


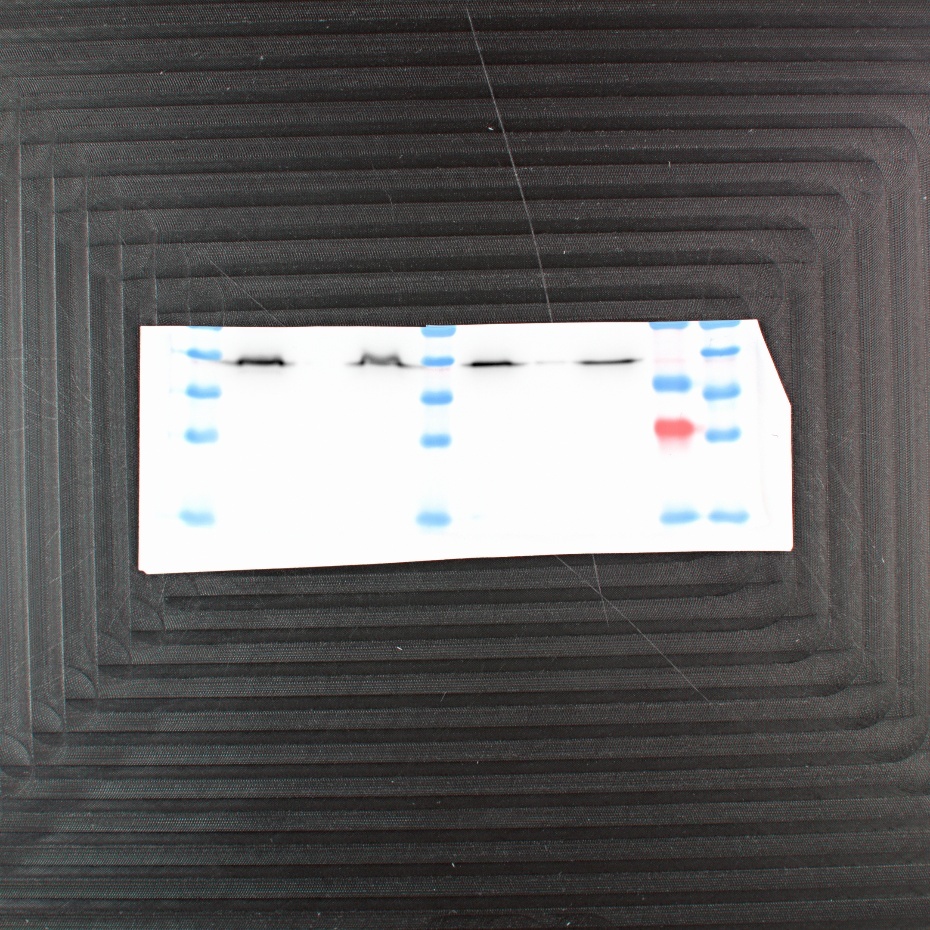


**Supplementary Figure 11**


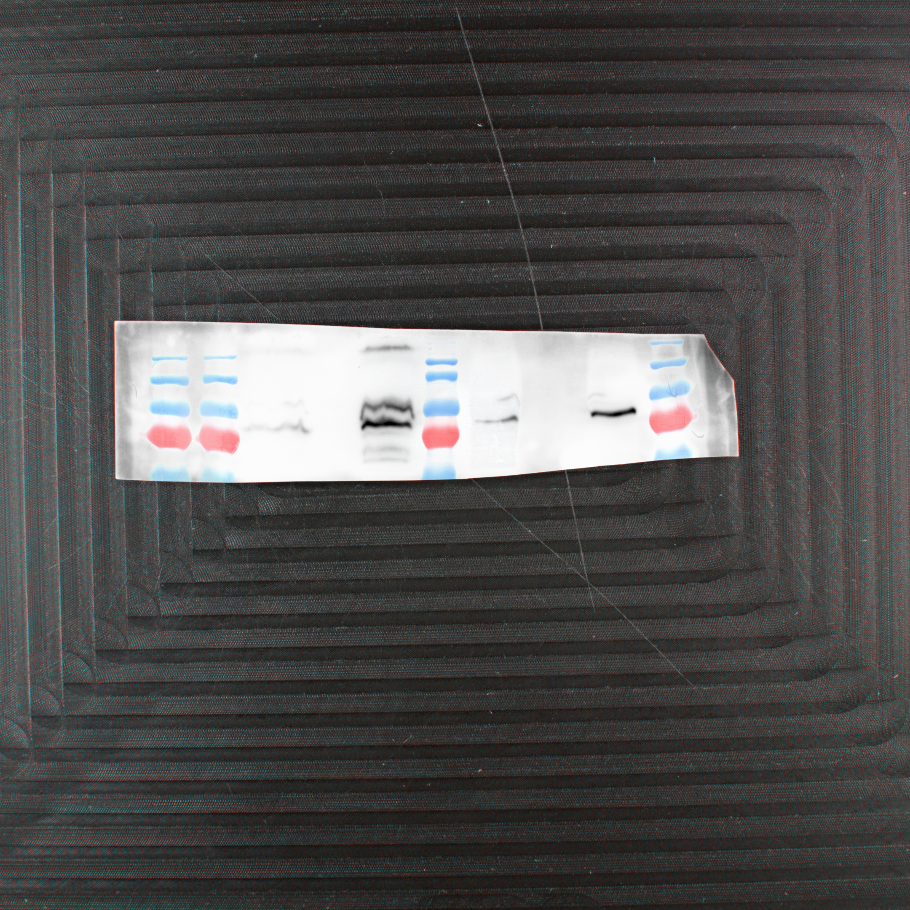


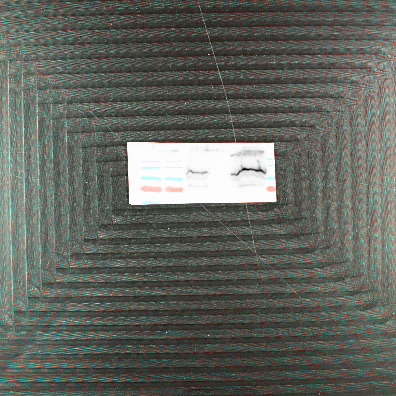


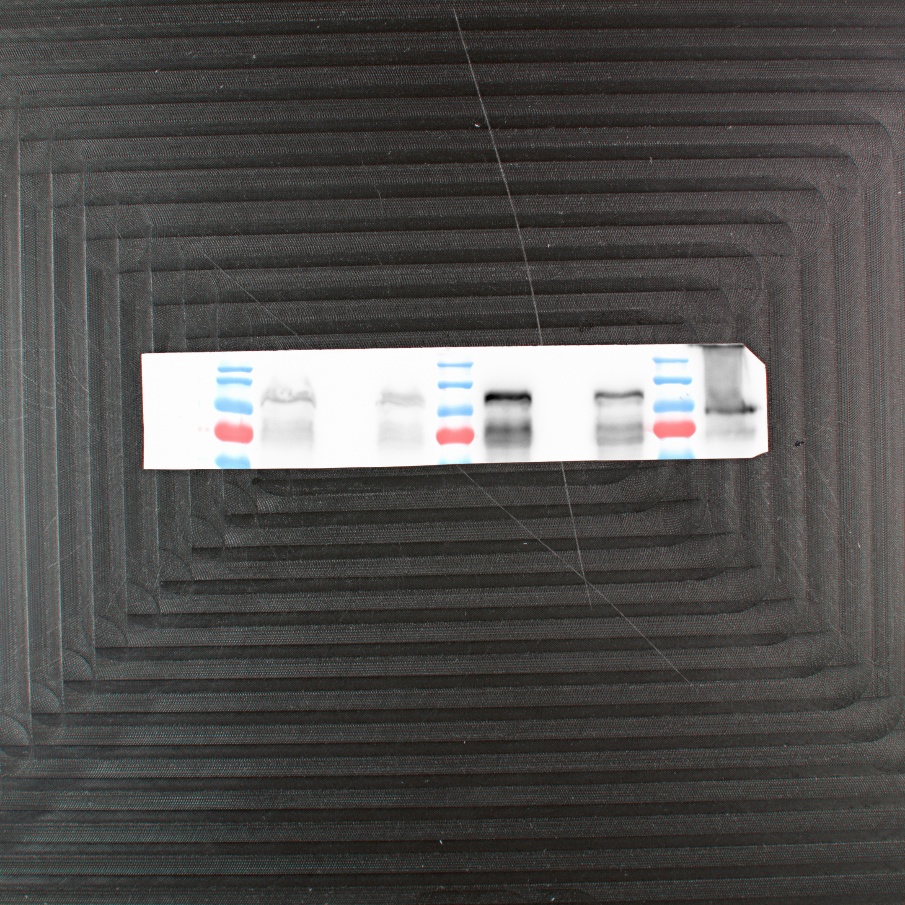


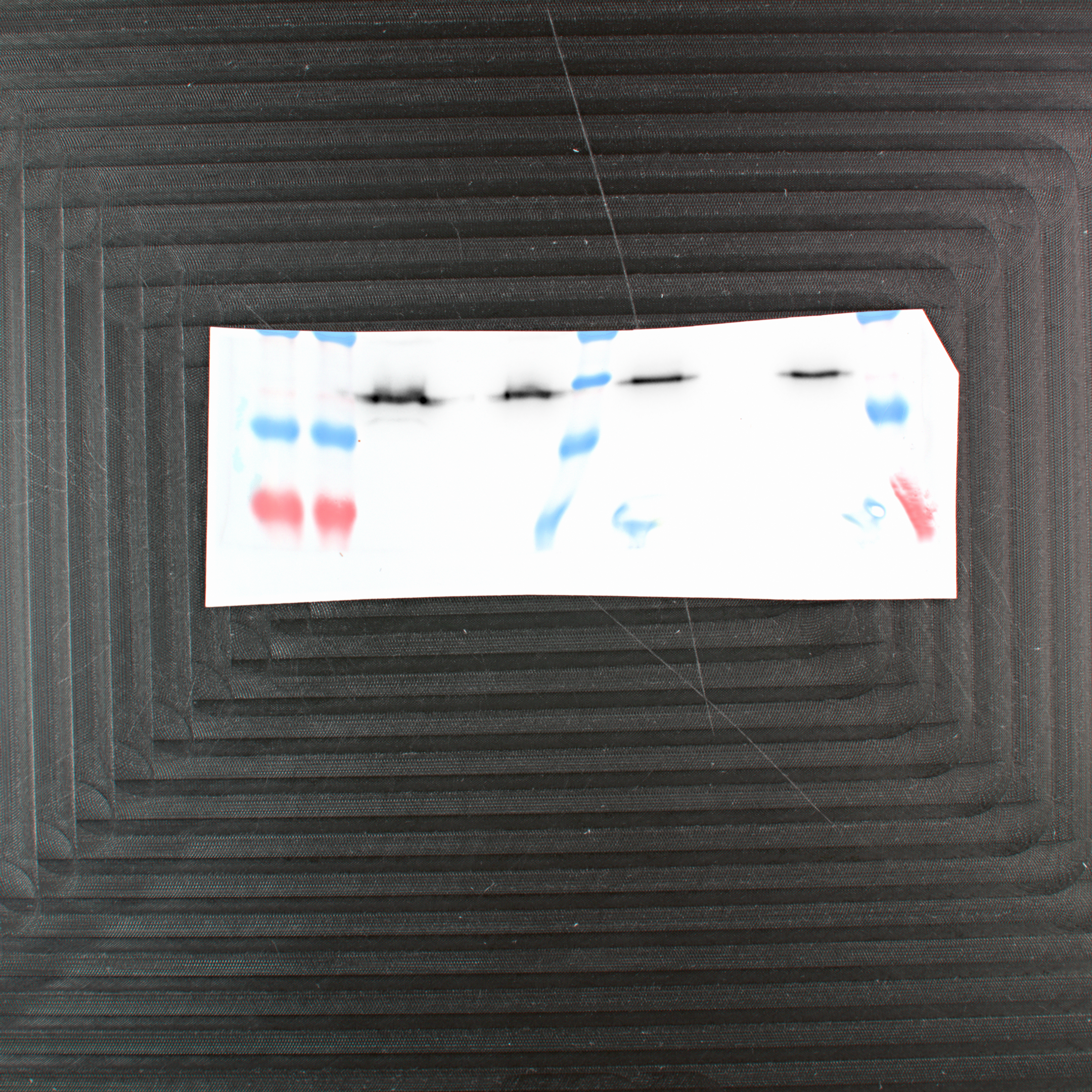


**Supplementary Figure 12B**


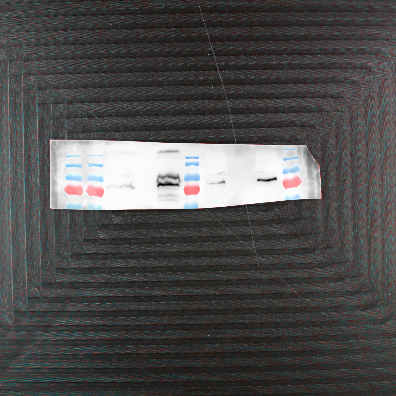


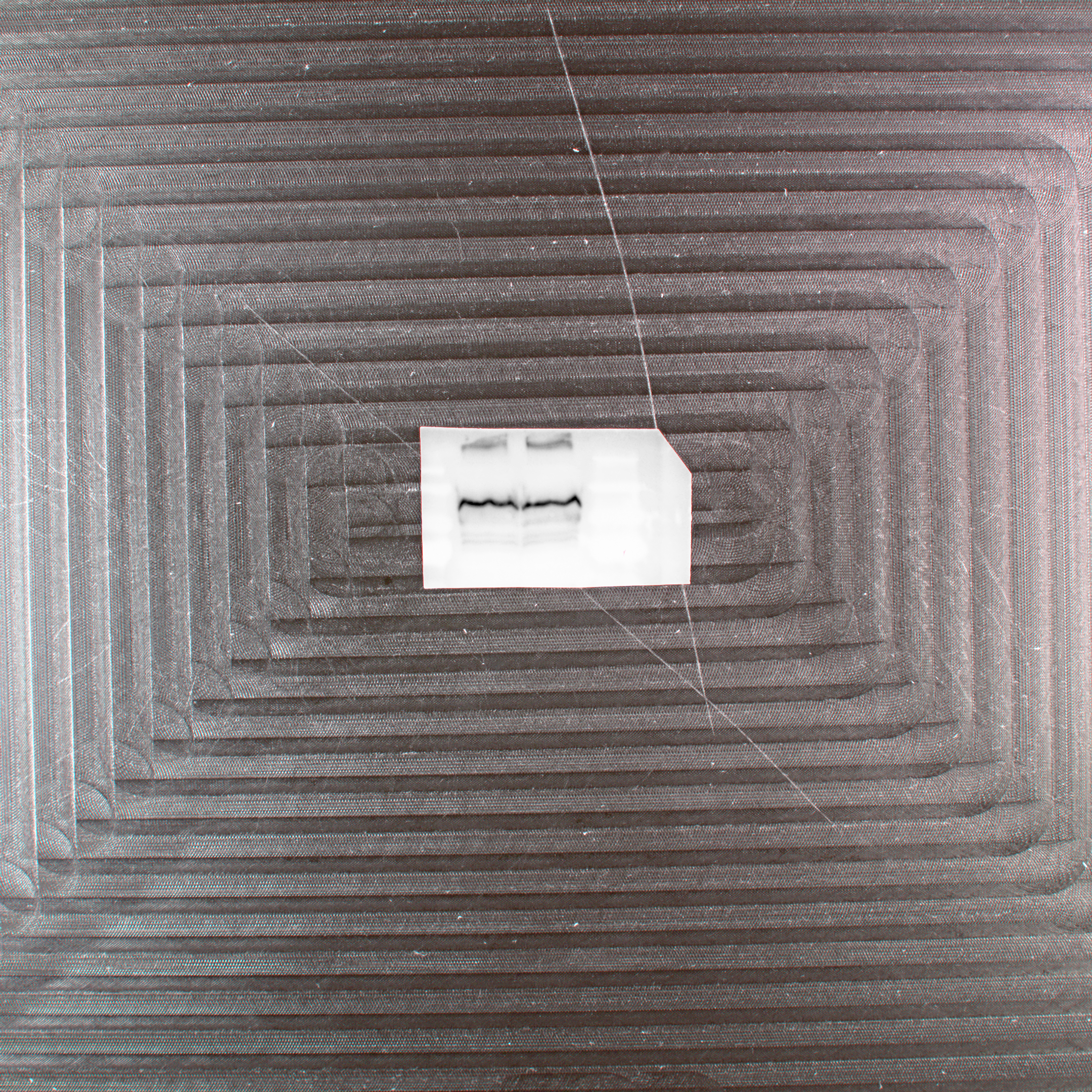


**
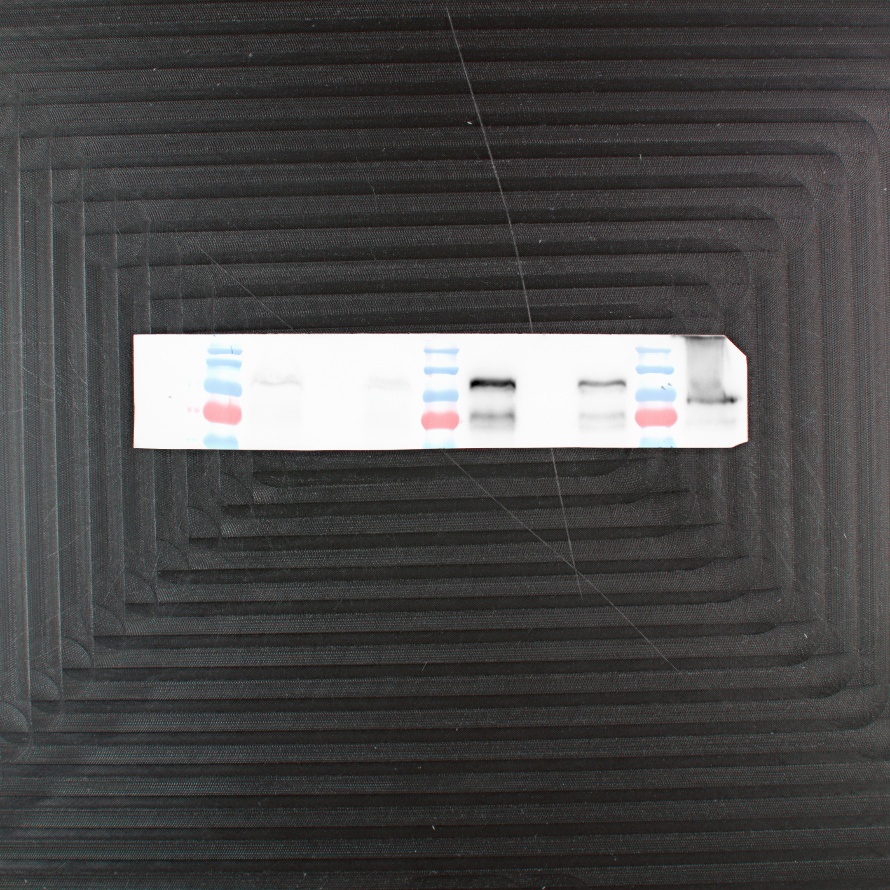
**


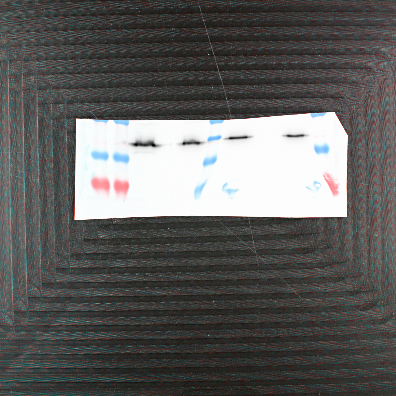

Supplement: Supplementary file 6 — source files_Western_blots [file 41419_2026_9044_MOESM6_ESM.docx]
